# High-efficiency base editing in the retina in primates and human tissues

**DOI:** 10.1038/s41591-024-03422-8

**Published:** 2025-01-08

**Authors:** Alissa Muller, Jack Sullivan, Wibke Schwarzer, Mantian Wang, Cindy Park-Windhol, Pascal W. Hasler, Lucas Janeschitz-Kriegl, Mert Duman, Beryll Klingler, Jane Matsell, Simon Manuel Hostettler, Patricia Galliker, Yanyan Hou, Pierre Balmer, Tamás Virág, Luis Alberto Barrera, Lauren Young, Quan Xu, Dániel Péter Magda, Ferenc Kilin, Arogya Khadka, Pierre-Henri Moreau, Lyne Fellmann, Thierry Azoulay, Mathieu Quinodoz, Duygu Karademir, Juna Leppert, Alex Fratzl, Georg Kosche, Ruchi Sharma, Jair Montford, Marco Cattaneo, Mikaël Croyal, Therese Cronin, Simone Picelli, Alice Grison, Cameron S. Cowan, Ákos Kusnyerik, Philipp Anders, Magdalena Renner, Zoltán Zsolt Nagy, Arnold Szabó, Kapil Bharti, Carlo Rivolta, Hendrik P. N. Scholl, David Bryson, Giuseppe Ciaramella, Botond Roska, Bence György

**Affiliations:** 1 Institute of Molecular and Clinical Ophthalmology Basel, Basel, Switzerland; 2 Department of Ophthalmology, University of Basel, Basel, Switzerland; 3 Beam Therapeutics, Cambridge, MA USA; 4 Department of Anatomy, Histology and Embryology, Semmelweis University, Budapest, Hungary; 5 SILABE, Université de Strasbourg, Niederhausbergen, France; 6 Clinique Vétérinaire Agoravet, Strasbourg, France; 7 Department of Genetics and Genome Biology, University of Leicester, Leicester, UK; 8 Ocular and Stem Cell Translational Research Section, National Eye Institute, National Institutes of Health, Bethesda, MD USA; 9 Department of Clinical Research, University of Basel, Basel, Switzerland; 10 Nantes Université, CNRS, INSERM, L’institut du thorax, Nantes, France; 11 Nantes Université, CHU Nantes, Inserm, CNRS, SFR Santé, Inserm UMS 016, CNRS UMS 3556, Nantes, France; 12 Université de Nantes, CHU de Nantes, INSERM UMR 1089, Translational Gene Therapy for Genetic Diseases, Nantes, France; 13 Department of Ophthalmology, Semmelweis University, Budapest, Hungary; 14 European Vision Institute, Basel, Switzerland; 15 Present Address: Medical University of Vienna, Department of Clinical Pharmacology, Vienna, Austria

**Keywords:** Translational research, Targeted gene repair, Visual system

## Abstract

Stargardt disease is a currently untreatable, inherited neurodegenerative disease that leads to macular degeneration and blindness due to loss-of-function mutations in the *ABCA4* gene. We have designed a dual adeno-associated viral vector encoding a split-intein adenine base editor to correct the most common mutation in *ABCA4* (c.5882G>A, p.Gly1961Glu). We optimized *ABCA4* base editing in human models, including retinal organoids, induced pluripotent stem cell-derived retinal pigment epithelial (RPE) cells, as well as adult human retinal explants and RPE/choroid explants in vitro. The resulting gene therapy vectors achieved high levels of gene correction in mutation-carrying mice and in female nonhuman primates, with average editing of 75% of cones and 87% of RPE cells in vivo, which has the potential to translate to a clinical benefit. No off-target editing was detectable in human retinal explants and RPE/choroid explants. The high editing rates in primates show promise for efficient gene editing in other ocular diseases that are targetable by base editing.

## Abstract (web summary)

A base-editing approach optimized to target the retina shows high editing rates in a mouse model of Stargardt disease, as well as in nonhuman primates and ex vivo human retinal explants, paving the way for potential clinical applications.

## Main

Age-related macular degeneration and monogenic forms of macular dystrophies cause blindness. The macula is the central part of the retina containing the fovea, which enables high-resolution color vision in primates. Currently there is no therapy for macular degeneration that halts cell death in the retina and/or RPE cells. Monogenic forms of macular degeneration have juvenile onset and are more severe; affected patients progressively lose their ability to read, drive or recognize faces, and become blind at the center of the visual field.

**Fig. 1:**
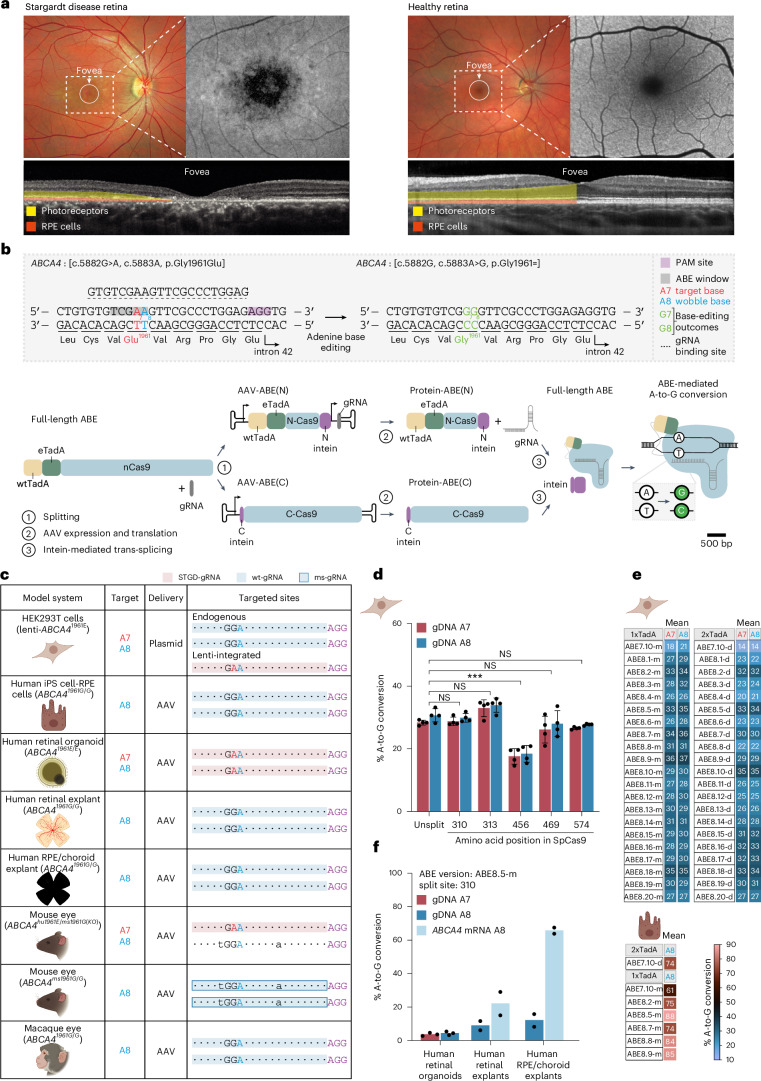
Adenine base editing corrects the most common Stargardt disease-associated mutation in vitro. **a**, Images of a retina of an individual with Stargardt disease with biallelic *ABCA4* mutations (c.5882G>A, p.Gly1961Glu); (c.66G>A, p.K = ) and of a healthy individual. The magnified grayscale images show the corresponding autofluorescence images. Decreased foveal autofluorescence (dark region) detected in the individual indicates atrophy of RPE cells. OCT images (bottom) show a cross-sectional view of the retina. The photoreceptor and RPE layers are highlighted to indicate foveal thinning. **b**, Dual AAV split-intein adenine base-editing strategy. Two adenines fall in the base-editing window: the c.5882A target base and the c.5883A wobble base of codon 1961. Conversion of the wobble base results in a silent base change. WtTadA and eTadA denote wild-type and evolved tRNA adenosine deaminase, respectively. **c**, Model systems used in the evaluation of *ABCA4* base-editing efficiency in photoreceptors and RPE cells. Genotype of the model systems, target adenines, delivery modalities and the targeted sites are indicated. The colors in the fourth column highlight the model-specific gRNA. STGD-gRNA, Stargardt disease gRNA; wt-gRNA, wild-type gRNA; ms-gRNA, mouse gRNA. Illustrations in **c**–**e** and illustrations of the AAV inverted terminal repeats (ITRs) created with BioRender.com. **d**, Base-editing efficiencies at the A7 and A8 sites with ABE7.10 split at five different positions in lenti-*ABCA4*^*1961E*^ HEK293T cells. Results were obtained from four biological replicates and presented as the mean ± s.d. ***P < 0.001, by three-way mixed-effects analysis of variance (ANOVA) with Dunnett’s correction, compared to the unsplit ABE7.10 construct. NS, not significant. **e**, Base-editing efficiencies at the A7 and A8 sites with different ABE versions in lenti-*ABCA4*^*1961E*^ HEK293T cells (top). Results were obtained from three replicates and are presented as means. Dual AAV-mediated base-editing efficiencies at the A8 site in gDNA of human iPS cell-RPE cells (bottom). Results were obtained from two replicates and are presented as means. **f**, AAV-mediated base-editing in gDNA and in *ABCA4* mRNA of different in vitro models using an ABE8.5m base editor split at amino acid residue 310 of SpCas9 (total dose: 2.3 × 10^11^ vector genomes (v.g.) per organoid and 3.3 × 10^11^ v.g. per human tissue, 1:1 ratio of ABE(N) and ABE(C)). Results were obtained from two to three replicates and are presented as means.

**Extended Data Fig. 1:**
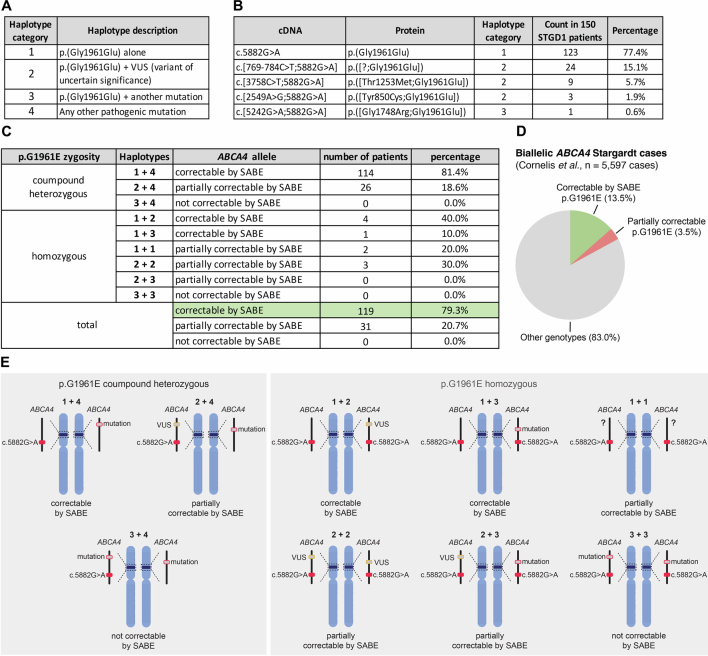
c.5882G>A haplotype categories and percent of patients who have a fully correctable allele by SABE. Our analysis is based on published genetic data from 150 Stargardt patients mostly from European descent^6^. **A**) Possible haplotypes found in Stargardt patient with at least one allele including c.5882G>A. **B**) Counts and percentage of different haplotypes found in the published study. **C**) Fraction of patients who have at least one allele, which is fully or partially correctable by SABE. **D**) Pie chart showing fraction of STGD patients carrying the p.Gly1961Glu mutation who have at least one allele, which is fully (green) or partially (red) correctable by SABE. The calculation is based on Lee W et al.^6^ and was applied on a large number of patients analyzed by Cornelis SS et al^50^. **E**) Depiction of possible *ABCA4* genotypes with respect to SABE-mediated correction. Concerning patients with homozygous haplotype 1, we expect other yet unknown modifier(s) (labeled with question mark) since homozygosity for c.5882G>A alone was shown not to lead to disease^51^. Therefore, we considered haplotype 1 as “partially correctable”. VUS, variant of uncertain significance.

The most common monogenic form affecting 1 in 6,500 individuals is Stargardt disease^1^ (Fig. 1a), which is caused by biallelic loss-of-function mutations in the *ABCA4* gene. The ABCA4 protein is a membrane lipid flippase that is localized in photoreceptors and RPE cells and prevents the accumulation of toxic retinoids in the retina that lead to cell death^2–4^. The most common Stargardt disease-associated mutation, affecting 15% of patients^5^, is a G-to-A point mutation in *ABCA4* (c.5882G>A, p.Gly1961Glu) that causes disease when present in *trans* with another more severe *ABCA4* mutation^6^. In the majority of individuals of European descent (79%; Extended Data Fig. 1), no additional pathogenic variants have been identified on the same allele with the *ABCA4* c.5882G>A mutation. This suggests that gene correction of the c.5882G>A in these individuals is therapeutically relevant. The mutation leads to decreased transporter activity of ABCA4 (ref. ^7^) and affected individuals demonstrate a characteristic foveal atrophy, mostly without lipid flecks (Fig. 1a).

DNA base editors, which include adenine base editors^8^ (ABEs) and cytosine base editors^9^, are precision gene-editing tools that allow targeted repair of single-nucleotide mutations without inducing double-stranded DNA breaks. ABEs include a Cas9 nickase (nCas9) fused to an evolved tRNA adenosine deaminase (TadA; Fig. 1b) that is targeted to the genomic DNA (gDNA) site of interest when complexed with a guide RNA (gRNA). ABEs convert A:T base pairs to G:C base pairs in the DNA through deamination in a short editing window. Base editing is, therefore, a plausible approach to correct the c.5882G>A mutation (Fig. 1b).

Although there are reports showing the potency of base editing in cell lines and in some cases in mice^10–15^, high levels of gene correction have yet to be demonstrated in the nervous tissue of humans and nonhuman primates (NHPs). Here we developed an adeno-associated viral vector (AAV)-based adenine base-editing strategy to correct the *ABCA4* c.5882G>A mutation in therapeutically relevant cell types, and we provide proof-of-concept for high-efficiency gene correction in human retina and RPE in vitro, as well as in mice and NHPs in vivo.

## Results

### Base-editing approach for the most common *ABCA4* mutation

**Extended Data Fig. 2:**
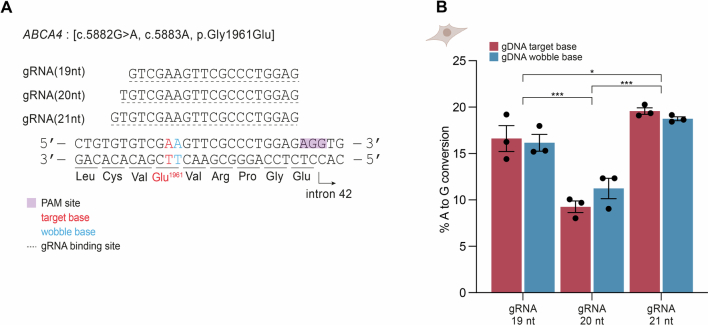
Adenine base-editing efficiency by different gRNAs. **A**) Schematic of a fragment of the *ABCA4* exon 42 with the NGG-PAM sequence indicated in purple, the Stargardt c.5882A target base highlighted in red, and the c.5883A wobble base shown in blue. **B**) Base-editing efficiencies at the target- and wobble bases with the unsplit ABE7.10 base editor in combination with the different gRNAs in lenti-*ABCA4*^1961E^ HEK293T cells. Results were obtained from three biological replicates (eyes) and are presented as mean ± s.d. *P < 0.05, ***P < 0.001 by three-way mixed-effect ANOVA with Tukey’s correction. Illustration in **b** created with BioRender.com.

To develop an adenine base-editing strategy to correct the c.5882G>A mutation, we first designed and tested different gRNAs in combination with ABE7.10 (ref. ^8^) in a HEK293T cell line carrying the mutation on a lentivirus insert (lenti-*ABCA4*^*1961E*^ HEK293T; Extended Data Fig. 2). Although the target mutation in HEK293T cells is not in the endogenous chromatin context, it allowed us to prescreen editors rapidly. We selected a 21-nucleotide gRNA that corrected the target site with the highest efficiency, referred to as Stargardt-gRNA (STGD-gRNA) (Supplementary Table 1). STGD-gRNA places the c.5882A target base of codon 1961 at position 7 (A7) inside the base-editing window (Fig. 1b).

**Extended Data Fig. 3:**
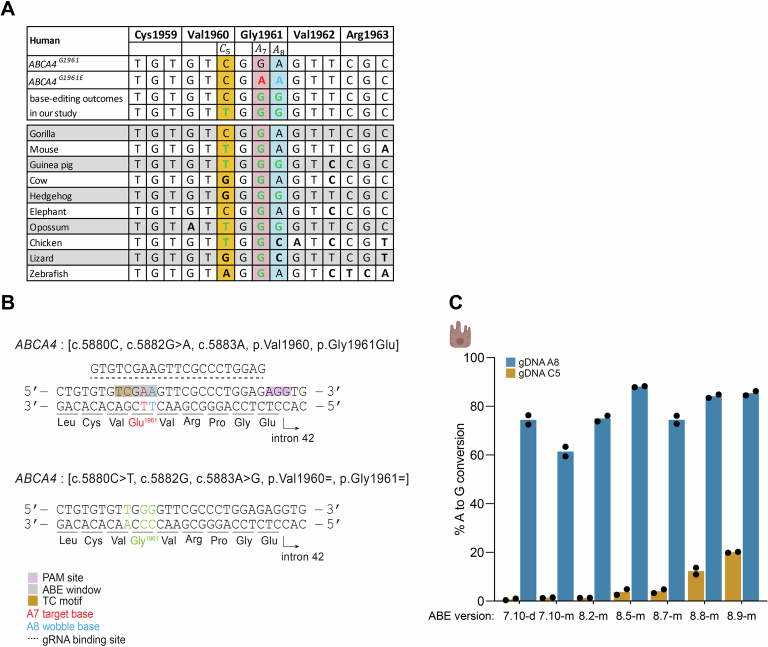
Conservation of the *ABCA4* sequence around p.Gly1961 and ABE-mediated cytosine editing on the *ABCA4* gene. **A**) The table shows the sequence alignment between humans and 10 other vertebrates. The first row shows the human *ABCA4* reference sequence. All sequence changes to the human sequence are indicated in bold. The second row shows the *ABCA4* c.5882A allele, with the A7 target base highlighted in red. The third and fourth rows show the most frequent base-editing outcomes in our study. The two observed bystander edits, c.5880C to c.5880T at position five (c.5880 C>T, p.Val1960 = ) and c.5883A to c.5883G at position eight (c.5883A>G, p.Gly1961=) lead to silent changes, do not affect conserved base positions, and are present in other species. These results suggest that these bystander base changes have no biological relevance. **B**) Fragment of the *ABCA4* exon 42 with the base-editor window highlighted in grey. The base-editor window also contains a TC sequence that constitutes a possible motif for ABE-mediated cytosine editing. *ABCA4* c.5880C to c.5880T bystander editing results in a silent change (c.5880C>T, p.Val1960=). This change is not conserved and is expected to have no biological relevance. **C**) Adenine base-editing efficiencies at the A8 site and cytosine base-editing efficiencies at the C5 site with different dual AAV-ABE versions in human iPS cell-RPE cells. Results were obtained from two biological replicates and are presented as mean. Illustration in **c** created with BioRender.com.

**Extended Data Fig. 4:**
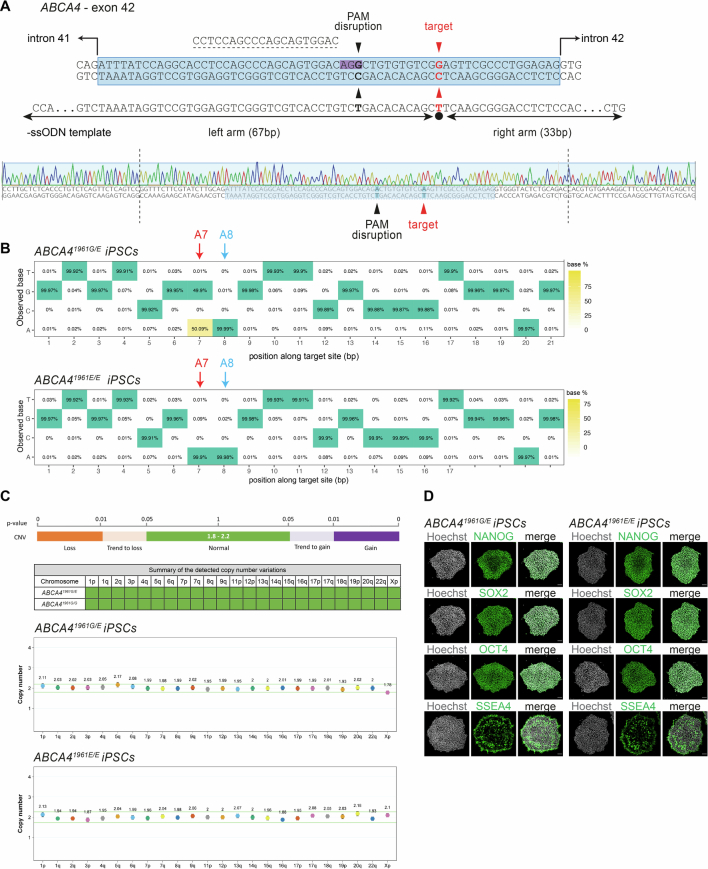
Generation of the *ABCA4*^*G1961E*^ human mutant iPSC line. **A**) Strategy for the generation of an *ABCA4*^*G1961E*^ iPSC line. *ABCA4* exon 42 (blue box) with flanking introns (top). The PAM site is highlighted in purple, and the gRNA binding site is indicated by a black dashed line. The black arrowheads point to the PAM disruption site (silent mutation) and the red arrowheads point to the *ABCA4* c.5882G>A mutation. Representative Sanger sequencing trace of the *ABCA4*^*1961E/E*^ clone that was selected for human retinal organoid induction (bottom). **B**) Results from targeted deep-sequencing of the *ABCA4*^*1961G/E*^ (top) and *ABCA4*^*1961E/E*^ (bottom) clone confirming successful knock-in of the target mutation in a heterozygous or homozygous form. **C**) Results from the iPSC digital aneuploidy test, confirming the genomic integrity of the *ABCA4*^*1961G/E*^ (top) and *ABCA4*^*1961E/E*^ (bottom) clone. These clones were used for human retinal organoid induction. **D**) Confocal images of *ABCA4*^*1961G/E*^ (left) and *ABCA4*^*1961E/E*^ (right) iPSCs. Green: antibody for pluripotency markers (NANOG, SOX2, OCT4, SSEA4); grey: Hoechst (scale bars: 100 µm). The experiment was performed once with one biological replicate.

**Extended Data Fig. 5:**
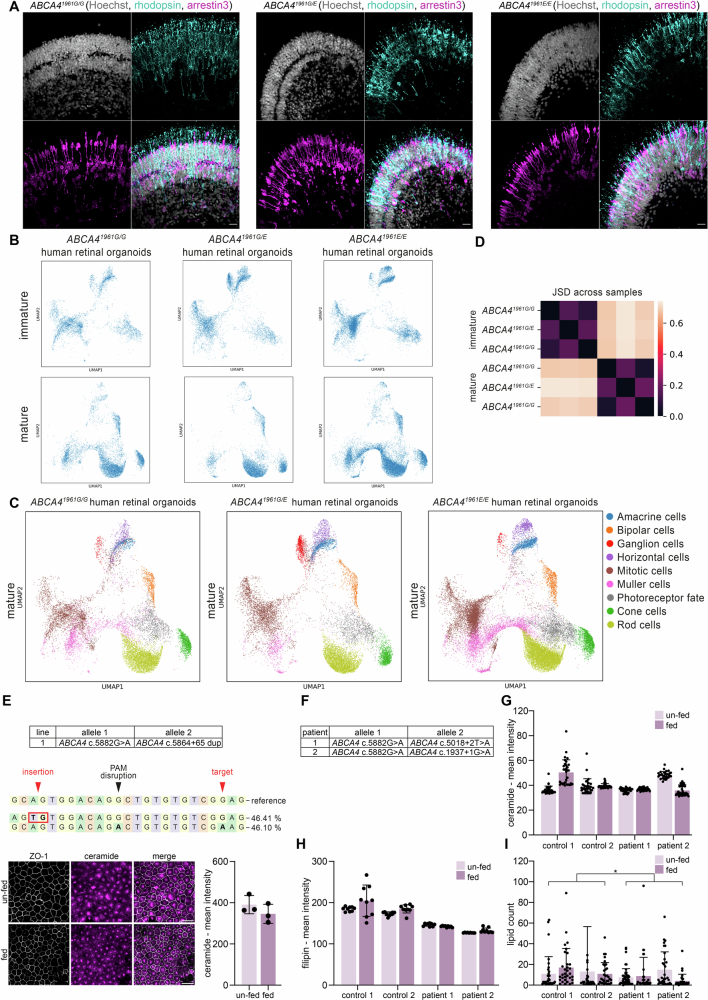
Characterization of the *ABCA4*^*G1961E*^ human retinal organoids. **A**) Confocal images of *ABCA4*^*1961G/G*^ (left), *ABCA4*^*1961G/E*^ (middle), and *ABCA4*^*1961E/E*^ (right) human retinal organoids. Grey: Hoechst; cyan: rhodopsin; magenta: arrestin3 (scale bars: 25 µm). **B**) 2D UMAP projection of single cells from human retinal organoids ordered by the *ABCA4* genotype (*ABCA4*^*1961G/G*^ human retinal organoids: left; *ABCA4*^*1961G/E*^ human retinal organoids: middle, and *ABCA4*^*1961E/E*^ human retinal organoids: right) and the developmental stage (immature: top; mature: bottom). **C**) 2D UMAP plot of scRNA data from mature human retinal organoids colored by cell type and plotted separately by the *ABCA4* genotype (*ABCA4*^*1961G/G*^ human retinal organoids: left; *ABCA4*^*1961G/E*^ human retinal organoids: middle, and *ABCA4*^*1961E/E*^ human retinal organoids: right). **D**) Heat map for Jensen–Shannon divergence (JSD) showing the similarity between organoids of different *ABCA4* genotypes at two different developmental stages (immature: top; mature: bottom). **E**) Results from targeted deep-sequencing of the *ABCA4*^*1961G/E*^ clone confirming successful knock-in of the target mutation in a heterozygous form. Confocal images of un-fed and POS-fed iPS cell-RPE cells. Grey: ZO-1; magenta: ceramide (scale bars: 25 µm). Quantification of ceramide intensity signal, for un-fed and POS-fed iPS cell-RPE cells. **F**) Genotypes of patient iPS cell-RPE cells. Quantification of (**G**) ceramide intensity signal, (**H**) filipin-stained lipid deposits and (**I**) BODIPY-stained lipid deposits (one outlier point in control 2 was removed for plotting) for un-fed and POS-fed controls and patient-derived iPS cell-RPE cells. For BODIPY-stained lipid deposits. Statistical tests used were two-sided, *P = 0.0296 for the interaction between feeding condition and sample (control and patient) in the ANOVA model with the count in fed condition being higher than in unfed condition in patient and lower in control. For Extended Data Fig. 5G-I, the experiment was performed twice with two biological replicates and results are presented as mean ± s.d.

**Extended Data Fig. 6:**
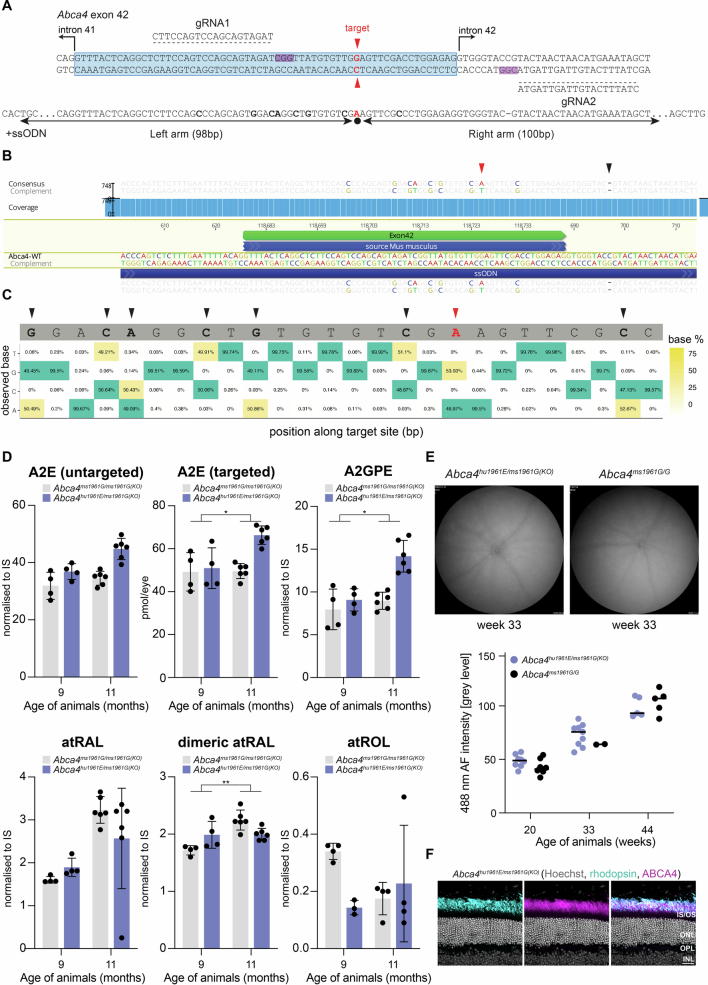
Generation and characterization of *Abca4*^*hu1961E*^ mice. **A**) Strategy for the generation of the *Abca4*^*hu1961E*^ mouse line. *Abca4* exon 42 (blue box) with flanking introns. The PAM sites are highlighted, and the gRNA binding sites are indicated by black dashed lines. The red arrowhead points to the p.Gly1961Glu mutation. Bold nucleotides indicate nucleotide changes due to humanization. Note the deletion in the downstream intron – this is intentional and was introduced to disrupt the PAM site. The deletion is not expected to interfere with splicing as it is at position +9, at which there is no base preference for canonical splicing^52^. **B**) Sequencing of the gDNA of the *Abca4*^*hu1961E*^ allele. The red arrowhead points to the *Abca4* c.5882G>A mutation, the black arrowhead points to a deletion in the intron. **C**) Deep-sequencing of *Abca4*^*hu1961E/ms1961G(KO)*^ mice, where the results indicate heterozygosity. The red arrowhead points to the *Abca4* c.5882G>A mutation and the black arrowheads point to the nucleotide changes due to humanization. **D**) Retinoid and bisretinoid levels measured by LC-HRMS analysis of eyes of *Abca4*^*hu1961E/ms1961G(KO)*^ mice compared to age-matched *Abca4*^*ms1961G/ms1961G(KO)*^ littermates. Retinyl acetate was used as internal standard (IS) for normalization. Absolute levels of A2E (A2E targeted) were determined according to the standard curve for synthetic A2E. Results were obtained from four biological replicates, except for atROL for mutant animals, where 3 biological replicates were used (9 months). Results were obtained from six biological replicates, except for atROL, where 4 biological replicates were used (11 months). Results are presented as mean ± s.d. Statistical tests used were two-sided, A2E targeted: *P = 0.021, A2GPE: *P = 0.016, dimeric atRAL: **P = 0.002, atROL: *P* = 0.056 for the interaction between genotype and age in the ANOVA model. **E**) Fundus autofluorescence images of retinas from an *Abca4*^*hu1961E/ms1961G(KO)*^ and wild-type *Abca4*^*ms1961G/G*^ mouse (top). Quantification of the fluorescent signals at different ages (bottom). For *Abca4*^*hu1961E/ms1961G(KO)*^ animals, results were obtained from eight (week 20), nine (week 33) and five (week 44) biological replicates (eyes). For wild-type *Abca4*^*ms1961G/G*^ animals, results were obtained from eight (week 20), two (week 33) and five (week 44) biological replicates (eyes). **F**) Confocal images of sections from *Abca4*^*hu1961E/ms1961G(KO)*^ mice. Grey: Hoechst; cyan: rhodopsin, magenta: ABCA4 (scale bar: 25 µm).

Importantly, an adjacent adenine (A8) at the third position of the same codon (c.5883A, wobble base) also underwent editing (Extended Data Fig. 2). Independent of whether A7 is edited or not, deamination of A8 does not affect the amino acid sequence. Furthermore, this silent edit can serve as a surrogate assay in models that lack the c.5882G>A mutation (Fig. 1c). The A8 position is not conserved among species and A8 editing is not expected to interfere with splicing^16–18^ (Extended Data Fig. 3a). Throughout the study, we analyzed A7 editing where applicable: in lenti-*ABCA4*^*1961E*^ HEK293T cells, *ABCA4*^*1961E/E*^ human retinal organoids (Extended Data Figs. 4 and 5) and *Abca4*^*hu1961E/ms1961G(KO)*^ mice (Extended Data Fig. 6). We evaluated A8 editing in wild-type models that are most relevant for therapeutic translation (that is, wild-type NHPs, postmortem human retinal explants and RPE/choroid explants) by using the wild-type gRNA (wt-gRNA; Fig. 1c and Supplementary Table 1). We read out editing at the gDNA level to reflect gene correction in all cells and at the *ABCA4* mRNA level to quantify gene correction in *ABCA4*-expressing cells, in photoreceptors, and in RPE cells (Supplementary Fig. 1).

While models carrying the c.5882G>A mutation allowed testing of the base-editor efficiency at the target base, we found no evidence of a Stargardt disease-related phenotype. Specifically, we did not detect lipid-handling defects in patient-derived or in engineered induced pluripotent stem (iPS) cell-derived RPE cells carrying the c.5882A mutation (Extended Data Fig. 5a–i). Nor did we detect any major difference in the levels of bisretinoid or other retinoids in *Abca4*^*hu1961E/ms1961G(KO)*^ mice compared to control mice using mass spectrometry (Extended Data Fig. 6d), which were previously found to be elevated in the *Abca4* knockout mouse model^19^. Furthermore, there was no difference in autofluorescence intensity between *Abca4*^*hu1961E/ms1961G(KO)*^ mice and wild-type mice (Extended Data Fig. 6e), and there was no retinal thinning or disturbance of photoreceptor outer segments (POSs) in *Abca4*^*hu1961E/ms1961G(KO)*^ mice (Extended Data Fig. 6f).

Given the clinical success with an AAV2-based gene therapy^20^, we opted for AAV vectors to deliver ABEs into the eye. As the coding region for ABE exceeds the packaging capacity of AAVs, we designed a split-intein ABE system^10,21^ by separating the ABE within the *Streptococcus pyogenes* Cas9 (SpCas9) into ABE(N) and ABE(C) halves (Fig. 1b). We tested five different split sites in the lenti-*ABCA4*^*1961E*^ HEK293T line using plasmid transfection (Fig. 1d). Base-editing rates with four of the split-intein constructs were comparable to the unsplit editor (Supplementary Table 2a). For further experiments, we selected the ABE variant that is split at position 310 of SpCas9.

ABE8 variants are evolved base editors that have higher activity in primary cells than the original ABE7.10 (ref. ^22^). We compared the activity of 40 different ABE8 variants^22^ to ABE7.10 in the lenti-*ABCA4*^*1961E*^ HEK293T line (Fig. 1e and Supplementary Table 3). A7 and A8 editing rates showed a high correlation (*r* = 0.868), with slightly higher A8 editing. All tested ABE8 variants led to significantly higher editing than ABE7.10 at both positions (Supplementary Table 2b). We retained five variants from this screen and tested them further in human iPS cell-derived RPE (iPS cell-RPE) cells using dual AAV delivery (Fig. 1e). We observed the highest A8 editing with ABE8.5m. With most variants, we observed a low level of c.5880C-to-c.5880T conversion due to ABE-mediated cytosine base editing^23^ (Extended Data Fig. 3b,c). This change is silent and non-conserved and is predicted not to interfere with splicing (Extended Data Fig. 3a). Altogether, we identified an ABE candidate for correction of the c.5882G>A mutation, namely ABE8.5m, split site 310.

**Extended Data Fig. 7:**
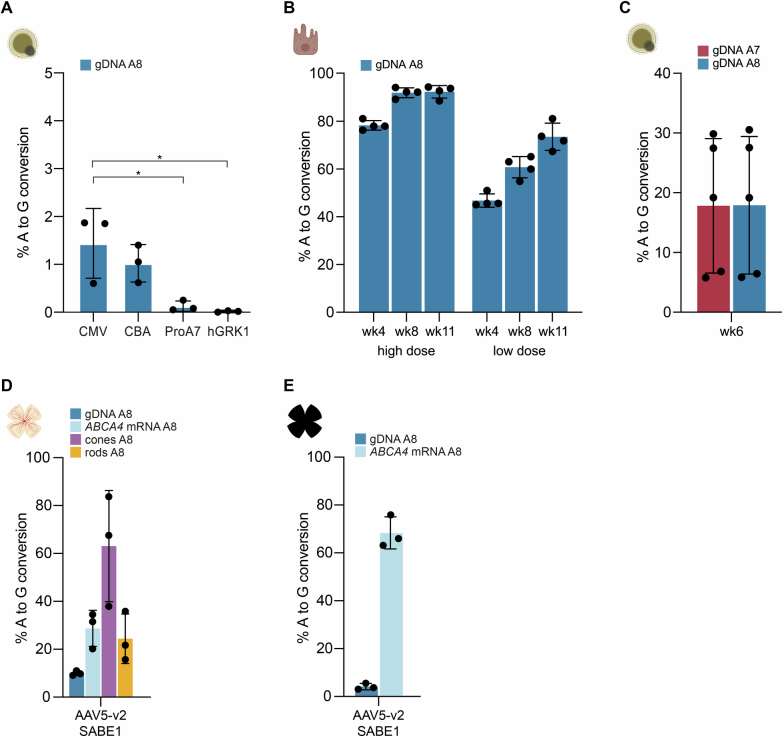
Dual AAV base editing in different in vitro model systems. **A**) Comparison of in vitro base-editing efficiencies at the A8 site in gDNA with different ubiquitous and photoreceptor specific promoters in *ABCA4*^*1961G/G*^ human retinal organoids. Results were obtained from three biological replicates and are presented as mean ± s.d. *P < 0.05 by one-way ANOVA with Tukey’s multiple comparisons test. CMV: cytomegalovirus promoter, CBA: chicken β-actin promoter, ProA7: cone-specific promoter from^43^, hGRK1: human rhodopsin-kinase promoter. **B**) AAV9-PHP.eB-SABE1 editing efficiencies at the A8 site in gDNA of human iPS cell-RPE cells at different time points and two different doses (high dose = 10^6 ^v.g./cell and low dose = 10^5^ v.g./cell). Results were obtained from four biological replicates and are presented as mean ± s.d. **C**) AAV9-PHP.eB-SABE1 editing at the A7 and A8 sites in gDNA of *ABCA4*^*1961E/E*^ human retinal organoids. Results were obtained from five biological replicates and are presented as mean ± s.d. **D**) AAV5-v2-SABE1 editing efficiencies at the A8 site in gDNA, *ABCA4* mRNA and sorted cones and rods of human retinal explants 5 weeks post-transduction. Results were obtained from three biological replicates and are presented as mean ± s.d. **E**) AAV5-v2-SABE1 editing efficiencies at the A8 site in gDNA and *ABCA4* mRNA of human RPE/choroid explants 5 weeks post-transduction. Results were obtained from three biological replicates and are presented as mean ± s.d. Illustrations in **a**–**e** created with BioRender.com.

We packaged ABE8.5m into an AAV9-PHP.eB capsid and evaluated editing in *ABCA4*^*1961E/E*^ retinal organoids, in retinal and RPE/choroid explants (Fig. 1f). To achieve expression of ABEs in cones, rods and RPE cells, we selected the ubiquitous cytomegalovirus (CMV) promoter (Extended Data Fig. 7a), which we combined with the rabbit β-globin polyadenylation signal (rbGlob polyA). While *ABCA4* editing rates in gDNA averaged between 4% and 12% in all models, higher editing rates were found for *ABCA4* mRNA, with averages of 22% and 66% in retinal and RPE/choroid explants, respectively (Fig. 1f).

**Fig. 2:**
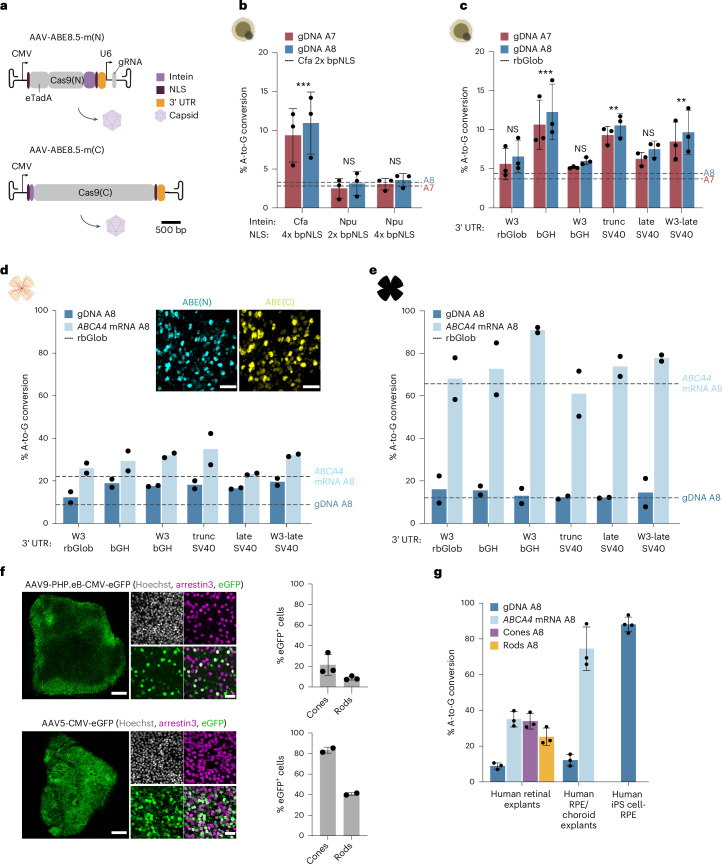
In vitro optimization of the ABE AAV vectors. **a**, Schematic of the dual AAV split-intein ABE8.5m(N) and ABE8.5m(C) vectors. **b**, Base-editing efficiencies in *ABCA4*^*1961E/E*^ human retinal organoids with split-Cfa intein versus split-Npu intein and 2× bpNLS versus 4× bpNLS sequences. Results were obtained from three biological replicates and are presented as the mean ± s.d. ***P = 1.70 × 10^−4^, by three-way mixed-effects ANOVA with Dunnett’s correction, compared to split-Cfa intein and 2× bpNLS. The dashed lines indicate editing rates using a construct with Cfa intein and 2× bpNLS. **c**, Base-editing efficiencies using different 3′ UTRs in *ABCA4*^*1961E/E*^ human retinal organoids. Results were obtained from three biological replicates and presented as the mean ± s.d. **P < 0.01, ***P < 0.001, by three-way mixed-effects ANOVA with Dunnett’s correction, compared to rabbit β-globin polyA (rbGlob). The dashed lines indicate editing rates using a construct with rbGlob polyA. **d**, Base-editing efficiencies using different 3′ UTRs in human retinal explants, and representative immunofluorescence images of ABE(N) and ABE(C) expression in the photoreceptor layer in whole-mount retinas (scale bars, 25 µm). Results were obtained from two biological replicates and are presented as means. The dashed lines indicate editing rates using a construct with rbGlob polyA. Cyan, ABE(N); yellow, ABE(C); gamma correction has been applied to obtain an optimal dynamic range for visualization. **e**, Base-editing efficiencies using different 3′ UTRs in human RPE/choroid explants. Results were obtained from two biological replicates and are presented as means. The dashed lines indicate editing rates using a construct with rbGlob polyA. Illustrations in **b**–**e** and illustrations of the AAV ITRs and AAV capsid in **a** created with BioRender.com. **f**, Representative immunofluorescence images of human retinal explants transduced with the AAV9-PHP.eB or AAV5 capsid encoding CMV-eGFP. Scale bars, 500 µm (left) and 25 µm (middle); right, quantification of eGFP-expressing cones and rods. Results were obtained from two to three biological replicates and are presented as the mean ± s.d. Gray, Hoechst; green, eGFP; magenta, arrestin3. **g**, Base-editing efficiencies with AAV5-SABE1 in human retinal explants and RPE/choroid explants, human iPS cell-RPE cells and sorted cones and rods. Results were obtained from three to four biological replicates and are presented as the mean ± s.d.

Collectively, these results suggest that *ABCA4* base editing can be achieved in target cells of Stargardt disease, but optimization of the vector components is necessary to maximize therapeutic benefit (Fig. 2a).

### In vitro optimization of AAV vector components

We first analyzed the effects of the intein and nuclear localization signal (NLS) on base-editing efficiency in *ABCA4*^*1961E/E*^ retinal organoids (Fig. 2b). A7 editing with split-consensus fast DnaE (Cfa) intein^24^ and two bipartite NLSs^25^ (bpNLSs) per ABE half were threefold higher than with the split-Cfa containing one bpNLS per half. No significant difference in editing rates was detected with split-*Nostoc punctiforme* (Npu) intein^24^ (Supplementary Table 2c). Next, we substituted the rbGlob polyA with different 3′ untranslated region (UTR) elements (Fig. 2c–e). The best candidates in *ABCA4*^*1961E/E*^ retinal organoids and retinal explants were bovine growth hormone (bGH) polyA, truncated simian virus 40 (trunc SV40) polyA and truncated woodchuck hepatitis virus posttranscriptional regulatory element (WPRE) late SV40 (W3-late SV40) polyA, which lead to a twofold to threefold improvement in editing at the gDNA level (Supplementary Table 2d). These candidates also performed well in RPE/choroid explants (Fig. 2e). Immunostaining for ABE(N) and ABE(C) confirmed expression of both halves in human photoreceptors (Fig. 2d). We chose bGH polyA for further studies as it is part of a clinically approved ocular gene therapy vector^20^, and the combination of CMV-ABE8.5m(N)-Cfa-(2x bpNLS)-bGH polyA-U6-gRNA and CMV-Cfa-ABE8.5m(C)-(2x bpNLS)-bGH polyA, denoted as Stargardt adenine base editor 1 (SABE1), became our lead candidate for further studies. We validated SABE1 in iPS cell-RPE cells and *ABCA4*^*1961E/E*^ retinal organoids (Extended Data Fig. 7b,c).

**Extended Data Fig. 8:**
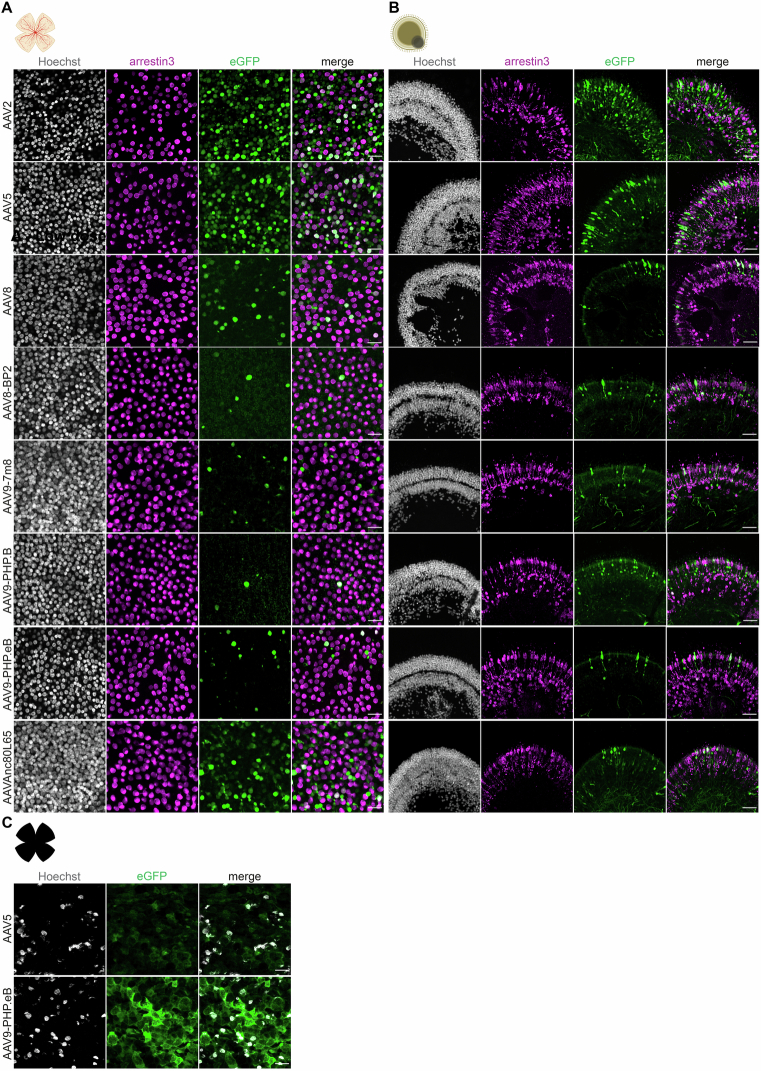
In vitro AAV capsid screen (low dose). **A**) Representative images of *ABCA4*^*1961G/G*^ human retinal explants transduced with different AAV capsids encoding for CMV-eGFP. Results are from 5 weeks after transduction (4.7 × 10^10 ^v.g./explant). Efficient cone-photoreceptor transduction is shown by colocalization of eGFP with arrestin3 (merge) (scale bars: 25 µm). **B**) Representative images of *ABCA4*^*1961G/G*^ human retinal organoids transduced with the same capsids 4 weeks after transduction (3 × 10^10 ^v.g./organoid). Efficient cone-photoreceptor transduction is shown by colocalization of eGFP with arrestin3 (merge) (scale bars: 50 µm). **C**) Representative images of *ABCA4*^*1961G/G*^ human RPE/choroid explants transduced with AAV5- or AAV9-PHP.eB capsids 5 weeks after transduction (4.7 × 10^10 ^v.g./explant) (scale bars: 25 µm). Grey: Hoechst; magenta: arrestin3; green: eGFP. The number of independent experiments and biological replicates are included in the Methods section, under ‘Statistics and Reproducibility’. Illustrations in **a**–**c** created with BioRender.com.

**Extended Data Fig. 9:**
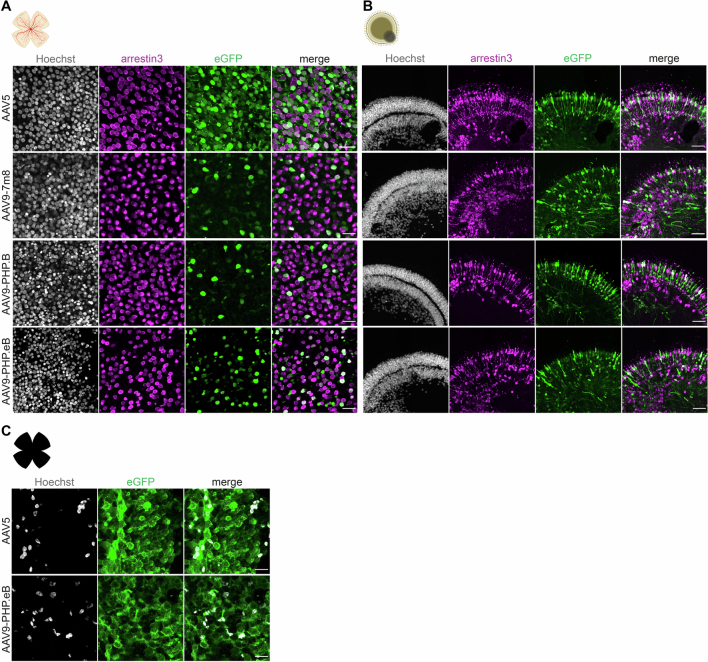
In vitro AAV capsid screen (high dose). **A**) Representative images of *ABCA4*^*1961G/G*^ human retinal explants transduced with different AAV capsids encoding for CMV-eGFP. Results are from 5 weeks after transduction (2.5 × 10^11 ^v.g./explant). Efficient cone-photoreceptor transduction is shown by colocalization of eGFP with arrestin3 (merge) (scale bars: 25 µm). **B**) Representative images of *ABCA4*^*1961G/G*^ human retinal organoids transduced with the same capsids 4 weeks after transduction (1.15 × 10^11 ^v.g./organoid). Efficient cone-photoreceptor transduction is shown by colocalization of eGFP with arrestin3 (merge) (scale bars: 50 µm). **C**) Representative images of *ABCA4*^*1961G/G*^ human RPE/choroid explants transduced with AAV5- or AAV9-PHP.eB capsids 5 weeks after transduction (2.5 × 10^11 ^v.g./explant) (scale bars: 25 µm). Grey: Hoechst; magenta: arrestin3; green: eGFP. The number of independent experiments and biological replicates are included in the Methods, under ‘Statistics and Reproducibility’. Illustrations in **a**–**c** created with BioRender.com.

Next, we screened different AAV capsids to identify serotypes that efficiently transduce photoreceptors and RPE cells. We packaged a CMV-eGFP construct into eight different capsids and transduced human explants and retinal organoids (Extended Data Figs. 8 and 9). Major differences in photoreceptor transduction efficiency were found between different serotypes. AAV5 showed the highest photoreceptor transduction efficiency and outperformed AAV9-PHP.eB (Fig. 2f). We developed a FACS protocol to determine editing rates in cones and rods separately (Supplementary Fig. 2). Editing reached 34% in cones and 25% in rods. High base-editing rates with the AAV5 capsid were also detected in RPE/choroid explants as well as in iPS cell-RPE cells (Fig. 2g).

### In vivo base editing in mice

**Fig. 3:**
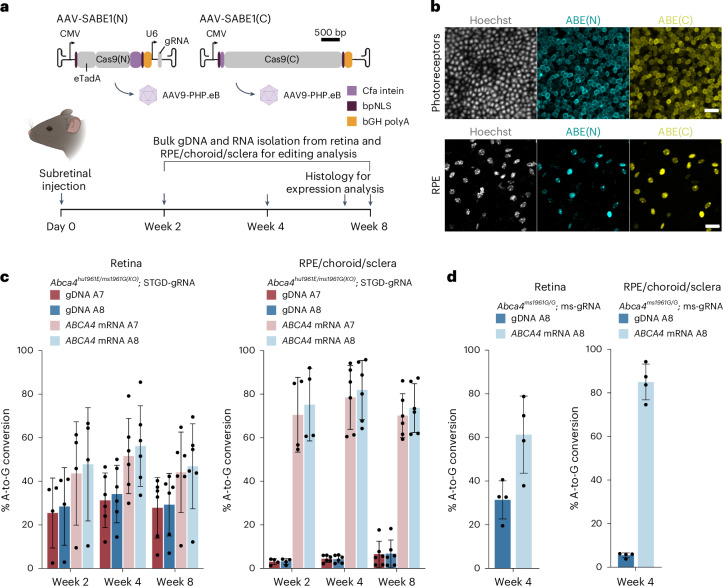
In vivo base editing in mice. **a**, Experimental design. Dual AAV9-PHP.eB-SABE1 was delivered by subretinal injection. Eyes were harvested at 2, 4 or 8 weeks after injection and the retina and RPE/choroid/sclera were processed separately. Illustration in **a** and illustrations of the AAV ITRs and AAV capsid in **a** created with BioRender.com. **b**, Representative immunofluorescence images of ABE(N) and ABE(C) expression in the photoreceptor layer (scale bar, 12.5 µm) and RPE layer (scale bar, 25 µm) of *Abca4*^*ms1961G/G*^ wild-type mice at 7 weeks after injection. Gray, Hoechst; cyan, ABE(N); yellow, ABE(C). **c**, In vivo base-editing efficiencies with the STGD-gRNA targeting the humanized allele in *Abca4*^*hu1961E/ms1961G(KO)*^ mice in the retina and RPE/choroid/sclera at different time points after treatment. The analysis was performed on the humanized allele only. Results were obtained from four (week 2) and six (weeks 4 and 8) biological replicates (eyes) and are presented as the mean ± s.d. **d**, In vivo base-editing efficiencies with the ms-gRNA targeting the mouse alleles in *Abca4*^*ms1961G/G*^ (wt) mice in the retina and RPE/choroid/sclera at 4 weeks after treatment. The analysis was performed on both mouse alleles. Results were obtained from four biological replicates (eyes) and are presented as mean ± s.d. Source data

We tested AAV9-PHP.eB-SABE1 in mice (Fig. 3a and Supplementary Table 4). The majority of individuals with Stargardt disease with the *ABCA4* c.5882G>A mutation are compound heterozygous for the mutation^6^ (Extended Data Fig. 1). Therefore, we utilized our *Abca4* mouse strain (*Abca4*^*hu1961E/ms1961G(KO)*^; Fig. 1c and Extended Data Fig. 6a–c) that carries a humanized *Abca4* c.5882G>A allele (*Abca4*^*hu1961E*^) and an *Abca4* knockout allele (*Abca4*^*ms1961G(KO)*^). Similarly to patients, only a single allele (*Abca4*^*hu1961E*^) can be targeted by the STGD-gRNA. Therefore, the measured editing rates correspond to the percentage of edited cells in this model.

Immunostainings revealed that, after subretinal injection, most photoreceptor and RPE cells expressed both ABE halves simultaneously (79% for retina, 72% for RPE) and a few cells expressed only one half (1% ABE(N), 5% ABE(C) for retina and 1% ABE(N), 4% ABE(C) for RPE; Fig. 3b). We then quantified editing in injected *Abca4*^*hu1961E/ms1961G(KO)*^ mice (Fig. 3c and Supplementary Table 4). At 4 weeks after injection, A7 gDNA editing on the *Abca4*^*hu1961E*^ allele was 31% for the retina and 4% for RPE/choroid/sclera. However, editing rates in the target cells were expected to be higher because mostly photoreceptors (~80% of all cells in the mouse retina^26^) and RPE cells are targeted after subretinal injection. Indeed, at the *Abca4* mRNA level, which represents the target cells, A7 editing was 52% and 79% in the retina and RPE/choroid/sclera, respectively. Longer treatment duration did not increase base-editing rates (Fig. 3c). As previously observed in vitro, A7 editing rates correlated strongly with A8 editing rates, in gDNA and mRNA (Supplementary Table 2e–h).

We also tested editing in *Abca4*^*ms1961G/G*^ wild-type mice (Fig. 3d) to quantify editing in a model that presents two targetable alleles, using the mouse gRNA (Fig. 1c and Supplementary Table 1). At 4 weeks after injection, A8 editing rates were very similar to the rates obtained in the heterozygous *Abca4*^*hu1961E/ms1961G(KO)*^ mice, with 61% and 85% editing on *Abca4* mRNA from retina and RPE/choroid/sclera, respectively (Supplementary Table 2i–l). These data suggest that in models carrying two targetable alleles (*Abca4*^*ms1961G/G*^ mice, wild-type NHPs, human explants), the measured editing percentage corresponds to the percentage of edited cells.

**Extended Data Fig. 10:**
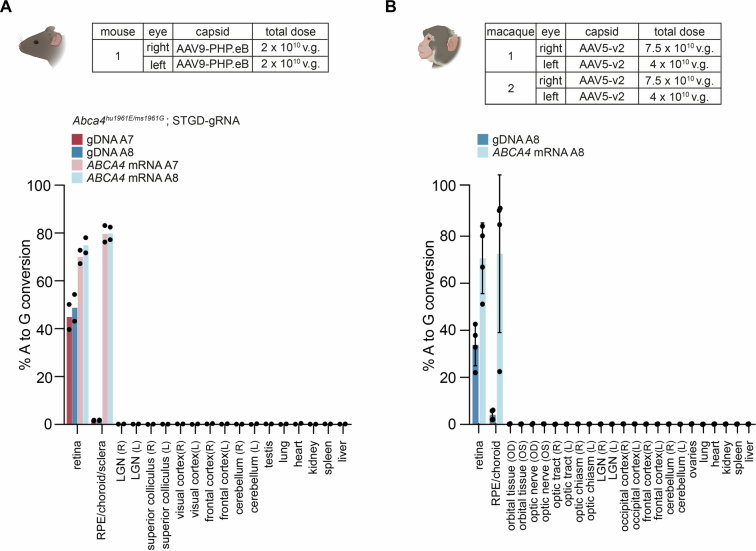
In vivo *ABCA4* base editing in non-ocular tissues from subretinally injected mice and NHPs. **A**) In vivo base-editing efficiencies in mouse tissues at the A7 and A8 site in *Abca4* gDNA and mRNA in the retina and RPE/choroid/sclera as well as in *Abca4* gDNA of different tissues of the visual pathway, cortex, cerebellum and peripheral organs. Tissues were harvested 5 weeks after subretinal injection. Results were obtained from two biological replicates (eyes) and one replicate (non-occular tissues). Results are presented as mean. **B**) In vivo base-editing efficiencies in NHPs at the A8 site in *ABCA4* gDNA and mRNA in the retina and RPE/choroid as well as in *ABCA4* gDNA of different tissues of the visual pathway, cortex, cerebellum and peripheral organs. Tissues were harvested 20 weeks after subretinal injection. Results were obtained from four biological replicates (eyes) and two biological replicates (non-occular tissues). Results are presented as mean ± s.d. Illustrations in **a** and **b** created with BioRender.com.

Finally, we analyzed editing outside the eye in *Abca4*^*hu1961E/ms1961G*^ mice. Despite robust editing in the retina and RPE/choroid/sclera, we have not observed any detectable A7 and A8 editing along the optic pathway, cortex, cerebellum and peripheral organs at the gDNA level (Extended Data Fig. 10a).

### In vivo base editing in NHPs

**Fig. 4:**
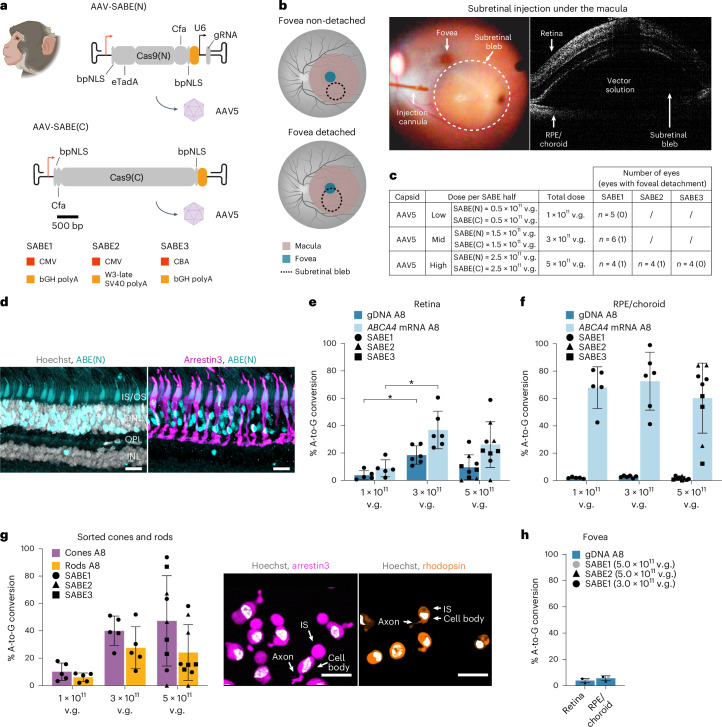
In vivo base editing in NHPs. **a**, Schematic of the SABE(N) and SABE(C) constructs. **b**, Dual AAV5-SABE vectors were delivered to 23 eyes of NHPs by subretinal injection under the macula. Illustration in **a**, illustrations of the AAV ITRs and AAV capsid in **a** and illustration of the eye in **b** created with BioRender.com. Some of the injections also detached the fovea. OCT was used to confirm successful bleb formation. **c**, Experimental design. The number of eyes with fovea detached are indicated in brackets. **d**, Representative immunofluorescence images of ABE(N) expression in a NHP retinal section (scale bars, 20 μm). IS/OS, photoreceptor inner and outer segments; ONL, outer nuclear layer (photoreceptors); OPL, outer plexiform layer; INL, inner nuclear layer. Gray, Hoechst; cyan, ABE(N); magenta, arrestin3; gamma correction has been applied to obtain an optimal dynamic range for visualization. **e**, In vivo base-editing efficiencies in gDNA and *ABCA4* mRNA in the retina with different base-editor constructs and at different doses. Results are presented as the mean ± s.d. Significance for dose response was calculated using a one-way ANOVA with Tukey’s correction, (gDNA: *P = 0.011, *ABCA4* mRNA: *P = 0.011). **f**, In vivo base-editing efficiencies in gDNA and *ABCA4* mRNA in the RPE/choroid with different base-editor constructs and at different doses. Results are presented as the mean ± s.d. **g**, In vivo base-editing efficiencies in gDNA of sorted cones and rods and representative immunofluorescence images of sorted cells (scale bars, 25 μm). IS, photoreceptor inner segment. Results are presented as the mean ± s.d. White, Hoechst; magenta, arrestin3; orange, rhodopsin. **h**, In vivo base-editing efficiencies in the foveal retina and RPE/choroid in gDNA at different dose levels and with different constructs. Results are presented as the mean ± s.d.

Next, we evaluated base editing in NHPs, which are the most relevant animal models for macular diseases given that among the mammalian models, only NHPs have a macula. We chose to deliver AAVs under the macula via subretinal injection, which is a well-established delivery route for therapeutic agents to the retina^20,27,28^. We injected AAV5-SABE into 12 cynomolgus macaques at three different doses (5 × 10^11^, 3 × 10^11 ^and 1 × 10^11^ vector genomes (v.g.); Fig. 4a–c and Supplementary Table 5). We selected vectors based on in vitro results, and we also considered other candidates with possible enhanced in vivo efficacy (Fig. 4a). We injected 15 eyes with AAV5-SABE1 and four eyes with AAV5-SABE2, a second candidate that contains the W3-late SV40 polyA. The W3 element might confer an in vivo expression benefit in combination with the intronless CMV promoter^29^. We also injected four eyes with a chicken β-actin promoter-driven editor, AAV5-SABE3, which is part of an approved gene therapy vector^20^ (Fig. 4c).

To confirm subretinal bleb formation, we used optical coherence tomography (OCT) immediately after surgery (Fig. 4b). Three of 23 eyes were excluded from the study as no subretinal blebs were detected (Supplementary Table 5). Animals were kept for 11 to 27 weeks before proceeding to histology and sequencing of the samples. Immunostaining revealed high expression of ABE(N), mostly localized to photoreceptors (Fig. 4d). Editing in gDNA and *ABCA4* mRNA from the retina was dose dependent, with the highest editing observed at the 3 × 10^11 ^v.g. dose (18% in gDNA, 37% in *ABCA4* mRNA; Fig. 4e and Supplementary Table 2m,n). At the 1 × 10^11 ^v.g. dose, editing fell to 4% and 9% at the gDNA and *ABCA4* mRNA levels, respectively. Editing rates at the RPE/choroid were consistently high at all dose levels tested (Fig. 4f and Supplementary Table 2o,p). Editing rates in the retina and the RPE/choroid with the different AAV5-SABE constructs were similar (Fig. 4e,f and Supplementary Table 2q–t). Editing rates in FACS-sorted cones were significantly higher than in FACS-sorted rods (Fig. 4g and Supplementary Table 2u), with 40% and 28% editing at the 3 × 10^11 ^v.g. dose, respectively.

We also analyzed editing in the fovea of eyes in which the bleb detached the fovea (Fig. 4b,c). Editing was on average 4% and 6% at the gDNA level in the retina and RPE/choroid, respectively (Fig. 4h). We did not assess the editing rates at the *ABCA4* mRNA level, but, based on the relationship between gDNA and mRNA editing in the retina (Supplementary Fig. 3a), we estimate a base-editing percentage of 8%.

Altogether, the results show that the in vitro and mouse results translated to NHPs and that high levels of base correction were reached in NHPs by subretinal injection, but high AAV doses were required.

### Further optimization of AAV-SABE1

**Fig. 5:**
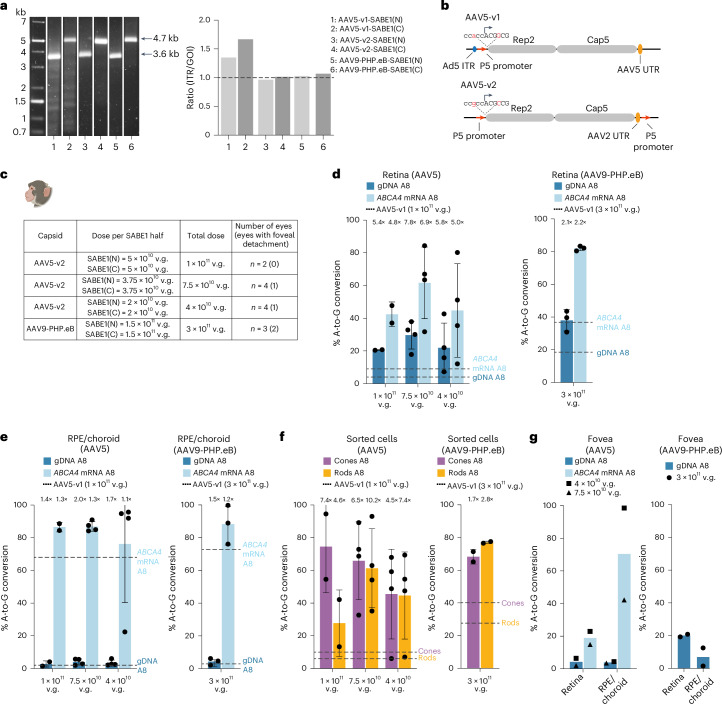
In vivo base editing in NHPs with optimized AAV-SABE1. **a**, Alkaline gel electrophoresis image (left) and 2D-ddPCR (right) of SABE1(N) and SABE1(C) packaged into AAV5-v1 (1–2), AAV5-v2 (3–4) or AAV9-PHP.eB (5–6). GOI, gene of interest. **b**, Schematic showing the key differences between the AAV5-v1 (top) and AAV5-v2 (bottom) Rep/Cap packaging plasmids. **c**, Experimental design showing different injection conditions, the number of eyes injected and the number of eyes in which the fovea was detached (in brackets). Illustration in **c** created with BioRender.com. **d**, In vivo base-editing efficiencies in the retina in gDNA and *ABCA4* mRNA with AAV5-v2-SABE1 (left) and AAV9-PHP.eB-SABE1 (right) at different dose levels. Results are presented as the mean ± s.d. The dashed lines indicate editing rates using AAV5-v1 at 1 × 10^11 ^v.g. per eye (left) or AAV5-v1 at 3 × 10^11 ^v.g. per eye (right); data are from Fig. 4e. Fold improvements compared to AAV5-v1 are indicated above the bars. **e**, In vivo base-editing efficiencies in the RPE/choroid in gDNA and *ABCA4* mRNA with AAV5-v2-SABE1 (left) and AAV9-PHP.eB-SABE1 (right) at different dose levels. Results are presented as the mean ± s.d. The dashed lines indicate editing rates using AAV5-v1 at 1 × 10^11 ^v.g. per eye (left) or AAV5-v1 at 3 × 10^11 ^v.g. per eye (right); data are from Fig. 4f. Fold improvements compared to AAV5-v1 are indicated above the bars. **f**, In vivo base-editing efficiencies in gDNA of sorted cones and rods with AAV5-v2-SABE1 (left) and AAV9-PHP.eB-SABE1 (right) at different dose levels. Results are presented as the mean ± s.d. The dashed lines indicate editing rates using AAV5-v1 at 1 × 10^11 ^v.g. per eye (left) or AAV5-v1 at 3 × 10^11 ^v.g. per eye (right); data are from Fig. 4g. Fold improvements compared to AAV5-v1 are indicated above the bars. **g**, In vivo base-editing efficiencies in the foveal retina and RPE/choroid with AAV5-v2-SABE1 (left) and AAV9-PHP.eB-SABE1 (right) at different dose levels. Results are presented as the mean. Source data

Finally, we further optimized the vector by improving AAV packaging. Previous studies reported heterogeneous genome packaging of AAVs^30,31^ with capsids containing intact genomes, partial genomes and empty capsids. The presence of partial genomes was shown to affect the potency of AAVs^30^. We tested vector genome integrity of AAV5-SABE1 (AAV5-v1-SABE1) using alkaline gel electrophoresis and two-dimensional droplet digital PCR (2D-ddPCR) against the viral inverted terminal repeat (ITR) and the gene of interest (Fig. 5a). We observed multiple shorter bands using alkaline gel electrophoresis and higher ITR titer values compared to gene-of-interest titers using the 2D-ddPCR assay (Fig. 5a). These results indicate the presence of partial AAV genomes in AAV5-v1-SABE1.

We considered two ways to improve packaging. We used an optimized AAV5 Rep/Cap packaging plasmid (AAV5-v2; Fig. 5b), and tested another capsid, AAV9-PHP.eB (Figs. 2 and 3). Indeed for both capsids, we observed improved vector packaging with predominantly intact genomes and similar titer values for the ITR and the gene of interest (Fig. 5a).

Given improved packaging and robust in vitro editing with both capsids (Extended Data Fig. 7b–e), we injected these vectors into NHPs. We used previously applied doses (1 × 10^11 ^v.g. per eye for AAV5-v2-SABE1 and 3 × 10^11 ^v.g. per eye for AAV9-PHPeB-SABE1) and also included lower dose levels for AAV5-v2-SABE1 (7.5 × 10^10 ^v.g. per eye, 4 × 10^10 ^v.g. per eye; Fig. 5c and Supplementary Table 5).

At 1 × 10^11 ^v.g. per eye, editing in the retina with AAV5-v2 capsid was 4.8-fold higher than AAV5-v1-SABE1, with 42% editing on the *ABCA4* mRNA. No significant decrease in editing was detected after reducing the dose by 1.3-fold or 2.5-fold down to 7.5 × 10^10 ^v.g. per eye and 4 × 10^10 ^v.g. per eye, respectively (Supplementary Table 2v,w). Editing rates were 62% and 45% in the retina at the *ABCA4* mRNA level at the two lower doses, respectively (Fig. 5d). At 3 × 10^11 ^v.g. per eye, editing efficiencies in the retina with the AAV9-PHP.eB capsid were 2.2-fold higher than AAV5-v1-SABE1, with 82.0% editing on the *ABCA4* mRNA (Fig. 5d and Supplementary Table 2v,w). In the RPE/choroid *ABCA4* mRNA, editing rates remained high at all dose levels for both capsids (Fig. 5e and Supplementary Table 2x,y).

The highest increase in editing rates with AAV5-v2 was detected in cones. At 1 × 10^11 ^v.g. per eye, we observed a 7.4-fold increase in editing rates with AAV5-v2-SABE1 compared to AAV5-v1-SABE1, with 75% editing (Fig. 5f). These high base-editing rates in cones were maintained after reducing the dose to 7.5 × 10^10 ^v.g. per eye and 4 × 10^10 ^v.g. per eye, with 66% and 46% editing, respectively (Supplementary Table 2z). Editing with AAV9-PHP.eB-SABE1 at 3 × 10^11 ^v.g. per eye in cones was 1.7-fold higher than AAV5-v1-SABE1 and reached 68% (Fig. 5f and Supplementary Table 2z).

We also analyzed editing in the fovea, in eyes in which the bleb detached the fovea (Fig. 5c). With AAV5-v2-SABE1, we observed editing of 4% and 4% at the gDNA level in retina and RPE/choroid, respectively. At the *ABCA4* mRNA level, editing was 19% and 70% in the foveal retina and RPE/choroid, respectively. Editing rates with AAV9-PHP.eB-SABE1 reached on average 20% and 7% at the gDNA level. Based on the relationship between gDNA and mRNA in the retina, this level of editing corresponds to 42% for retina at the *ABCA4* mRNA level (Supplementary Fig. 3a).

Finally, we also analyzed editing outside the retina and RPE/choroid. Despite robust editing in the retina and RPE/choroid, we did not detect editing in the optic nerves, optic pathways, orbital tissue, central nervous system and peripheral organs (Extended Data Fig. 10b).

These results show that the potency of the AAV vectors is dependent on vector genome integrity. AAVs with predominantly intact vector genomes, such as AAV5-v2-SABE1 and AAV9-PHP.eB-SABE1, led to higher editing rates at the same applied dose compared to AAV5-v1-SABE1, which contained more partial vector genomes. Furthermore, these results confirm that increasing vector potency by optimizing the AAV preparation enables reductions in the vector dose.

### Off-target profiling

**Fig. 6:**
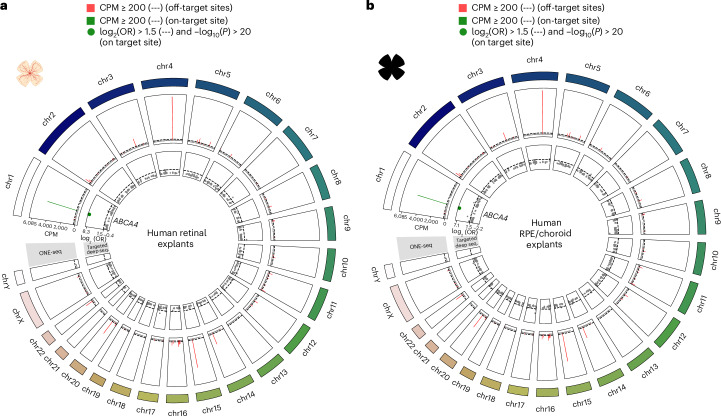
Off-target profile of the STGD-gRNA on human retinal and RPE/choroid explants. **a**,**b**, Circos plots visualizing the off-target profile of the STGD-gRNA. Each sector in the circos plot corresponds to one chromosome that is indicated by the number in the outermost track. The second track shows the results from the ONE-seq assay with the bars representing the counts per million (CPM) for each genomic site. The green bar corresponds to the on-target site, and the red bars represent off-target sites for which the CPM passed the assay threshold of 200 CPM. The innermost track shows the targeted sequencing results of the sites nominated by the ONE-seq assay as well as sites with ≤3 mismatches to the STGD-gRNA, from human retinal explants (**a**; 399 genomic sites; 811 adenine base-editing positions) and RPE/choroid explants (**b**; 401 genomic sites; 812 adenine base-editing positions) treated with AAV5-v2-SABE1. The logarithm of base 2 of the odds ratio (log_2_(OR)) quantifying the A-to-G enrichment at each genomic site in the treated samples is plotted along the vertical axis of the track. Statistical significance was determined by Fisher’s exact test and expressed as −log_10_ *P* value. The on-target site, for which the logarithm of the OR was significant (−log_10_(*P* value) > 20), is colored in green. Sites for which the enrichment was not significant (−log_10_(*P* value) < 20) are colored in gray. Illustrations in **a** and **b** created with BioRender.com.

To analyze genome-wide off-target effects (Fig. 6a,b) of base editing on human tissue, we first performed an in silico identification of candidate sites based on gRNA complementarity. For 10,358 identified sites, we then performed an OligoNucleotide Enrichment and sequencing (ONE-seq^32^) assay using ABE8.5m, STGD-gRNA and EndoV nuclease. The 56 genomic sites nominated by ONE-seq, and sites with fewer or equal to three mismatches to the STGD-gRNA, were further analyzed from PCR-amplified gDNA of treated human retinal and RPE/choroid explants. We did not identify any off-target sites showing a significant A-to-G enrichment, suggesting that c.5882A base editing is precise and does not induce relevant off-target effects.

## Discussion

We have demonstrated highly effective and precise base editing for Stargardt disease in the relevant cell types in mice, NHPs and human tissues. To maximize the chance of successful clinical translation, we established and optimized editing in human models, such as retinal organoids and human explants. We observed a linear relationship between editing rates at the two editable positions and found that target base editing is on average 92% of wobble base editing across all mutation-carrying models (Supplementary Fig. 3b,c).

A key requirement for therapeutic application of base editing is the demonstration of precise base correction in a high percentage of clinically relevant target cells. The evaluation of base-editing rates in *ABCA4* mRNA and from sorted photoreceptors allowed us to specifically determine editing rates in cells expressing the causative gene, including cones, rods and RPE cells.

Although we observed robust editing in the target cells, we could not evaluate the functional effect of base editing, due to the lack of a Stargardt disease-related phenotype in mice and iPS cell-RPE cells. Compared to other *ABCA4* mutations, p.Gly1961Glu has unique phenotypical characteristics: First, the mutation has a predominant effect on foveal cones; disease progression rate outside the fovea is slow^33,34^. Second, lipid deposition in RPE cells is either absent or reduced^33,35^. These characteristics likely explain the lack of a phenotype in our models: we have not observed photoreceptor degeneration or loss of outer segments in the mouse retina, which lacks the fovea and is dominated by rods, and we have not detected alterations of retinal lipids in mice or iPS cell-RPE cells.

The lack of a representative phenotype in *ABCA4* p.Gly1961Glu disease models presents a challenge for therapy development. Our base-editing approach, however, corrects the disease-causing mutation at the genomic level and thereby acts on the root cause of the disease. A beneficial functional effect in the rescued cells is, therefore, to be expected, and will likely be correlated to the percentage of base-edited cells.

Consequently, it is important to determine the minimum percentage of cells that need to be rescued for meaningful vision. Visual acuity is determined by foveal cone photoreceptors, whose proper function depends on the support of the RPE cells. Therefore, we anticipate that gene correction both in cones and in RPE cells is important to preserve foveal vision. In RPE cells, we found efficient editing with all constructs and doses tested. In cones, the editing rate was more sensitive to the dose and vector design. Therefore, we expect that editing in cones will determine patient outcomes.

There are different ways to estimate the minimum number of cones needed for meaningful vision. First, by measuring the stacking density of cone outer segments in the fovea and extrapolating to lower stacking densities, it was found that 12.5% of cones can support vision that is better than legal blindness, that is, visual acuity of 20/200 (‘10% vision’), normal vision being 20/20 (‘100% vision’)^36^. Second, by simulating loss of cones in normal subjects, it was found that only 12.5% of photoreceptors are required to support a gratings visual acuity of 20/60 (ref. ^37^), sufficient for most daily visual tasks. Third, mathematical modeling of the results from a small dot detection task revealed that 8–15% of cones can support a visual acuity of 20/100–20/30 (ref. ^38^), sufficient for most daily visual tasks. Based on these studies, we estimate that a minimum of 12.5% of cones must be rescued to maintain meaningful vision.

The first-generation base-editor vector (AAV5-v1-SABE1) achieved 40% editing in NHP cones at 3 × 10^11 ^v.g. (37% A7 editing predicted). This dose is twofold higher than a clinically approved AAV product^20^. Cone editing at 1 × 10^11 ^v.g. was only 10% (9% A7 editing predicted). Further optimization of AAV5-v1-SABE1 led to a candidate with improved potency, AAV5-v2-SABE1. Subretinal injection of this vector in NHPs resulted in 75% cone editing (69% A7 editing predicted) and 87% editing in RPE cells at the 1 × 10^11 ^v.g. dose level. As a consequence, a 7.5-fold lower dose of AAV5-v2 (4 × 10^10 ^v.g.) was sufficient to achieve editing rates similar to AAV5-v1 at the 3 × 10^11 ^v.g. dose. Key to this result was improvement of the genomic integrity of the AAV preparation, which was impacted by the AAV packaging plasmid.

We have observed efficient editing in the fovea. However, it is difficult to remove foveal tissue without including non-bleb areas, as the fovea is small and close to the bleb margin (Fig. 4b). These untransduced regions might contribute to the decreased editing rates in the foveal samples.

Based on a genome-wide screening, we found no off-target editing in human explants. Furthermore, editing of the *ABCA4* gene was confined to the target organ after subretinal injection.

This study had a primary limitation. The mouse and human models carrying the *ABCA4* p.Gly1961Glu mutation displayed no phenotype; therefore, we were unable to assess whether the observed base-editing efficiency would translate to functional improvements. Furthermore, wild-type NHP models required successful bystander editing as a surrogate marker of editing success.

Altogether, these results suggest that dual AAV-mediated adenine base editing is a promising approach toward a targeted therapy for the most frequent causative mutation in Stargardt disease. These conclusions may be applicable to other inherited retinal diseases where the mutation is targetable by base editing.

## Methods

Note that reference numbers for reagents are found in Supplementary Table 6.

Illustrations of model systems in Figs. 1–6 and Extended Data Figs. 2, 3 and 7–10 and Supplementary Fig. 3 and illustrations of the AAV ITRs and AAV capsid in Figs. 1–4, as well as the illustration of the eye in Fig. 4 were created with BioRender.com.

### Ethics

All human retina and RPE/choroid tissue samples were obtained in accordance with the tenets of the Declaration of Helsinki. Consent was obtained from relatives. Personal identifiers were removed and samples were coded before processing. All experimental protocols regarding human tissues were approved by the local ethics committees (ethical permit numbers: Budapest: Medical Research Council, Ministry of Interior, ETT TUKEB 34851-2/2018/EKU and ETT TUKEB IV/5645-1/2021/EKU; Basel: Ethics Committee of Northwestern and Central Switzerland, EKNZ 2021-01773). Mice were used in accordance with standard ethical guidelines as stated in the European Communities Guidelines on the Care and Use of Laboratory Animals. All animal experiments and procedures were approved by the local ethics committee (permit number: 3048/31896, Kantonales Veterinäramt Basel-Stadt). NHPs were monitored in accordance with the guidelines of the European Directive 2010/63, and handled in strict accordance with good animal practice as defined by the French National Charter on the Ethics of Animal Experimentation. All animal protocols were approved by the French Ministry of Higher Education and Research (permit number: APAFIS 27357-2020092811266511_v2 (28/12/2020)).

### Generation of the HEK293T cells containing a lentivirus-integrated *ABCA4*^*1961E*^

HEK293T (CRL-3216) cells were cultured in DMEM supplemented with 10% (vol/vol) FBS and Glutamax. A lentiviral plasmid encoding the human V5-tagged *ABCA4* gene fragment, comprising the sequence from 72 bp upstream to 123 bp downstream of exon 42 and including the *ABCA4* c.5882G>A mutation, was generated by synthesizing the gene fragment and cloning into the HpaI/ApaI digested pLenti6.4 R4R2 V5-DEST vector. Lentivirus was produced at Charles River Laboratories (Vigene Biosciences) and used to transduce HEK293T cells at a multiplicity of infection of 0.3–10 infectious units per cell. Cells harboring stable lentivirus integrants were selected by 10 µg ml^−1^ blasticidin. The average number of lentiviral integrations per cell was assessed by targeted amplicon sequencing using primers oBTx361 and oBTx362 (Supplementary Tables 7 and 8), which simultaneously amplify the virally integrated *ABCA4* fragment and the endogenous *ABCA4* locus. Cell lines containing an average of two or fewer integrations per cell were used for all base-editing experiments.

### Base editing in HEK293T cells

Base-editor plasmids were codon optimized and synthesized by GeneArt (Thermo Fisher Scientific). We used a split-intein strategy with the base editor split into two halves in the SpCas9 sequence at amino acid positions 310, 313, 456, 469 and 574 (the number refers to the first amino acid on the ABE(C)). The first amino acid on ABE(C) was mutated to cysteine. Lenti-*ABCA4*^*1961E*^ HEK293T cells were seeded in Corning CellBIND 48-well Multiple Well Plates at a density of 35,000 cells. Complementary plasmid pairs containing the split-intein ABE and gRNA, or a full-length base-editor plasmid and gRNA, were combined at a 1:1 molar ratio (1,000 ng total plasmid) for transfection with 1.5 μl Lipofectamine 2000 in a total volume of 25 µl Opti-MEM reduced serum medium. Five days later, 75 µl lysis buffer (10 mM Tris-HCl; pH 8.0) + 0.05% SDS + 100 µg ml^−1^ Proteinase K) was added and the cell lysate was transferred to a 96-well plate. The plate was incubated at 55 °C for 1 h followed by heat inactivation at 95 °C for 20 min.

### AAV vector production and titration

AAV vectors were produced using transient triple transfection of suspension cultures of HEK293T cells with plasmids containing (i) AAV Rep and Cap genes, (ii) the transgene flanked by ITR sequences and (iii) adenovirus helper genes. Cells were lysed 72 h after transfection and unpackaged DNA removed by adding Triton X-100, MgCl_2_ and Turbonuclease at final concentrations of 0.25% (vol/vol), 2 mM and 10 U ml^−1^, respectively. The cell lysate was then filtered through a clarification depth filter, followed by filtration through a 0.2-µm filter. Clarified lysate was loaded onto the affinity chromatography column of an ÄKTA Pure chromatography system. Captured AAV was eluted using an elution buffer at a pH of 2.5–3. The eluate was captured and the pH was promptly neutralized. Full and empty AAV particles were separated by cesium chloride density gradient ultracentrifugation. Bands containing full AAV particles were collected and the titer was determined by qPCR. The full particle samples were then diluted in cesium chloride stock solution to a final target concentration and dialyzed into AAV formulation buffer (10 mM Na_2_HPO_4_, 2 mM KH_2_PO_4_, 2.7 mM KCl, 192 mM NaCl, 0.001% Pluronic F-68; pH 7.4) using 100-kDa cutoff dialysis cassettes. Dialysate was filtered using low-protein binding 0.1-µm syringe filters and then aliquoted and stored at −80 °C. Final AAV titers were determined using ddPCR (Supplementary Table 7). For alkaline gel electrophoresis, AAV vectors were denatured at 95 °C for 10 min in Ficoll loading buffer, supplemented with 50 mM NaOH and 0.3% SDS. Denatured samples were run on a horizontal 1% agarose (1× TAE) gel in running buffer composed of 50 mM NaOH and 1 mM EDTA (40 V, 16.5 h, 4 °C). Gels were then imaged with a ChemiDoc Gel Imager. 2D-ddPCR was performed as previously described^39^ using the QX200 ddPCR system (Bio-Rad) and ddPCR Supermix for Probes (no dUTP; Bio-Rad; Supplementary Table 7).

### Generation of an iPS cell line containing the *ABCA4* c.5882G>A mutation

We used CRISPR-mediated homology-directed repair for knock-in of the *ABCA4* c.5882G>A mutation into the previously published female 01F49i-N-B7 iPS cell line^40^ (Extended Data Fig. 4). An additional silent change (c.5871G>A) was introduced to inactivate the protospacer adjacent motif (PAM) site. A two-part gRNA complex was formed by combining the designed crRNA (Alt-R CRISPR–Cas9 crRNA, IDT; Supplementary Table 7) with the tracrRNA (Alt-R CRISPR–Cas9 tracrRNA, IDT). The gRNA was combined with the Cas9 nuclease protein (Alt-R SpCas9 Nuclease, IDT) to form a CRISPR–Cas9 ribonucleoprotein (RNP) complex. The repair template containing the target mutation and the PAM disruption was designed as a 100-bp-long single-stranded oligonucleotide (ssODN; Supplementary Table 7 and Extended Data Fig. 4). iPS cells were dissociated to single cells and 250,000 cells transfected with 25 μl of the transfection mix containing 20 µl of nucleofection buffer (P3 Primary Cell 4D-NucleofectorTM X Kit S, Lonza), 5 µl of the RNP complex, 1.2 µl of ssODN template and 2.6 µl of PBS. The transfection was performed in a 4D-Nucleofector X Unit using the CA-137 program. For identification of successfully edited clones, individual colonies were picked manually and were expanded in a 96-well plate. gDNA was extracted by adding 40 µl QuickExtract DNA Extraction Solution to each well. We performed targeted PCR by adding 1 μl of cell lysate (1:5 dilution) to a 25-μl PCR reaction containing GoTaq Hot Start Master Mix Green and 0.5 μl of the primers (10 µM, oT01-24 and oT01-25; Supplementary Table 7). To characterize edited iPS cell clones, we performed long-range Sanger sequencing (Supplementary Table 7), targeted deep sequencing (Supplementary Table 7), flow cytometry to analyze pluripotency markers and an aneuploidy test using ddPCR (Extended Data Fig. 4).

### iPS cell-RPE cell culture and AAV transduction

Human iPS cell-RPE cells were differentiated with small modifications according to previous reports^41^. Briefly, embryoid bodies (EBs) were generated from the female iPS(IMR90)-4-DL-01 line^40^ by seeding 9,000 single cells per well in agarose microwell arrays. EBs were cultivated in six-well plates in growth factor-free chemically defined medium containing IMDM, 45% F-12, 450 µM monothioglycerol, 1% penicillin–streptomycin and 10% KnockOut Serum Replacement. Six days after EB induction, 1.5 nM bone morphogenetic protein 4 was added to the medium for another 3 days. At day 18, RPE spheroids were transferred to a 10-cm cell culture dish and kept in suspension in ‘1:1 medium’ containing DMEM:F-12, 1% N2 Supplement, 1% penicillin–streptomycin, 5 µM fibroblast growth factor receptor inhibitor and 3 µM GSK inhibitor (CHIR99021). The medium was exchanged every 3 days until the RPE spheroids were fully pigmented (12–15 days). Thereafter, to obtain a homogeneous monolayer of 2D RPE, RPE spheroids were dissociated into single cells with TrypLE and seeded on Matrigel-coated 96-well plates at a density of 100,000 cells per cm^2^. Cells were transduced with AAV vectors in a 96-well plate at a multiplicity of infection of 1 × 10^5^ or 1 × 10^6^ v.g. per cell for each split-intein ABE half. Four to eleven weeks later, either the samples were fixed or we proceeded with gDNA isolation.

### iPS cell-derived human retinal organoid culture and AAV transduction

Human retinal organoids were derived from the 01F49i-N-B7 iPS cell line. Briefly, EBs were generated from iPS cells in agarose microwell arrays. EBs were cultivated in neural induction medium for the first 16 days and then in ‘3:1 medium’ containing three parts of DMEM and one part of F-12 medium supplemented with 2% B27 without vitamin A, 1% non-essential amino acid solution and 1% penicillin–streptomycin. Retinal structures were detached from Matrigel-coated six-well plates on days 28–32 using the checkerboard scraping method and cultivated in suspension on 90-mm dishes in 10–15 ml 3:1 medium. From day 42, aggregates were cultured in 3:1 medium supplemented with an additional 10% heat-inactivated FBS and 100 µM taurine. At week 10, the culture medium was supplemented with 1 µM retinoic acid. From week 14, organoids were kept in ‘3:1 N2 medium’ in which the B27 supplement in 3:1 medium was replaced by N2 supplement and retinoic acid was reduced to 0.5 µM. Human retinal organoids were transduced with 1.15 × 10^11 ^v.g. per organoid from each intein-split ABE half AAV vector. Four to seven weeks later, either the samples were fixed or we proceeded with bulk gDNA and RNA isolation.

### Human retina and RPE/choroid culture and AAV transductions

Human retinas and RPE/choroid samples were collected at the Department of Ophthalmology, Semmelweis University (Budapest, Hungary) or at the Department of Ophthalmology, Basel University Hospital (Basel, Switzerland). Regarding specification about human donor sex, samples in Figs. 1, 2d,e and 6 were from male donors, and samples in Fig. 2f, Extended Data Figs. 7d,e, 8a,c and 9a,c were from female donors. Briefly, after enucleation, the vitreous was removed and the retina and the RPE/choroid layers were separated. We cultured ~5 × 5 mm tissue pieces on polycarbonate membrane inserts with the photoreceptor side down or the choroid side down. The cultures were maintained at 37 °C in 5% CO_2_ in DMEM–F-12 medium supplemented with 0.1% BSA and 10 μM *O*-acetyl-l-carnitine hydrochloride, 1 mM fumaric acid, 0.5 mM galactose, 1 mM glucose, 0.5 mM glycine, 10 mM HEPES, 0.05 mM mannose, 13 mM sodium bicarbonate, 3 mM taurine, 0.1 mM putrescine dihydrochloride, 0.35 μM retinol, 0.3 μM retinyl acetate, 0.2 μM (+)-α-tocopherol, 0.5 mM ascorbic acid, 0.05 μM sodium selenite, 0.02 μM hydrocortisone, 0.02 μM progesterone, 1 μM insulin, 0.003 μM 3,3′,5-triiodo-l-thyronine, 2,000 U penicillin and 2 mg streptomycin. The medium was changed every second day. AAV vectors containing eGFP or base editors were added 1–2 days after enucleation at 1.66 × 10^11 ^v.g. per explant. Four to seven weeks later, either the samples were fixed or we proceeded with bulk gDNA and RNA isolation.

### Single-cell RNA sequencing

We performed single-cell RNA sequencing of human retinal organoids and human retinal and RPE/choroid explants to analyze expression of *ABCA4*. We loaded 8,000 dissociated cells onto a 10x Genomics Chromium Next GEM Chip G. Single-cell RNA-sequencing libraries were prepared using the Chromium Next GEM Single Cell 3′ Reagent Kits version 3.1 according to the manufacturer’s manual (version CG000204_Rev_C for the version 3.1 kit). Indexed sequencing libraries were sequenced using an Illumina HiSeq 2500 sequencer.

### Mice

All mice were maintained in a pathogen-free environment with ad libitum access to food and drinking water. C57BL/6J wild-type mice were obtained from Charles River Laboratories. *Abca4*^*huG1961E/E*^ mice (B6-*Abca4*^*em1(huEx42-G1961E)Brsk*^, short: *Abca4*^*hu1961E/E*^) were created by the University of Basel, Centre for Transgenic Models (Basel, Switzerland). Briefly, we used the CRISPR-EZ technique^42^ with two gRNAs (Extended Data Fig. 6) to humanize 8 nucleotides in a 34-nucleotide-long region in exon 42 of the *Abca4* gene and to simultaneously introduce the *Abca4* c.5882G>A mutation. One-cell embryos were electroporated with SpCas9/gRNA RNP particles and an ssODN template (Supplementary Table 7). One C nucleotide in the +9 position in intron 42 was deleted to inactivate the PAM site. This nucleotide deletion is not expected to alter splicing and is not conserved between mouse and human. The mouse line was validated using primers that bind outside the targeted region (oT06-37 and oT06-38; Supplementary Table 7). Targeted deep sequencing was used to confirm the sequence identity (Extended Data Fig. 6). To obtain *Abca4*^*hu1961E/ms1961G(KO)*^ heterozygous mice, we crossed *Abca4*^*huG1961E/E*^ animals to B6.129S-Abca4^tm1Ght^/J mice (JAX 026800, Jackson Laboratories).

### Subretinal injection of mice

Subretinal injections were performed at age 11–22 weeks (Supplementary Table 4) as described before^43^. In brief, animals were anesthetized by subcutaneous injection of fentanyl, medetomidine and midazolam (0.05 mg per kg body weight, 0.5 mg per kg body weight and 5 mg per kg body weight, respectively). A small incision was made with a sharp 27-gauge needle at the corneal–scleral divide and a total of 3 × 10^10 ^v.g. per eye of AAV mixture or 1.5 μl of formulation buffer was injected through this incision into the subretinal space using a blunt 5 μl Hamilton syringe held by a micromanipulator. Animals were recovered by subcutaneous injection of naloxone, atipamezole and flumazenil (1.2 mg per kg body weight, 2.5 mg per kg body weight and 0.5 mg per kg body weight, respectively).

### NHPs

Female cynomolgus macaques (*Macaca fascicularis*) were used in the study (Supplementary Table 5). Animals were housed at the Simian Laboratory Europe facility (Silabe).

### Subretinal injections in macaques

NHPs underwent a comprehensive ophthalmological examination by a veterinary ophthalmologist before injection. We obtained preinjection fundus photos and OCT scans of the macula and the optic nerve head. Also, NHPs were genotyped to exclude polymorphisms in the target region via PCR and Sanger sequencing using oT04-46, oT04-47, oT04-48 and oT04-49 (Supplementary Table 7). Three days before injection, treatment of the animals with 0.75 mg per kg body weight intramuscular dexamethasone began and continued for one week. One day before injection, animals received 15 mg per kg body weight intramuscular amoxicillin followed by two further doses 48 h apart. Animals were fasted before the day of the surgery (but access to water was maintained). On the day of the surgery, animals were anesthetized using 10 mg per kg body weight intramuscular ketamine. The pupils were dilated using eyedrops containing 0.5% tropicamide and 10% phenylephrine. Animals received propofol (5–10 mg per kg body weight) followed by intubation. The anesthesia was maintained by isoflurane (1–2.5%). The first six animals were injected through a two-port vitrectomy configuration and a manual injection. Two ports were made on the limbus using 25-gauge trocars, one for the endoillumination port and one for the 41-gauge subretinal microinjection cannula. After gently touching the retina with the subretinal cannula, a slow manual injection was performed with the balanced salt solution (BSS) to induce a pre-bleb, followed by injection of the AAV. The remaining 15 animals were injected through a three-port vitrectomy configuration and the injection was controlled via a foot pedal. In this second cohort, a core vitrectomy was performed, followed by pre-bleb formation by BSS and, finally, injection of the AAV. Shortly after injection, we performed OCT imaging to visualize the subretinal blebs. In 3 of 36 injections, we found no OCT evidence for bleb formation; hence, these eyes were excluded (Supplementary Table 5). Animals received subconjunctival antibiotics immediately after the procedure and tobramycin ointment for 7 days following the procedure.

### Processing of NHP eyes

NHP eyes were removed during terminal anesthesia. The bleb region was punched out using a 4-mm biopsy punch. The retina and the underlying RPE/choroid were separately processed. One half of the retinal tissue was used for dissociation and cell sorting, while the remaining retina and the entire RPE/choroid were subjected to bulk gDNA and RNA isolation. The circular edge of the tissue was fixed with 4% paraformaldehyde (PFA) in PBS and processed for histology. In eyes where the fovea was detached, we analyzed base editing in the fovea separately, by removing it with a 2-mm biopsy punch (Supplementary Table 5). A control, non-treated area was also removed and processed similarly.

### gDNA and RNA extraction from cells and tissues

gDNA from human iPS cell-RPE was isolated by adding 40 µl QuickExtract DNA Extraction Solution to each well. Bulk gDNA and RNA from human retinal organoids were isolated using the AllPrep DNA/RNA Micro Kit or DNeasy Blood & Tissue Kit. Bulk gDNA and RNA from human, mouse and macaque retina or RPE/choroid tissue were isolated using the AllPrep DNA/RNA Mini Kit. Tissue samples were disrupted and homogenized using TissueRuptor II (Qiagen) for 20–30 s at full speed. During RNA purification, DNase digestion was performed on the column membrane using DNase I (Qiagen) for 15 min at room temperature (RT). RNA was eluted in 14–30 µl of RNase-free water. For gDNA, a one-step elution was performed in 50–100 µl of elution buffer. Complementary DNA was synthesized from 20–60 ng of RNA using the ProtoScript II First Strand cDNA Synthesis Kit.

### Organ harvest from mice for base-editing analysis in non-ocular tissues

Brain tissue and peripheral organs were collected from an *Abca4*^*hu1961E/ms1961G*^ mouse 4 weeks after subretinal injection of AAV9-PHP.eB-SABE1. The mouse was first deeply anesthetized with a ketamine–xylazine mixture (120 mg per kg body weight ketamine, 16 mg per kg body weight xylazine; intraperitoneal injection) and then transcardially perfused with an artificial cerebrospinal fluid solution (ACSF, 225 mM sucrose, 3 mM KCl, 1.25 mM NaH_2_PO_4_, 26 mM NaHCO_3_, 10 mM d-(+)-glucose, 2 mM MgSO_4_, 2 mM CaCl_2_). Bilateral samples of the cerebellum, superior colliculus, visual cortex, dorsolateral geniculate nucleus and frontal cortex were collected. 2 × 2 × 2-mm tissue samples of testes, lungs, heart, spleen, kidneys and liver were also collected.

### Organ harvest from NHPs for base-editing analysis in non-ocular tissues

Brain and peripheral organ tissues were collected from macaques treated with AAV5-v2-SABE1. Following eye removal, samples from the intraconal orbital tissue were taken. Next, the skull was opened to take samples from the frontal cortex, cerebellum, occipital cortex, lateral geniculate nucleus, optic tract, optic chiasm and intracranial portion of the optic nerve. Subsequently, we took 2 × 2 × 2-mm tissue samples from the ovaries, lung, heart, kidney, spleen and liver. All samples were taken using sterile instruments to avoid cross contamination.

### FACS of rods and cones

Retinal tissues were dissociated using the Neural Tissue Dissociation Kit (P). Briefly, 990 µl of enzyme mix P (40 µl enzyme P and 950 µl buffer X) was added to a ~5 × 5-mm piece of human retina or a ~4 × 3-mm piece of NHP retina and tissues incubated for 20–30 min at 37 °C while shaking. Next, a further 10 µl of enzyme P was added and tissues incubated for another 10 min at 37 °C. When dissociation was complete, 15 µl of enzyme mix A (5 µl enzyme A and 10 µl buffer Y) was added to the samples and mixtures were incubated for another 10–15 min at 37 °C. The cell suspension was then centrifuged at 300*g* for 5 min at 4 °C and cells resuspended in 100 µl of stain buffer (BD Biosciences, supplemented with 5 mM EDTA). Cells were strained through a 70-µm filter followed by a 40-µm filter and fixed using 100 µl of fixation reagent (Medium A). After 15 min, the cells were washed and resuspended in 300 µl of stain buffer. To stain cones and rods, samples were incubated in 100 µl of Medium B containing primary antibodies for 30 min. Cells were then washed in stain buffer and secondary antibody staining was performed in stain buffer. A FACSAria sorter was used to collect rods and cones into 20 µl of QuickExtract DNA Extraction Solution. The numbers of sorted cells were 200–750 cones and 2,000–6,500 rods for human retinas (Fig. 2g). For macaque retinas, the numbers of collected cells were 20–5,000 cones and 1,800–5,000 rods (Fig. 4e and Supplementary Table 5). Immediately after sorting, proteinase K was added and gDNA extraction performed.

### Target amplicon sequencing DNA

For deep sequencing of the *ABCA4* locus, we performed targeted PCR from gDNA, cDNA or cell lysates. Briefly, 2–10 µl gDNA samples or 5 µl cDNA samples were added to a 50 μl PCR reaction. Cell lysates (2 µl human iPS cell-RPE or 10 µl sorted cells) were added to a 100 µl PCR reaction. The PCR reaction contained Q5 Hot Start HiFi 2X Master Mix and 0.4 μM of each primer (Supplementary Table 7). PCR reactions were carried out at 95 °C for 2 min, 30 cycles of (95 °C for 15 s, 65 °C for 20 s and 72 °C for 20 s) and a final 72 °C extension for 2 min. Following amplification, 2 µl of the crude PCR products was barcoded using 0.5 µM of each Illumina barcoding primer pair and Q5 Hot Start High-Fidelity 2X Master Mix in a total volume of 25 µl. The reactions were carried out as follows: 98 °C for 2 min, 10 cycles of (98 °C for 20 s, 60 °C for 30 s and 72 °C for 30 s) and a final 72 °C extension for 2 min. Equal volumes of barcoded PCR products were then pooled and cleaned up using SPRISelect paramagnetic beads using a 0.6× bead/sample ratio. The amplicon (Supplementary Table 8) was sequenced with an Illumina MiSeq instrument.

### Targeted deep-sequencing analysis

FASTQ files were generated from base-call files created by the MiSeq instrument using Illumina blc2fastq (v2.20.0.422). Adaptors were trimmed using trimmomatic (v0.39) with parameters set up to clip Illumina TruSeq adaptors, exclude reads shorter than 20 bases, trim the remaining 3′ end of reads if the average base quality (Phred score) in a 4-bp sliding window dropped below 15, trim any bases with quality scores of 3 or lower at the end of reads, and trim the 4 randomized bases introduced from the round 1 PCR primers. Trimmed reads were aligned to the reference sequence using Bowtie 2 (v2.35) in end-to-end mode with the ‘--very-sensitive’ flag specified. The SAM files created by Bowtie 2 were converted to BAM files, sorted and indexed using SAMTools (v1.9). BAM files were processed using the bam-readcounts tool (https://github.com/genome/bam-readcount/) to generate plain text files summarizing the number of non-reference bases (substitutions), deletions and insertions at each position in the alignment. The minimum base quality (Phred score) for counting a non-reference base was set to 29 in order to exclude low-confidence base calls. Editing rates for each position in the target site were calculated as the fraction of non-reference bases of a given type (for example, G) to the total number of bases passing the base-quality threshold at a given position in the alignment.

### Tissue preparation for histology

All samples were fixed in 4% PFA in PBS. Human retinal and RPE/choroid explants were fixed for 30 min at RT. Mouse eye cups were fixed for 1 h at RT. Human retinal organoids were fixed for 4 h at 4 °C. The edge of the NHP bleb was fixed overnight at 4 °C. After fixation, samples were washed with PBS and cryoprotected in 30% sucrose overnight at 4 °C.

### Tissue embedding and cryosectioning

Human retinal organoids and NHP retinas were embedded in 7.5% gelatin and 10% sucrose in PBS. Mouse eye cups were embedded in a 1:1 mixture of 30% sucrose and OCT medium. Tissues were cryosectioned into 20–25-µm-thick sections using a MICROM International cryostat and mounted onto Superfrost Plus slides. Sections were dried overnight at RT.

### Immunofluorescence staining of tissue cryosections

Slides were dried for 1 h at RT and then rehydrated in PBS. Samples were blocked in a ‘blocking buffer A’ (PBS supplemented with 10% normal donkey serum, 1% (wt/vol) BSA, 0.5% Triton X-100 in PBS and 0.02% sodium azide) at RT for 1 h. Primary antibodies were diluted in 200 µl of ‘blocking buffer B’ (PBS supplemented with 3% normal donkey serum, 1% BSA, 0.5% Triton X-100 in PBS and 0.02% sodium azide) and samples incubated at RT in a wet chamber overnight. Slides were washed three times in PBS for 15 min each with 0.05% Triton X-100. Secondary antibodies were diluted in 200 µl of ‘blocking buffer B’ and samples incubated for 2 h at RT in the dark. Slides were washed two times in PBS for 10 min each with 0.05% Triton X-100 and once in PBS for 10 min and were coverslipped with ProLong Gold.

### Immunofluorescence staining of tissue cryosections with heat-induced antigen retrieval

Slides were baked at 60 °C for 1 h and postfixed for 15 min on ice with 4% PFA in PBS. Samples were washed shortly in PBS and permeabilized for 15 min with 0.5% Triton X-100 in PBS. The slides were then transferred to a plastic container filled with preheated 1× antigen retrieval buffer (100× Tris-EDTA Buffer; pH 9.0) in water and kept in a steamer for 20 min. After washing in PBS, the samples were stained as described above.

### Immunofluorescence staining of whole-mount human retinas

Whole-mount samples were freeze-thawed three times on dry ice and washed in 500 µl PBS for 15 min in a 24-well plate. Samples were then blocked in 200 µl ‘blocking buffer A’ for 1 h on a shaker at RT in the dark. Primary antibodies were diluted in 200–250 µl of ‘blocking buffer B’ and samples incubated for 3–4 days on a shaker at RT in the dark. They were then washed three times for 10–15 min each in 500 µl PBS and incubated with 200 µl of secondary antibodies diluted in ‘blocking buffer B’ for 1.5 h on a shaker at RT in the dark. After three washes in 500 µl PBS for 10–15 min each, samples were mounted on slides with ProLong Gold.

### Immunofluorescence staining of whole-mount samples with heat-induced antigen retrieval

Human or *Abca4*^*ms1961G/G*^ wild-type mouse retinas were placed into a hybridization chamber and permeabilized inside the chamber for 15 min with 0.5% Triton X-100 in PBS. After washing with PBS, the chamber was filled with 1× antigen retrieval buffer in water (100× Tris-EDTA Buffer; pH 9.0), sealed and transferred to a plastic container that was then placed into a steamer for 20 min. The whole mounts were finally washed in PBS and stained as described above.

### RNAScope

Cultured human retina samples were sectioned by cryosectioning. A Hs-ABCA4 probe was used to label *ABCA4* mRNA molecules, and the reaction was developed using the RNAScope 2.5 HD Reagent Kit according to the manufacturer’s instructions. Secondary staining was carried out with the Opal 690 dye at a 1:5,000 dilution in TSA buffer. Images were captured with an Olympus LSM710 laser-scanning confocal microscope.

### Targeting efficiency of cone and rod photoreceptor cells by AAV capsid

We analyzed six to twelve individual regions of interest per retinal whole mount (*n* = 2 (AAV5) and *n* = 3 (AAV9-PHP.eB) human retinal explants; Fig. 2f). Quantification was performed using the ImageJ Plugin ‘Cell counter’ on two separate *z*-plane images. A cone-rich layer (layer 1) was extracted based on arrestin3 expression and a rod-predominant layer (layer 2) was extracted based on the absence of arrestin3 expression and the presence of a rhodopsin signal. Cone targeting efficiency was quantified from layer 1 by determining the percentage of eGFP-positive cells in the overall arrestin3-positive cone population. Rod targeting efficiency was quantified from layer 2 by determining the percentage of eGFP-positive cells in the overall Hoechst-positive rod population.

### Coexpression efficiency of AAV9-PHP.eB-SABE1(N) and AAV9-PHP.eB-SABE1(C) in mouse photoreceptor and RPE cells

We analyzed six regions of interest per mouse retinal and RPE/choroid/sclera whole mounts (*n* = 1). Quantification was performed with an ImageJ Plugin ‘Cell counter’ in a single *z*-plane image from the outer nuclear layer or the RPE layer. Coexpression efficiency was quantified by determining the percentage of cells coexpressing ABE(N) and ABE(C) in the overall Hoechst-positive population.

### Mass spectrometry analysis

Liquid chromatography high-resolution mass spectrometry was performed as described previously^44^. Briefly, mice were euthanized, and eye cups were removed and snap frozen in liquid nitrogen. A 2:1 methanol–chloroform mixture containing retinyl acetate as internal standard (108 nmol l^−1^) was then added to the samples. Samples were homogenized using a tissue lyser (Qiagen) and centrifuged for 10 min at 20,000*g*. The lipid layers were used for further analysis. The solvent was removed under a gentle stream of nitrogen. The dried samples were solubilized in a isopropanol–acetonitrile–water (50:25:25, vol:vol:vol, 100 µl) solution before liquid chromatography high-resolution mass spectrometry analyses. Targeted metabolites were then separated and detected by the mass spectrometer from their exact mass-to-charge ratios as extensively described previously^44^.

### POS treatment of iPS cell-RPE and lipid staining

Patient-derived iPS cell-RPE cells (laboratory of K.B., National Eye Institute; iPS cell-control 1—female, iPS cell-control 2—male, iPS cell-patient 1—female, iPS cell-patient 2—male) or engineered iPS cells (see above) were cultured and differentiated as described previously^45,46^. All lines used in this study have been previously described^45^. Cells were cultured for 6–8 weeks as described previously^46^. Before performing functional assays, cells with tyrosinase-related protein 1 levels, measured by flow cytometry, higher than 30% on day 25 and 98% on day 40 were used for seeding onto 12-mm Transwell plates. iPS cell-RPE cells were treated for 7 days with bovine POSs (10 POSs per cell) as reported previously^45^. After treatment, cells were fixed and analyzed for lipid, cholesterol and ceramide accumulation, as described previously^47,48^. Briefly, cells were fixed in 4% PFA for 20 min at RT, washed three times in PBS and blocked in immunocytochemistry (ICC) buffer (1× PBS, 1% BSA, 0.25% Tween 20, 0.25% Triton X-100) for 1 h at RT. Primary antibodies were added to the cells followed by incubation overnight at RT. Following the overnight primary antibody incubation, the cells were washed three times with ICC buffer. Secondary antibodies were diluted at a 1:800 ratio in ICC buffer, added to the cells, and incubated in the dark for 1 h at RT. After incubation, cells were washed three times with ICC buffer and mounted on a glass slide, with Fluoromount-G aqueous mounting medium. Lipid deposits were stained using BODIPY 493/503 (10 μM per ml). Cells were fixed, incubated in the BODIPY working solution (10 μM in PBS) overnight at 4 °C and washed three times with PBS before mounting.

### POS treatment of engineered human iPS cell-RPE and lipid staining

Engineered human iPS cell-RPE cells were differentiated as described above. Spheroids were dissociated into single cells and seeded on 6.5-mm Transwell plates at a density of 180,000 cells per cm^2^. Seven weeks after seeding, cells were treated for 10 days with bovine POSs (ten POSs per cell). POS treatment was performed in the dark under red light. After treatment, cells were washed three times with PBS and fixed in 4% PFA for 10 min at RT. After fixation, the Transwell membrane was manually cut out from the insert with a scalpel and stained in a 48-well plate. Membranes were stained as described in ‘Immunofluorescence staining of tissue cryosections’. Primary antibodies against the proteins ZO1 and ceramide were used.

### Off-target analysis

Initial in silico identification of candidate off-target sites was performed by running Cas-OFFinder (v2.4)^49^ on the GRCh38 human reference genome to identify genomic sequences up to seven mismatches to the STGD-gRNA. Oligonucleotides corresponding to 10,358 genomic sites were synthesized and a ONE-seq^32^ assay was performed using endonuclease V and ABE8.5m and the STGD-gRNA. We identified 56 sites that reached the assay threshold (normalized read counts count per million > 200). These 56 sites, 317 genomic sites with a mismatch to target lower or equal than 3 base pairs and 31 control sites were included in a targeted deep-sequencing assay. PCR1 was set up with 5 µl 4× rhAmpSeq Library Mix 1 (IDT), 2 µl 10× rhAmpSeq forward panel, 2 µl 10× rhAmpSeq reverse panel and 11 µl gDNA at 9.1 ng µl^−1^. The PCR reaction was carried out as follows: 95 °C for 10 min, 10 cycles of (95 °C for 15 s, 62 °C for 4 min) and, finally, 99.5 °C for 15 min. PCR products were cleaned up using 1.5× Ampure XP beads: 30 µl of RT Ampure beads was added to each reaction, mixed and incubated for 10 min at RT. Solutions were then exposed to a magnet until clarification of the solution. After removal of the supernatant, beads were washed twice with fresh 80% ethanol and elution performed in 13 µl IDTE buffer. The indexing PCR reaction was set up with 5 µl 4× rhAmpSeq Library Mix 2 (IDT), 4 µl i5/i7 Illumina sequencing indexes at 5 µM each and 11 µl cleaned-up PCR1 product and was carried out as follows: 95 °C for 3 min, 15 cycles of (95 °C for 15 s, 60 °C for 30 s, 72 °C for 30 s) and, finally, 72 °C for 1 min and held at 4 °C. PCR products were cleaned up using 1× Ampure XP beads as described above, using 20 µl RT beads and an elution volume of 22 µl IDTE. Quality control of the libraries was performed using the Qubit and D1000 Tapestation. Equal-nanogram aliquots of each library were then pooled and sequenced using the appropriate Illumina sequencer, aiming to achieve roughly 70,000 reads per amplicon sequenced. Sequencing reads were first preprocessed to trim low-quality base calls. Subsequently, paired-end reads were stitched to create consensus reads with adjusted base-quality scores, and successfully stitched reads were aligned to the human reference genome. Base-call frequencies corresponding to each position in all on-target and candidate off-target sites were calculated from the read alignments. Base-call frequencies for treated and untreated samples from the same donor were compared and an odds ratio quantifying the enrichment of each observed variant in the treated sample was calculated. For off-target analysis in the targeted deep-sequencing analysis, treated and untreated samples were paired for comparative analysis. Two thresholds were used to nominate base-editing target sites (on-target and potential off-target), one for effect size and one for significance. The effect size was expressed as the logarithm of 2 of the odds ratio between the frequency of A-to-G substitutions in treated versus untreated samples (threshold was set as 1.5). The significance was expressed as −log_10_ *P* value from Fisher’s exact test (threshold was set as 20).

### Statistics and reproducibility

All sequencing measurements were taken from distinct samples and were only measured once. For Fig. 4e, results were obtained from five (1 × 10^11 ^v.g. dose level), six (3 × 10^11 ^v.g. dose level) and nine (5 × 10^11 ^v.g. dose level) biological replicates (eyes). For Fig. 4f, results were obtained from five (1 × 10^11 ^v.g. dose level), six (3 × 10^11 ^v.g. dose level) and nine (5 × 10^11 ^v.g. dose level) biological replicates (eyes). For Fig. 4g, results were obtained from five (1 × 10^11 ^v.g. dose level), five (3 × 10^11 ^v.g. dose level) and nine (5 × 10^11 ^v.g. dose level) biological replicates (eyes). For Fig. 4h, results were obtained from one (3 × 10^11 ^v.g. dose level) and two (5 × 10^11 ^v.g. dose level) biological replicates (eyes). For Fig. 5d, results were obtained from two (1 × 10^11 ^v.g. dose level) and four (7.5 × 10^10^ and 4 × 10^10 ^v.g. dose level) biological replicates (eyes) for AAV5-v2-SABE1 and three (3 × 10^11 ^v.g. dose level) biological replicates (eyes) for AAV9-PHP.eB-SABE1. For Fig. 5e, results were obtained from two (1 × 10^11 ^v.g. dose level) and four (7.5 × 10^10^ and 4 × 10^10 ^v.g. dose level) biological replicates (eyes) for AAV5-v2-SABE1 and three (3 × 10^11 ^v.g. dose level) biological replicates (eyes) for AAV9-PHP.eB-SABE1. For Fig. 5f, results were obtained from two (1 × 10^11 ^v.g. dose level) and four (7.5 × 10^10 ^and 4 × 10^10 ^v.g. dose level) biological replicates (eyes) for AAV5-v2-SABE1 and two (3 × 10^11 ^v.g. dose level) biological replicates (eyes) for AAV9-PHP.eB-SABE1. For Fig. 5g, results were obtained from one (7.5 × 10^10^ and 4 × 10^10 ^v.g. dose level) biological replicate (eye) for AAV5-v2-SABE1 and two (3 × 10^11 ^v.g. dose level) biological replicates (eyes) for AAV9-PHP.eB-SABE1.

In Extended Data Fig. 8a, for AAV2 (two biological replicates), AAV8 (three biological replicates), AAV8-BP2 (three biological replicates), AAV97m8 (three biological replicates), AAV9-PHP.B (three biological replicates), AAV9-PHP.eB (three biological replicates) and AAVAnc80L65 (three biological replicates), the experiment was performed once. For AAV5, the experiment was repeated twice with three biological replicates.

In Extended Data Fig. 8b, for AAV2 (three biological replicates), AAV5 (three biological replicates), AAV8 (three biological replicates), AAV9-7m8 (three biological replicates), AAV9-PHP.B (three biological replicates) and AAV9-PHP.eB (three biological replicates), the experiment was performed once. For AAV8-BP2 and AAVAnc80L65, the experiment was repeated twice with three biological replicates.

In Extended Data Fig. 8c, for AAV9-PHP.eB (three biological replicates), the experiment was performed once. For AAV5, the experiment was repeated twice with three biological replicates.

In Extended Data Fig. 9a, for AAV9-7m8 (three biological replicates), AAV9-PHP.B (three biological replicates) and AAV9-PHP.eB (three biological replicates), the experiment was performed once. For AAV5, the experiment was repeated twice with three and two biological replicates, respectively.

In Extended Data Fig. 9b, for AAV9-PHP.eB (three biological replicates), the experiment was performed once. For AAV9-7m8 and AVV9-PHP.B, the experiment was repeated twice with three biological replicates. For AAV5, the experiment was repeated twice with three and two biological replicates, respectively.

In Extended Data Fig. 9c, for AAV9-PHP.eB (three biological replicates), the experiment was performed once. For AAV5, the experiment was repeated twice with three and two biological replicates, respectively.

We used the following statistical tests: Linear (mixed) models including three-way mixed-effect ANOVA and two-way mixed-effects analysis of covariance with time as the continuous covariate, two-way ANOVA or *t*-test (one-way ANOVA). The results were corrected for multiple testing using either Dunnett’s or Tukey’s correction. A random effect was introduced into the models when A7 editing and A8 editing had been applied to the same samples to correct for the correlation of the resulting outcomes. In these cases, the estimates of the correlation between the outcomes of A7 editing and A8 editing on the same samples are reported in Supplementary Table 2. All tests were performed with the statistical software package R (version 4.2.2). All statistical tests used were two sided and the *P* values are stated in Supplementary Table 2.

### Reporting summary

Further information on research design is available in the Nature Portfolio Reporting Summary linked to this article.

## Online content

Any methods, additional references, Nature Portfolio reporting summaries, source data, extended data, supplementary information, acknowledgements, peer review information; details of author contributions and competing interests; and statements of data and code availability are available at 10.1038/s41591-024-03422-8.

## Supplementary information

Supplementary Information Supplementary Information, Extended Data Figs. 1–10 legends, Supplementary Figs 1–3 and Supplementary Data source file

Reporting Summary

Supplementary Tables 1–8 Supplementary Table 1. gRNA sequences. Supplementary Table 2. Details of statistical tests. Supplementary Table 3. Testing different ABE versions on HEK293T cells carrying the p.Gly1961Glu mutation. Supplementary Table 4. Detailed mouse study results. Supplementary Table 5. Detailed NHP study results. Supplementary Table 6. List of reagents and antibodies, and biological samples. Supplementary Table 7. Sequences of primers. Supplementary Table 8. Sequences of amplicons.

## Source data

Source Data Fig. 3c Base-editing results in mice from Fig. 3c segregated by sex.

Source Data Fig. 5a Unprocessed alkaline gel electrophoresis image.

## Data Availability

All data are available in the manuscript or the Supplementary Information. AAV vector sequences are included in the Supplementary Information. The sequencing data generated in this study have been deposited in the Sequence Read Archive under accession PRJNA1170171. Source data are provided with this paper.
