# Supplementary material for: High-efficiency base editing in the retina in primates and human tissues
Source: Nat Med. 2025 Jan 8;31(2):490–501. doi: 10.1038/s41591-024-03422-8 (PMC11835749; doi:10.1038/s41591-024-03422-8)
Supplement: Supplementary file 1 — Supplementary Information, Extended Data Figs. 1–10 legends, Supplementary Figs 1–3 and Supplementary Data source file [file 41591_2024_3422_MOESM1_ESM.pdf]

nature medicine

Supplementary information

https://doi.org/10.1038/s41591-024-03422-8

High-efficiency base editing in the retina in primates and human tissues

In the format provided by the authors and unedited

Supplementary Information

SABE1(N) vector sequence

CMV promoter, bpNLS, TadA8.5-m, Cas9(N), Cfa intein(N), bGH polyA, U6 promoter, gRNA

acattgattattgactagttattaatagtaatcaattacggggtcattagttcatagcccatatatgg
agttccgcgttacataacttacggtaaatggcccgcctggctgaccgcccaacgacccccgcccattg
acgtcaataatgacgtatgttcccatagtaacgccaatagggactttccattgacgtcaatgggtgga
gtatttacggtaaactgcccacttggcagtacatcaagtgtatcatatgccaagtacgccccctattg
acgtcaatgacggtaaatggcccgcctggcattatgcccagtacatgaccttatgggactttcctact
tggcagtacatctacgtattagtcatcgctattaccatggtgatgcggttttggcagtacatcaatgg
gcgtggatagcggtttgactcacggggatttccaagtctccaccccattgacgtcaatgggagtttgt
tttggcaccaaaatcaacgggactttccaaaatgtcgtaacaactccgccccattgacgcaaatgggc
ggtaggcgtgtacggtgggaggtctatataagcagagctggtttagtgaaccgtcagatccgctagcg
ccaccATGGAAGGCGCCGATAAGCGGACCGCCGACGGCAGCGAGTTCGAGAGCCCTAAGAAAAAGAGG
AAGGTGAGCGAGGTCGAGTTCTCTCACGAATATTGGATGAGACACGCTCTCACCCTGGCTAAGAGAGC
CAGGGACGAAAGAGAGGTGCCAGTTGGCGCTGTCCTGGTGTTGAACAATCGCGTCATCGGAGAAGGAT
GGAATCGCGCCATTGGCCTGCACGATCCAACCGCACATGCCGAAATTATGGCTCTGCGGCAAGGCGGC
CTCGTGATGCAAAATTACAGACTGATCGATGCTACCCTCTACAGCACCTTCGAGCCCTGTGTCATGTG
TGCTGGGGCAATGATTCACTCCCGGATTGGCCGCGTGGTGTTTGGAGTGCGGAATGCCAAGACTGGCG
CCGCTGGATCTCTGATGGACGTCCTGCACTATCCTGGGATGAACCACCGGGTCGAGATCACAGAGGGA
ATTCTGGCTGACGAGTGCGCTGCCCTGCTGTGCTACTTCTTTAGAATGCCCAGACAGGTGTTCAACGC
CCAGAAAAAAGCTCAGAGCAGCACCGATTCCGGCGGAAGCAGCGGAGGATCTTCTGGAAGCGAAACCC
CAGGCACCAGCGAGTCTGCCACACCAGAATCATCTGGCGGTAGCTCCGGCGGCAGCGACAAGAAGTAT
TCTATCGGACTGGCCATCGGCACCAACTCTGTTGGATGGGCCGTGATCACCGACGAGTACAAGGTGCC
CAGCAAGAAATTCAAGGTGCTGGGCAACACCGACAGGCACAGCATCAAGAAGAACCTGATCGGCGCAC
TGCTGTTCGACTCTGGCGAAACAGCCGAGGCCACCAGACTGAAGAGAACAGCCCGCAGACGGTACACC
AGAAGAAAGAACCGGATCTGCTACCTCCAAGAGATCTTCAGCAACGAGATGGCCAAGGTGGACGACAG
CTTCTTCCACAGACTGGAAGAGTCCTTCCTGGTGGAAGAGGACAAGAAGCACGAGAGACACCCCATCT
TCGGCAACATCGTGGACGAGGTGGCCTACCACGAGAAGTACCCCACCATCTACCACCTGAGAAAGAAA
CTGGTGGACAGCACCGACAAGGCCGACCTGAGACTGATCTATCTGGCCCTGGCTCACATGATCAAGTT
CCGGGGCCACTTCCTGATCGAGGGCGACCTGAATCCTGACAACAGCGACGTGGACAAGCTGTTCATCC
AGCTGGTGCAGACCTACAACCAGCTGTTCGAGGAAAACCCCATCAACGCCAGCGGAGTGGATGCCAAG
GCCATCCTGTCTGCCAGACTGAGCAAGAGCAGACGGCTGGAAAATCTGATCGCCCAGCTGCCTGGCGA
GAAGAAGAATGGCCTGTTCGGCAACCTGATTGCCCTGAGCCTGGGCCTGACACCTAACTTCAAGAGCA
ACTTCGACCTGGCCGAGGACGCCAAACTGCAGCTGAGCAAGGACACCTACGACGACGACCTGGACAAT
CTGCTGGCCCAGATCGGCGATCAGTACGCCGACTTGTTTCTGGCCGCCAAGAATCTGAGCGACGCCAT
CCTGCTGTCCGACATCCTGAGAGTGAACTGCCTGAGCTACGATACCGAGATCCTGACCGTGGAATACG
GCTTCCTGCCTATCGGCAAGATCGTCGAGGAACGGATCGAGTGCACAGTGTACACCGTGGATAAGAAT
GGCTTCGTGTACACCCAGCCTATCGCTCAGTGGCACAACAGAGGCGAGCAAGAGGTGTTCGAGTACTG
CCTGGAAGATGGCAGCATCATCCGGGCCACCAAGGACCACAAGTTTATGACCACCGACGGCCAGATGC
TGCCCATCGACGAGATCTTTGAGAGAGGCCTGGACCTGAAACAGGTGGACGGACTGCCTAGCGGCGGA
TCTGAAGGCGCTGATAAAAGAACCGCCGATGGCTCCGAGTTCGAGAGCCCTAAGAAAAAGCGGAAGGT
GTGAAAGCTTCGACTGTGCCTTCTAGTTGCCAGCCATCTGTTGTTTGCCCCTCCCCCGTGCCTTCCTT
GACCCTGGAAGGTGCCACTCCCACTGTCCTTTCCTAATAAAATGAGGAAATTGCATCGCATTGTCTGA
GTAGGTGTCATTCTATTCTGGGGGGTGGGGTGGGGCAGGACAGCAAGGGGGAGGATTGGGAAGACAAT
AGCAGGCATGCTGGGGACCTGCAGGtgtacaaaaaagcaggctttaaaggaaccaattcagtcgactg
gatccggtaccaaggtcgggcaggaagagggcctatttcccatgattccttcatatttgcatatacga
tacaaggctgttagagagataattagaattaatttgactgtaaacacaaagatattagtacaaaatac
gtgacgtagaaagtaataatttcttgggtagtttgcagttttaaaattatgttttaaaatggactatc
atatgcttaccgtaacttgaaagtatttcgatttcttggctttatatatcttgtggaaaggacgaaac
accGTGTCGAAGTTCGCCCTGGAGGTTTTAGAGCTAGAAATAGCAAGTTAAAATAAGGCTAGTCCGTT
ATCAACTTGAAAAAGTGGCACCGAGTCGGTGCTTTTTT

SABE1(C) vector sequence

CMV promoter, bpNLS, Cfa intein(C), Cas9(C), bGH polyA

acattgattattgactagttattaatagtaatcaattacggggtcattagttcatagcccatatatgg
agttccgcgttacataacttacggtaaatggcccgcctggctgaccgcccaacgacccccgcccattg
acgtcaataatgacgtatgttcccatagtaacgccaatagggactttccattgacgtcaatgggtgga
gtatttacggtaaactgcccacttggcagtacatcaagtgtatcatatgccaagtacgccccctattg
acgtcaatgacggtaaatggcccgcctggcattatgcccagtacatgaccttatgggactttcctact
tggcagtacatctacgtattagtcatcgctattaccatggtgatgcggttttggcagtacatcaatgg
gcgtggatagcggtttgactcacggggatttccaagtctccaccccattgacgtcaatgggagtttgt
tttggcaccaaaatcaacgggactttccaaaatgtcgtaacaactccgccccattgacgcaaatgggc
ggtaggcgtgtacggtgggaggtctatataagcagagctggtttagtgaaccgtcagatccgctagcg
ccaccATGGAAGGAGCTGATAAGCGGACAGCCGACGGCAGCGAGTTCGAGAGCCCTAAGAAGAAAAGA
AAGGTGTCCGGCGGATCTGTCAAGATCATCAGCAGAAAGAGCCTGGGCACCCAGAACGTGTACGATAT
CGGAGTGGGCGAGCCCCACAACTTTCTGCTCAAGAATGGCCTGGTGGCCAGCAACTGCGAGATCACCA
AGGCACCTCTGAGCGCCTCTATGATCAAGAGATACGACGAGCACCACCAGGATCTGACCCTGCTGAAG
GCCCTCGTTAGACAGCAGCTGCCAGAGAAGTACAAAGAGATTTTCTTCGACCAGAGCAAGAACGGCTA
CGCCGGCTACATTGATGGCGGAGCCAGCCAAGAGGAATTCTACAAGTTCATCAAGCCCATCCTCGAGA
AGATGGACGGCACCGAGGAACTGCTGGTCAAGCTGAACAGAGAGGACCTGCTGAGAAAGCAGAGAACC
TTCGACAACGGCAGCATCCCTCACCAGATCCACCTGGGAGAACTGCACGCCATTCTGCGGAGACAAGA
GGACTTTTACCCATTCCTGAAGGACAACCGGGAAAAGATCGAGAAAATCCTGACCTTCAGGATCCCCT
ACTACGTGGGACCACTGGCCAGAGGCAATAGCAGATTCGCCTGGATGACCAGAAAGAGCGAGGAAACC
ATCACTCCCTGGAACTTCGAGGAAGTGGTGGACAAGGGCGCCAGCGCTCAGTCCTTCATCGAGCGGAT
GACCAACTTCGATAAGAACCTGCCTAACGAGAAGGTGCTGCCCAAGCACAGCCTGCTGTACGAGTACT
TCACCGTGTACAACGAGCTGACCAAAGTGAAATACGTGACCGAGGGAATGAGAAAGCCCGCCTTTCTG
AGCGGCGAGCAGAAAAAGGCCATCGTGGATCTGCTGTTCAAGACCAACCGGAAAGTGACCGTGAAGCA
GCTGAAAGAGGACTACTTCAAGAAAATCGAGTGCTTCGACAGCGTCGAGATCTCCGGCGTGGAAGATC
GGTTCAATGCCAGCCTGGGCACATACCACGATCTGCTGAAAATTATCAAGGACAAGGACTTCCTGGAC
AACGAAGAGAACGAGGACATCCTTGAGGACATCGTGCTGACACTGACCCTGTTTGAGGACAGAGAGAT
GATCGAGGAACGGCTGAAAACATACGCCCACCTGTTCGACGACAAAGTGATGAAGCAACTGAAGCGGC
GGAGATACACCGGCTGGGGCAGACTGTCTCGGAAGCTGATCAACGGCATCCGGGATAAGCAGTCCGGC
AAGACCATCCTGGACTTTCTGAAGTCCGACGGCTTCGCCAACAGAAACTTCATGCAGCTGATTCACGA
CGACAGCCTCACCTTCAAAGAGGATATCCAGAAAGCCCAGGTGTCCGGCCAGGGCGATTCTCTGCATG
AGCACATTGCCAACCTGGCCGGCTCTCCCGCCATTAAGAAAGGCATCCTGCAGACAGTGAAGGTGGTG
GACGAGCTTGTGAAAGTGATGGGCAGACACAAGCCCGAGAACATCGTGATCGAAATGGCCAGAGAGAA
CCAGACCACACAGAAGGGACAGAAGAACAGCCGCGAGAGAATGAAGCGGATCGAAGAGGGCATCAAAG
AGCTGGGCAGCCAGATCCTGAAAGAACACCCCGTGGAAAACACCCAGCTGCAGAACGAGAAGCTGTAC
CTGTACTACCTGCAGAATGGACGGGATATGTACGTGGACCAAGAGCTGGACATCAACAGACTGTCCGA
CTACGATGTGGACCATATCGTGCCCCAGTCTTTTCTGAAGGACGACTCCATCGACAACAAGGTCCTGA
CCAGATCCGACAAGAATCGGGGCAAGAGCGACAACGTGCCCTCCGAAGAGGTGGTCAAGAAGATGAAG
AACTACTGGCGACAGCTGCTGAACGCCAAGCTGATTACCCAGCGGAAGTTCGACAATCTGACCAAGGC
CGAAAGAGGCGGCCTGAGCGAACTGGATAAGGCCGGCTTCATCAAGAGACAGCTGGTGGAAACCCGGC
AGATCACAAAGCACGTGGCACAGATTCTGGACTCTCGGATGAACACTAAGTACGACGAGAACGACAAA
CTGATCCGCGAAGTGAAAGTCATCACCCTGAAGTCCAAGCTGGTGTCCGATTTCCGGAAGGATTTCCA
GTTCTACAAAGTGCGCGAGATCAACAACTACCATCACGCCCACGACGCCTACCTGAATGCCGTTGTTG
GAACAGCCCTGATCAAAAAGTACCCTAAGCTGGAAAGCGAGTTCGTGTACGGCGACTACAAGGTGTAC
GACGTGCGGAAGATGATCGCCAAGAGCGAGCAAGAGATTGGCAAGGCAACCGCCAAGTACTTCTTCTA
CAGCAACATCATGAACTTTTTCAAGACAGAGATCACCCTCGCCAACGGCGAGATCAGAAAGCGGCCTC
TGATCGAGACAAACGGCGAAACCGGCGAGATTGTGTGGGATAAGGGCAGAGACTTTGCCACAGTGCGG
AAAGTGCTGAGCATGCCCCAAGTGAATATCGTGAAGAAAACCGAGGTGCAGACAGGCGGCTTCAGCAA
AGAGTCTATCCTGCCTAAGCGGAACTCCGACAAGCTGATCGCCAGAAAGAAGGACTGGGACCCCAAGA
AGTACGGCGGCTTCGATTCTCCTACCGTGGCCTATAGCGTGCTGGTGGTGGCCAAAGTGGAAAAGGGC
AAGTCCAAGAAACTCAAGAGCGTGAAAGAGCTGCTGGGGATCACCATCATGGAAAGAAGCAGCTTCGA
GAAGAATCCGATCGATTTCCTCGAGGCCAAGGGCTACAAAGAAGTGAAAAAGGACCTGATCATCAAGC
TCCCCAAGTACTCCCTGTTCGAGCTGGAAAACGGCCGGAAGAGAATGCTGGCCTCTGCTGGCGAACTG

CAGAAGGGAAACGAACTGGCCCTGCCTAGCAAATATGTGAACTTCCTGTACCTGGCCAGCCACTATGA
GAAGCTGAAGGGCAGCCCCGAGGACAATGAGCAAAAGCAGCTGTTTGTGGAACAGCACAAGCACTACC
TGGACGAGATCATCGAGCAGATCAGCGAGTTTAGCAAGAGAGTGATTCTGGCCGACGCCAATCTGGAC
AAAGTGCTGTCCGCCTACAACAAGCACCGGGACAAGCCTATCAGAGAGCAGGCCGAGAATATCATCCA
CCTGTTTACCCTGACCAACCTGGGAGCCCCTGCCGCCTTCAAGTACTTTGACACCACCATCGACCGGA
AGCGGTACACCTCCACCAAAGAGGTGCTGGACGCCACTCTGATCCACCAGTCTATCACCGGCCTGTAC
GAGACACGGATCGACCTGTCTCAACTCGGAGGCGACGAAGGCGCCGATAAGAGAACCGCCGATGGCTC
TGAGTTCGAGAGCCCCAAGAAAAAGCGCAAAGTGTGAAAGCTTCGACTGTGCCTTCTAGTTGCCAGCC
ATCTGTTGTTTGCCCCTCCCCCGTGCCTTCCTTGACCCTGGAAGGTGCCACTCCCACTGTCCTTTCCT
AATAAAATGAGGAAATTGCATCGCATTGTCTGAGTAGGTGTCATTCTATTCTGGGGGGTGGGGTGGGG
CAGGACAGCAAGGGGGAGGATTGGGAAGACAATAGCAGGCATGCTGGGGA

SABE2(N) vector sequence

CMV promoter, bpNLS, TadA8.5-m, Cas9(N), Cfa intein(N), W3-late SV40 polyA, U6 promoter, gRNA

acattgattattgactagttattaatagtaatcaattacggggtcattagttcatagcccatatatgg
agttccgcgttacataacttacggtaaatggcccgcctggctgaccgcccaacgacccccgcccattg
acgtcaataatgacgtatgttcccatagtaacgccaatagggactttccattgacgtcaatgggtgga
gtatttacggtaaactgcccacttggcagtacatcaagtgtatcatatgccaagtacgccccctattg
acgtcaatgacggtaaatggcccgcctggcattatgcccagtacatgaccttatgggactttcctact
tggcagtacatctacgtattagtcatcgctattaccatggtgatgcggttttggcagtacatcaatgg
gcgtggatagcggtttgactcacggggatttccaagtctccaccccattgacgtcaatgggagtttgt
tttggcaccaaaatcaacgggactttccaaaatgtcgtaacaactccgccccattgacgcaaatgggc
ggtaggcgtgtacggtgggaggtctatataagcagagctggtttagtgaaccgtcagatccgctagcg
ccaccATGGAAGGCGCCGATAAGCGGACCGCCGACGGCAGCGAGTTCGAGAGCCCTAAGAAAAAGAGG
AAGGTGAGCGAGGTCGAGTTCTCTCACGAATATTGGATGAGACACGCTCTCACCCTGGCTAAGAGAGC
CAGGGACGAAAGAGAGGTGCCAGTTGGCGCTGTCCTGGTGTTGAACAATCGCGTCATCGGAGAAGGAT
GGAATCGCGCCATTGGCCTGCACGATCCAACCGCACATGCCGAAATTATGGCTCTGCGGCAAGGCGGC
CTCGTGATGCAAAATTACAGACTGATCGATGCTACCCTCTACAGCACCTTCGAGCCCTGTGTCATGTG
TGCTGGGGCAATGATTCACTCCCGGATTGGCCGCGTGGTGTTTGGAGTGCGGAATGCCAAGACTGGCG
CCGCTGGATCTCTGATGGACGTCCTGCACTATCCTGGGATGAACCACCGGGTCGAGATCACAGAGGGA
ATTCTGGCTGACGAGTGCGCTGCCCTGCTGTGCTACTTCTTTAGAATGCCCAGACAGGTGTTCAACGC
CCAGAAAAAAGCTCAGAGCAGCACCGATTCCGGCGGAAGCAGCGGAGGATCTTCTGGAAGCGAAACCC
CAGGCACCAGCGAGTCTGCCACACCAGAATCATCTGGCGGTAGCTCCGGCGGCAGCGACAAGAAGTAT
TCTATCGGACTGGCCATCGGCACCAACTCTGTTGGATGGGCCGTGATCACCGACGAGTACAAGGTGCC
CAGCAAGAAATTCAAGGTGCTGGGCAACACCGACAGGCACAGCATCAAGAAGAACCTGATCGGCGCAC
TGCTGTTCGACTCTGGCGAAACAGCCGAGGCCACCAGACTGAAGAGAACAGCCCGCAGACGGTACACC
AGAAGAAAGAACCGGATCTGCTACCTCCAAGAGATCTTCAGCAACGAGATGGCCAAGGTGGACGACAG
CTTCTTCCACAGACTGGAAGAGTCCTTCCTGGTGGAAGAGGACAAGAAGCACGAGAGACACCCCATCT
TCGGCAACATCGTGGACGAGGTGGCCTACCACGAGAAGTACCCCACCATCTACCACCTGAGAAAGAAA
CTGGTGGACAGCACCGACAAGGCCGACCTGAGACTGATCTATCTGGCCCTGGCTCACATGATCAAGTT
CCGGGGCCACTTCCTGATCGAGGGCGACCTGAATCCTGACAACAGCGACGTGGACAAGCTGTTCATCC
AGCTGGTGCAGACCTACAACCAGCTGTTCGAGGAAAACCCCATCAACGCCAGCGGAGTGGATGCCAAG
GCCATCCTGTCTGCCAGACTGAGCAAGAGCAGACGGCTGGAAAATCTGATCGCCCAGCTGCCTGGCGA
GAAGAAGAATGGCCTGTTCGGCAACCTGATTGCCCTGAGCCTGGGCCTGACACCTAACTTCAAGAGCA
ACTTCGACCTGGCCGAGGACGCCAAACTGCAGCTGAGCAAGGACACCTACGACGACGACCTGGACAAT
CTGCTGGCCCAGATCGGCGATCAGTACGCCGACTTGTTTCTGGCCGCCAAGAATCTGAGCGACGCCAT
CCTGCTGTCCGACATCCTGAGAGTGAACTGCCTGAGCTACGATACCGAGATCCTGACCGTGGAATACG
GCTTCCTGCCTATCGGCAAGATCGTCGAGGAACGGATCGAGTGCACAGTGTACACCGTGGATAAGAAT
GGCTTCGTGTACACCCAGCCTATCGCTCAGTGGCACAACAGAGGCGAGCAAGAGGTGTTCGAGTACTG
CCTGGAAGATGGCAGCATCATCCGGGCCACCAAGGACCACAAGTTTATGACCACCGACGGCCAGATGC
TGCCCATCGACGAGATCTTTGAGAGAGGCCTGGACCTGAAACAGGTGGACGGACTGCCTAGCGGCGGA
TCTGAAGGCGCTGATAAAAGAACCGCCGATGGCTCCGAGTTCGAGAGCCCTAAGAAAAAGCGGAAGGT
GTGAAAGCTTGATAATCAACCTCTGGATTACAAAATTTGTGAAAGATTGACTGGTATTCTTAACTATG
TTGCTCCTTTTACGCTATGTGGATACGCTGCTTTAATGCCTTTGTATCATGCTATTGCTTCCCGTATG
GCTTTCATTTTCTCCTCCTTGTATAAATCCTGGTTAGTTCTTGCCACGGCGGAACTCATCGCCGCCTG
CCTTGCCCGCTGCTGGACAGGGGCTCGGCTGTTGGGCACTGACAATTCCGTGGTGTTTATTTGTGAAA
TTTGTGATGCTATTGCTTTATTTGTAACCATCTAGCTTTATTTGTGAAATTTGTGATGCTATTGCTTT
ATTTGTAACCATTATAAGCTGCAATAAACAAGTTAACAACAACAATTGCATTCATTTTATGTTTCAGG
TTCAGGGGGAGATGTGGGAGGTTTTTTAAAGCCCTGCAGGtgtacaaaaaagcaggctttaaaggaac
caattcagtcgactggatccggtaccaaggtcgggcaggaagagggcctatttcccatgattccttca
tatttgcatatacgatacaaggctgttagagagataattagaattaatttgactgtaaacacaaagat
attagtacaaaatacgtgacgtagaaagtaataatttcttgggtagtttgcagttttaaaattatgtt
ttaaaatggactatcatatgcttaccgtaacttgaaagtatttcgatttcttggctttatatatcttg
tggaaaggacgaaacaccGTGTCGAAGTTCGCCCTGGAGGTTTTAGAGCTAGAAATAGCAAGTTAAAA
TAAGGCTAGTCCGTTATCAACTTGAAAAAGTGGCACCGAGTCGGTGCTTTTTT

SABE2(C) vector sequence

CMV promoter, bpNLS, Cfa intein(C), Cas9(C), W3-late SV40 polyA

acattgattattgactagttattaatagtaatcaattacggggtcattagttcatagcccatatatgg
agttccgcgttacataacttacggtaaatggcccgcctggctgaccgcccaacgacccccgcccattg
acgtcaataatgacgtatgttcccatagtaacgccaatagggactttccattgacgtcaatgggtgga
gtatttacggtaaactgcccacttggcagtacatcaagtgtatcatatgccaagtacgccccctattg
acgtcaatgacggtaaatggcccgcctggcattatgcccagtacatgaccttatgggactttcctact
tggcagtacatctacgtattagtcatcgctattaccatggtgatgcggttttggcagtacatcaatgg
gcgtggatagcggtttgactcacggggatttccaagtctccaccccattgacgtcaatgggagtttgt
tttggcaccaaaatcaacgggactttccaaaatgtcgtaacaactccgccccattgacgcaaatgggc
ggtaggcgtgtacggtgggaggtctatataagcagagctggtttagtgaaccgtcagatccgctagcg
ccaccATGGAAGGAGCTGATAAGCGGACAGCCGACGGCAGCGAGTTCGAGAGCCCTAAGAAGAAAAGA
AAGGTGTCCGGCGGATCTGTCAAGATCATCAGCAGAAAGAGCCTGGGCACCCAGAACGTGTACGATAT
CGGAGTGGGCGAGCCCCACAACTTTCTGCTCAAGAATGGCCTGGTGGCCAGCAACTGCGAGATCACCA
AGGCACCTCTGAGCGCCTCTATGATCAAGAGATACGACGAGCACCACCAGGATCTGACCCTGCTGAAG
GCCCTCGTTAGACAGCAGCTGCCAGAGAAGTACAAAGAGATTTTCTTCGACCAGAGCAAGAACGGCTA
CGCCGGCTACATTGATGGCGGAGCCAGCCAAGAGGAATTCTACAAGTTCATCAAGCCCATCCTCGAGA
AGATGGACGGCACCGAGGAACTGCTGGTCAAGCTGAACAGAGAGGACCTGCTGAGAAAGCAGAGAACC
TTCGACAACGGCAGCATCCCTCACCAGATCCACCTGGGAGAACTGCACGCCATTCTGCGGAGACAAGA
GGACTTTTACCCATTCCTGAAGGACAACCGGGAAAAGATCGAGAAAATCCTGACCTTCAGGATCCCCT
ACTACGTGGGACCACTGGCCAGAGGCAATAGCAGATTCGCCTGGATGACCAGAAAGAGCGAGGAAACC
ATCACTCCCTGGAACTTCGAGGAAGTGGTGGACAAGGGCGCCAGCGCTCAGTCCTTCATCGAGCGGAT
GACCAACTTCGATAAGAACCTGCCTAACGAGAAGGTGCTGCCCAAGCACAGCCTGCTGTACGAGTACT
TCACCGTGTACAACGAGCTGACCAAAGTGAAATACGTGACCGAGGGAATGAGAAAGCCCGCCTTTCTG
AGCGGCGAGCAGAAAAAGGCCATCGTGGATCTGCTGTTCAAGACCAACCGGAAAGTGACCGTGAAGCA
GCTGAAAGAGGACTACTTCAAGAAAATCGAGTGCTTCGACAGCGTCGAGATCTCCGGCGTGGAAGATC
GGTTCAATGCCAGCCTGGGCACATACCACGATCTGCTGAAAATTATCAAGGACAAGGACTTCCTGGAC
AACGAAGAGAACGAGGACATCCTTGAGGACATCGTGCTGACACTGACCCTGTTTGAGGACAGAGAGAT
GATCGAGGAACGGCTGAAAACATACGCCCACCTGTTCGACGACAAAGTGATGAAGCAACTGAAGCGGC
GGAGATACACCGGCTGGGGCAGACTGTCTCGGAAGCTGATCAACGGCATCCGGGATAAGCAGTCCGGC
AAGACCATCCTGGACTTTCTGAAGTCCGACGGCTTCGCCAACAGAAACTTCATGCAGCTGATTCACGA
CGACAGCCTCACCTTCAAAGAGGATATCCAGAAAGCCCAGGTGTCCGGCCAGGGCGATTCTCTGCATG
AGCACATTGCCAACCTGGCCGGCTCTCCCGCCATTAAGAAAGGCATCCTGCAGACAGTGAAGGTGGTG
GACGAGCTTGTGAAAGTGATGGGCAGACACAAGCCCGAGAACATCGTGATCGAAATGGCCAGAGAGAA
CCAGACCACACAGAAGGGACAGAAGAACAGCCGCGAGAGAATGAAGCGGATCGAAGAGGGCATCAAAG
AGCTGGGCAGCCAGATCCTGAAAGAACACCCCGTGGAAAACACCCAGCTGCAGAACGAGAAGCTGTAC
CTGTACTACCTGCAGAATGGACGGGATATGTACGTGGACCAAGAGCTGGACATCAACAGACTGTCCGA
CTACGATGTGGACCATATCGTGCCCCAGTCTTTTCTGAAGGACGACTCCATCGACAACAAGGTCCTGA
CCAGATCCGACAAGAATCGGGGCAAGAGCGACAACGTGCCCTCCGAAGAGGTGGTCAAGAAGATGAAG
AACTACTGGCGACAGCTGCTGAACGCCAAGCTGATTACCCAGCGGAAGTTCGACAATCTGACCAAGGC
CGAAAGAGGCGGCCTGAGCGAACTGGATAAGGCCGGCTTCATCAAGAGACAGCTGGTGGAAACCCGGC
AGATCACAAAGCACGTGGCACAGATTCTGGACTCTCGGATGAACACTAAGTACGACGAGAACGACAAA
CTGATCCGCGAAGTGAAAGTCATCACCCTGAAGTCCAAGCTGGTGTCCGATTTCCGGAAGGATTTCCA
GTTCTACAAAGTGCGCGAGATCAACAACTACCATCACGCCCACGACGCCTACCTGAATGCCGTTGTTG
GAACAGCCCTGATCAAAAAGTACCCTAAGCTGGAAAGCGAGTTCGTGTACGGCGACTACAAGGTGTAC
GACGTGCGGAAGATGATCGCCAAGAGCGAGCAAGAGATTGGCAAGGCAACCGCCAAGTACTTCTTCTA
CAGCAACATCATGAACTTTTTCAAGACAGAGATCACCCTCGCCAACGGCGAGATCAGAAAGCGGCCTC
TGATCGAGACAAACGGCGAAACCGGCGAGATTGTGTGGGATAAGGGCAGAGACTTTGCCACAGTGCGG
AAAGTGCTGAGCATGCCCCAAGTGAATATCGTGAAGAAAACCGAGGTGCAGACAGGCGGCTTCAGCAA
AGAGTCTATCCTGCCTAAGCGGAACTCCGACAAGCTGATCGCCAGAAAGAAGGACTGGGACCCCAAGA
AGTACGGCGGCTTCGATTCTCCTACCGTGGCCTATAGCGTGCTGGTGGTGGCCAAAGTGGAAAAGGGC
AAGTCCAAGAAACTCAAGAGCGTGAAAGAGCTGCTGGGGATCACCATCATGGAAAGAAGCAGCTTCGA
GAAGAATCCGATCGATTTCCTCGAGGCCAAGGGCTACAAAGAAGTGAAAAAGGACCTGATCATCAAGC
TCCCCAAGTACTCCCTGTTCGAGCTGGAAAACGGCCGGAAGAGAATGCTGGCCTCTGCTGGCGAACTG

CAGAAGGGAAACGAACTGGCCCTGCCTAGCAAATATGTGAACTTCCTGTACCTGGCCAGCCACTATGA
GAAGCTGAAGGGCAGCCCCGAGGACAATGAGCAAAAGCAGCTGTTTGTGGAACAGCACAAGCACTACC
TGGACGAGATCATCGAGCAGATCAGCGAGTTTAGCAAGAGAGTGATTCTGGCCGACGCCAATCTGGAC
AAAGTGCTGTCCGCCTACAACAAGCACCGGGACAAGCCTATCAGAGAGCAGGCCGAGAATATCATCCA
CCTGTTTACCCTGACCAACCTGGGAGCCCCTGCCGCCTTCAAGTACTTTGACACCACCATCGACCGGA
AGCGGTACACCTCCACCAAAGAGGTGCTGGACGCCACTCTGATCCACCAGTCTATCACCGGCCTGTAC
GAGACACGGATCGACCTGTCTCAACTCGGAGGCGACGAAGGCGCCGATAAGAGAACCGCCGATGGCTC
TGAGTTCGAGAGCCCCAAGAAAAAGCGCAAAGTGTGAAAGCTTGATAATCAACCTCTGGATTACAAAA
TTTGTGAAAGATTGACTGGTATTCTTAACTATGTTGCTCCTTTTACGCTATGTGGATACGCTGCTTTA
ATGCCTTTGTATCATGCTATTGCTTCCCGTATGGCTTTCATTTTCTCCTCCTTGTATAAATCCTGGTT
AGTTCTTGCCACGGCGGAACTCATCGCCGCCTGCCTTGCCCGCTGCTGGACAGGGGCTCGGCTGTTGG
GCACTGACAATTCCGTGGTGTTTATTTGTGAAATTTGTGATGCTATTGCTTTATTTGTAACCATCTAG
CTTTATTTGTGAAATTTGTGATGCTATTGCTTTATTTGTAACCATTATAAGCTGCAATAAACAAGTTA
ACAACAACAATTGCATTCATTTTATGTTTCAGGTTCAGGGGGAGATGTGGAGGTTTTTTAAAGC

SABE3(N) vector sequence

CBA promoter, bpNLS, TadA8.5-m, Cas9(N), Cfa intein(N), bGH polyA, U6 promoter, gRNA

tcgttacataacttacggtaaatggcccgcctggctgaccgcccaacgacccccgcccattgacgtca
ataatgacgtatgttcccatagtaacgccaatagggactttccattgacgtcaatgggtggagtattt
acggtaaactgcccacttggcagtacatcaagtgtatcatatgccaagtacgccccctattgacgtca
atgacggtaaatggcccgcctggcattatgcccagtacatgaccttatgggactttcctacttggcag
tacatctactcgaggccacgttctgcttcactctccccatctcccccccctccccacccccaattttg
tatttatttattttttaattattttgtgcagcgatgggggcgggggggggggggggcgcgcgccagg
cggggcggggcggggcgaggggcggggcggggcgaggcggagaggtgcggcggcagccaatcagagcg
gcgcgctccgaaagtttccttttatggcgaggcggcggcggcggcggccctataaaaagcgaagcgcg
cggcgggcggtaagtatcaaggttacaagacaggtttaaggagaccaatagaaactgggcttgtcgag
acagagaagactcttgcgtttctgataggcacctattggtcttactgacatccactttgcctttctct
ccacaggctagcgccaccATGGAAGGCGCCGATAAGCGGACCGCCGACGGCAGCGAGTTCGAGAGCCC
TAAGAAAAAGAGGAAGGTGAGCGAGGTCGAGTTCTCTCACGAATATTGGATGAGACACGCTCTCACCC
TGGCTAAGAGAGCCAGGGACGAAAGAGAGGTGCCAGTTGGCGCTGTCCTGGTGTTGAACAATCGCGTC
ATCGGAGAAGGATGGAATCGCGCCATTGGCCTGCACGATCCAACCGCACATGCCGAAATTATGGCTCT
GCGGCAAGGCGGCCTCGTGATGCAAAATTACAGACTGATCGATGCTACCCTCTACAGCACCTTCGAGC
CCTGTGTCATGTGTGCTGGGGCAATGATTCACTCCCGGATTGGCCGCGTGGTGTTTGGAGTGCGGAAT
GCCAAGACTGGCGCCGCTGGATCTCTGATGGACGTCCTGCACTATCCTGGGATGAACCACCGGGTCGA
GATCACAGAGGGAATTCTGGCTGACGAGTGCGCTGCCCTGCTGTGCTACTTCTTTAGAATGCCCAGAC
AGGTGTTCAACGCCCAGAAAAAAGCTCAGAGCAGCACCGATTCCGGCGGAAGCAGCGGAGGATCTTCT
GGAAGCGAAACCCCAGGCACCAGCGAGTCTGCCACACCAGAATCATCTGGCGGTAGCTCCGGCGGCAG
CGACAAGAAGTATTCTATCGGACTGGCCATCGGCACCAACTCTGTTGGATGGGCCGTGATCACCGACG
AGTACAAGGTGCCCAGCAAGAAATTCAAGGTGCTGGGCAACACCGACAGGCACAGCATCAAGAAGAAC
CTGATCGGCGCACTGCTGTTCGACTCTGGCGAAACAGCCGAGGCCACCAGACTGAAGAGAACAGCCCG
CAGACGGTACACCAGAAGAAAGAACCGGATCTGCTACCTCCAAGAGATCTTCAGCAACGAGATGGCCA
AGGTGGACGACAGCTTCTTCCACAGACTGGAAGAGTCCTTCCTGGTGGAAGAGGACAAGAAGCACGAG
AGACACCCCATCTTCGGCAACATCGTGGACGAGGTGGCCTACCACGAGAAGTACCCCACCATCTACCA
CCTGAGAAAGAAACTGGTGGACAGCACCGACAAGGCCGACCTGAGACTGATCTATCTGGCCCTGGCTC
ACATGATCAAGTTCCGGGGCCACTTCCTGATCGAGGGCGACCTGAATCCTGACAACAGCGACGTGGAC
AAGCTGTTCATCCAGCTGGTGCAGACCTACAACCAGCTGTTCGAGGAAAACCCCATCAACGCCAGCGG
AGTGGATGCCAAGGCCATCCTGTCTGCCAGACTGAGCAAGAGCAGACGGCTGGAAAATCTGATCGCCC
AGCTGCCTGGCGAGAAGAAGAATGGCCTGTTCGGCAACCTGATTGCCCTGAGCCTGGGCCTGACACCT
AACTTCAAGAGCAACTTCGACCTGGCCGAGGACGCCAAACTGCAGCTGAGCAAGGACACCTACGACGA
CGACCTGGACAATCTGCTGGCCCAGATCGGCGATCAGTACGCCGACTTGTTTCTGGCCGCCAAGAATC
TGAGCGACGCCATCCTGCTGTCCGACATCCTGAGAGTGAACTGCCTGAGCTACGATACCGAGATCCTG
ACCGTGGAATACGGCTTCCTGCCTATCGGCAAGATCGTCGAGGAACGGATCGAGTGCACAGTGTACAC
CGTGGATAAGAATGGCTTCGTGTACACCCAGCCTATCGCTCAGTGGCACAACAGAGGCGAGCAAGAGG
TGTTCGAGTACTGCCTGGAAGATGGCAGCATCATCCGGGCCACCAAGGACCACAAGTTTATGACCACC
GACGGCCAGATGCTGCCCATCGACGAGATCTTTGAGAGAGGCCTGGACCTGAAACAGGTGGACGGACT
GCCTAGCGGCGGATCTGAAGGCGCTGATAAAAGAACCGCCGATGGCTCCGAGTTCGAGAGCCCTAAGA
AAAAGCGGAAGGTGTGAAAGCTTCGACTGTGCCTTCTAGTTGCCAGCCATCTGTTGTTTGCCCCTCCC
CCGTGCCTTCCTTGACCCTGGAAGGTGCCACTCCCACTGTCCTTTCCTAATAAAATGAGGAAATTGCA
TCGCATTGTCTGAGTAGGTGTCATTCTATTCTGGGGGGTGGGGTGGGGCAGGACAGCAAGGGGGAGGA
TTGGGAAGACAATAGCAGGCATGCTGGGGACCTGCAGGtgtacaaaaaagcaggctttaaaggaacca
attcagtcgactggatccggtaccaaggtcgggcaggaagagggcctatttcccatgattccttcata
tttgcatatacgatacaaggctgttagagagataattagaattaatttgactgtaaacacaaagatat
tagtacaaaatacgtgacgtagaaagtaataatttcttgggtagtttgcagttttaaaattatgtttt
aaaatggactatcatatgcttaccgtaacttgaaagtatttcgatttcttggctttatatatcttgtg
gaaaggacgaaacaccGTGTCGAAGTTCGCCCTGGAGGTTTTAGAGCTAGAAATAGCAAGTTAAAATA
AGGCTAGTCCGTTATCAACTTGAAAAAGTGGCACCGAGTCGGTGCTTTTTT

SABE3(C) vector sequence

CBA promoter, bpNLS, Cfa intein(C), Cas9(C), bGH polyA

tcgttacataacttacggtaaatggcccgcctggctgaccgcccaacgacccccgcccattgacgtca
ataatgacgtatgttcccatagtaacgccaatagggactttccattgacgtcaatgggtggagtattt
acggtaaactgcccacttggcagtacatcaagtgtatcatatgccaagtacgccccctattgacgtca
atgacggtaaatggcccgcctggcattatgcccagtacatgaccttatgggactttcctacttggcag
tacatctactcgaggccacgttctgcttcactctccccatctcccccccctccccacccccaatttttg
tatttatttattttttaattattttgtgcagcgatgggggcgggggggggggggggcgcgcgccagg
cggggcggggcggggcgaggggcggggcggggcgaggcggagaggtgcggcggcagccaatcagagcg
gcgcgctccgaaagtttccttttatggcgaggcggcggcggcggcggccctataaaaagcgaagcgcg
cggcgggcggtaagtatcaaggttacaagacaggtttaaggagaccaatagaaactgggcttgtcgag
acagagaagactcttgcgtttctgataggcacctattggtcttactgacatccactttgcctttctct
ccacaggctagcgccaccATGGAAGGAGCTGATAAGCGGACAGCCGACGGCAGCGAGTTCGAGAGCCC
TAAGAAGAAAAGAAAGGTGTCCGGCGGATCTGTCAAGATCATCAGCAGAAAGAGCCTGGGCACCCAGA
ACGTGTACGATATCGGAGTGGGCGAGCCCCACAACTTTCTGCTCAAGAATGGCCTGGTGGCCAGCAAC
TGCGAGATCACCAAGGCACCTCTGAGCGCCTCTATGATCAAGAGATACGACGAGCACCACCAGGATCT
GACCCTGCTGAAGGCCCTCGTTAGACAGCAGCTGCCAGAGAAGTACAAAGAGATTTTCTTCGACCAGA
GCAAGAACGGCTACGCCGGCTACATTGATGGCGGAGCCAGCCAAGAGGAATTCTACAAGTTCATCAAG
CCCATCCTCGAGAAGATGGACGGCACCGAGGAACTGCTGGTCAAGCTGAACAGAGAGGACCTGCTGAG
AAAGCAGAGAACCTTCGACAACGGCAGCATCCCTCACCAGATCCACCTGGGAGAACTGCACGCCATTC
TGCGGAGACAAGAGGACTTTTACCCATTCCTGAAGGACAACCGGGAAAAGATCGAGAAAATCCTGACC
TTCAGGATCCCCTACTACGTGGGACCACTGGCCAGAGGCAATAGCAGATTCGCCTGGATGACCAGAAA
GAGCGAGGAAACCATCACTCCCTGGAACTTCGAGGAAGTGGTGGACAAGGGCGCCAGCGCTCAGTCCT
TCATCGAGCGGATGACCAACTTCGATAAGAACCTGCCTAACGAGAAGGTGCTGCCCAAGCACAGCCTG
CTGTACGAGTACTTCACCGTGTACAACGAGCTGACCAAAGTGAAATACGTGACCGAGGGAATGAGAAA
GCCCGCCTTTCTGAGCGGCGAGCAGAAAAAGGCCATCGTGGATCTGCTGTTCAAGACCAACCGGAAAG
TGACCGTGAAGCAGCTGAAAGAGGACTACTTCAAGAAAATCGAGTGCTTCGACAGCGTCGAGATCTCC
GGCGTGGAAGATCGGTTCAATGCCAGCCTGGGCACATACCACGATCTGCTGAAAATTATCAAGGACAA
GGACTTCCTGGACAACGAAGAGAACGAGGACATCCTTGAGGACATCGTGCTGACACTGACCCTGTTTG
AGGACAGAGAGATGATCGAGGAACGGCTGAAAACATACGCCCACCTGTTCGACGACAAAGTGATGAAG
CAACTGAAGCGGCGGAGATACACCGGCTGGGGCAGACTGTCTCGGAAGCTGATCAACGGCATCCGGGA
TAAGCAGTCCGGCAAGACCATCCTGGACTTTCTGAAGTCCGACGGCTTCGCCAACAGAAACTTCATGC
AGCTGATTCACGACGACAGCCTCACCTTCAAAGAGGATATCCAGAAAGCCCAGGTGTCCGGCCAGGGC
GATTCTCTGCATGAGCACATTGCCAACCTGGCCGGCTCTCCCGCCATTAAGAAAGGCATCCTGCAGAC
AGTGAAGGTGGTGGACGAGCTTGTGAAAGTGATGGGCAGACACAAGCCCGAGAACATCGTGATCGAAA
TGGCCAGAGAGAACCAGACCACACAGAAGGGACAGAAGAACAGCCGCGAGAGAATGAAGCGGATCGAA
GAGGGCATCAAAGAGCTGGGCAGCCAGATCCTGAAAGAACACCCCGTGGAAAACACCCAGCTGCAGAA
CGAGAAGCTGTACCTGTACTACCTGCAGAATGGACGGGATATGTACGTGGACCAAGAGCTGGACATCA
ACAGACTGTCCGACTACGATGTGGACCATATCGTGCCCCAGTCTTTTCTGAAGGACGACTCCATCGAC
AACAAGGTCCTGACCAGATCCGACAAGAATCGGGGCAAGAGCGACAACGTGCCCTCCGAAGAGGTGGT
CAAGAAGATGAAGAACTACTGGCGACAGCTGCTGAACGCCAAGCTGATTACCCAGCGGAAGTTCGACA
ATCTGACCAAGGCCGAAAGAGGCGGCCTGAGCGAACTGGATAAGGCCGGCTTCATCAAGAGACAGCTG
GTGGAAACCCGGCAGATCACAAAGCACGTGGCACAGATTCTGGACTCTCGGATGAACACTAAGTACGA
CGAGAACGACAAACTGATCCGCGAAGTGAAAGTCATCACCCTGAAGTCCAAGCTGGTGTCCGATTTCC
GGAAGGATTTCCAGTTCTACAAAGTGCGCGAGATCAACAACTACCATCACGCCCACGACGCCTACCTG
AATGCCGTTGTTGGAACAGCCCTGATCAAAAAGTACCCTAAGCTGGAAAGCGAGTTCGTGTACGGCGA
CTACAAGGTGTACGACGTGCGGAAGATGATCGCCAAGAGCGAGCAAGAGATTGGCAAGGCAACCGCCA
AGTACTTCTTCTACAGCAACATCATGAACTTTTTCAAGACAGAGATCACCCTCGCCAACGGCGAGATC
AGAAAGCGGCCTCTGATCGAGACAAACGGCGAAACCGGCGAGATTGTGTGGGATAAGGGCAGAGACTT
TGCCACAGTGCGGAAAGTGCTGAGCATGCCCCAAGTGAATATCGTGAAGAAAACCGAGGTGCAGACAG
GCGGCTTCAGCAAAGAGTCTATCCTGCCTAAGCGGAACTCCGACAAGCTGATCGCCAGAAAGAAGGAC
TGGGACCCCAAGAAGTACGGCGGCTTCGATTCTCCTACCGTGGCCTATAGCGTGCTGGTGGTGGCCAA
AGTGGAAAAGGGCAAGTCCAAGAAACTCAAGAGCGTGAAAGAGCTGCTGGGGATCACCATCATGGAAA
GAAGCAGCTTCGAGAAGAATCCGATCGATTTCCTCGAGGCCAAGGGCTACAAAGAAGTGAAAAAGGAC

CTGATCATCAAGCTCCCCAAGTACTCCCTGTTCGAGCTGGAAAACGGCCGGAAGAGAATGCTGGCCTC
TGCTGGCGAACTGCAGAAGGGAAACGAACTGGCCCTGCCTAGCAAATATGTGAACTTCCTGTACCTGG
CCAGCCACTATGAGAAGCTGAAGGGCAGCCCCGAGGACAATGAGCAAAAGCAGCTGTTTGTGGAACAG
CACAAGCACTACCTGGACGAGATCATCGAGCAGATCAGCGAGTTTAGCAAGAGAGTGATTCTGGCCGA
CGCCAATCTGGACAAAGTGCTGTCCGCCTACAACAAGCACCGGGACAAGCCTATCAGAGAGCAGGCCG
AGAATATCATCCACCTGTTTACCCTGACCAACCTGGGAGCCCCTGCCGCCTTCAAGTACTTTGACACC
ACCATCGACCGGAAGCGGTACACCTCCACCAAAGAGGTGCTGGACGCCACTCTGATCCACCAGTCTAT
CACCGGCCTGTACGAGACACGGATCGACCTGTCTCAACTCGGAGGCGACGAAGGCGCCGATAAGAGAA
CCGCCGATGGCTCTGAGTTCGAGAGCCCCAAGAAAAAGCGCAAAGTGTGAAAGCTTCGACTGTGCCTT
CTAGTTGCCAGCCATCTGTTGTTTGCCCCTCCCCCGTGCCTTCCTTGACCCTGGAAGGTGCCACTCCC
ACTGTCCTTTCCTAATAAAATGAGGAAATTGCATCGCATTGTCTGAGTAGGTGTCATTCTATTCTGGG
GGGTGGGGTGGGGCAGGACAGCAAGGGGGAGGATTGGGAAGACAATAGCAGGCATGCTGGGGA

Extended Data Figures Legends

Extended Data Fig. 1 | c.5882G>A haplotype categories and percent of patients who have a fully correctable allele by SABE. Our analysis is based on published genetic data from 150 Stargardt patients mostly from European descent[6]. **A)** Possible haplotypes found in Stargardt patient with at least one allele including c.5882G>A. **B)** Counts and percentage of different haplotypes found in the published study. **C)** Fraction of patients who have at least one allele, which is fully or partially correctable by SABE. **D)** Pie chart showing fraction of STGD patients carrying the p.G1961E mutation who have at least one allele, which is fully (green) or partially (red) correctable by SABE. The calculation is based on Lee W et al.[6] and was applied on a large number of patients analyzed by Cornelis SS et al[50]. **E)** Depiction of possible *ABCA4* genotypes with respect to SABE-mediated correction. Concerning patients with homozygous haplotype 1, we expect other yet unknown modifier(s) (labeled with question mark) since homozygosity for c.5882G>A alone was shown not to lead to disease[51]. Therefore, we considered haplotype 1 as "partially correctable". VUS, variant of uncertain significance

Extended Data Fig. 2 | Adenine base-editing efficiency by different gRNAs. A) Schematic of a fragment of the *ABCA4* exon 42 with the NGG-PAM sequence indicated in purple, the Stargardt c.5882A target base highlighted in red, and the c.5883A wobble base shown in blue. **B)** Base-editing efficiencies at the target- and wobble bases with the unsplit ABE7.10 base editor in combination with the different gRNAs in lenti-$ABCA4^{1961E}$ HEK293T cells. Results were obtained from three biological replicates (eyes) and are presented as mean ± SD. *$P < 0.05$, ***$P < 0.001$ by three-way mixed-effect ANOVA with Tukey's correction.

Extended Data Fig. 3 | Conservation of the *ABCA4* sequence around p.Gly1961 and ABE-mediated cytosine editing on the *ABCA4* gene. A) The table shows the sequence alignment between humans and 10 other vertebrates. The first row shows the human *ABCA4* reference sequence. All sequence changes to the human sequence are indicated in bold. The second row shows the *ABCA4* c.5882A allele, with the A7 target base highlighted in red. The third and fourth rows show the most frequent base-editing outcomes in our study. The two observed bystander edits, c.5880C to c.5880T at position five (c.5880C>T, p.Val1960=) and c.5883A to c.5883G at position eight (c.5883A>G, p.Gly1961=) lead to silent changes, do not affect conserved base positions, and are present in other species. These results suggest that these bystander base changes have no biological relevance. **B)** Fragment of the *ABCA4* exon 42 with the base-editor window highlighted in grey. The base-editor window also contains a TC

sequence that constitutes a possible motif for ABE-mediated cytosine editing. *ABCA4* c.5880C to c.5880T bystander editing results in a silent change (c.5880C>T, p.Val1960=). This change is not conserved and is expected to have no biological relevance. **C)** Adenine base-editing efficiencies at the A8 site and cytosine base-editing efficiencies at the C5 site with different dual AAV-ABE versions in human iPSC-RPE. Results were obtained from two biological replicates and are presented as mean.

Extended Data Fig. 4 | Generation of the $ABCA4^{G1961E}$ human mutant iPSC line. A) Strategy for the generation of an $ABCA4^{G1961E}$ iPSC line. *ABCA4* exon 42 (blue box) with flanking introns (top). The PAM site is highlighted in purple, and the gRNA binding site is indicated by a black dashed line. The black arrowheads point to the PAM disruption site (silent mutation) and the red arrowheads point to the *ABCA4* c.5882G>A mutation. Representative Sanger sequencing trace of the $ABCA4^{1961E/E}$ clone that was selected for human retinal organoid induction (bottom). **B)** Results from targeted deep-sequencing of the $ABCA4^{1961G/E}$ (top) and $ABCA4^{1961E/E}$ (bottom) clone confirming successful knock-in of the target mutation in a heterozygous or homozygous form. **C)** Results from the iPSC digital aneuploidy test, confirming the genomic integrity of the $ABCA4^{1961G/E}$ (top) and $ABCA4^{1961E/E}$ (bottom) clone. These clones were used for human retinal organoid induction. **D)** Confocal images of $ABCA4^{1961G/E}$ (left) and $ABCA4^{1961E/E}$ (right) iPSCs. Green: antibody for pluripotency markers (NANOG, SOX2, OCT4, SSEA4); grey: Hoechst (scale bars: 100 µm). The experiment was performed once with one biological replicate.

Extended Data Fig. 5 | Characterization of the $ABCA4^{G1961E}$ human retinal organoids. A) Confocal images of $ABCA4^{1961G/G}$ (left), $ABCA4^{1961G/E}$ (middle), and $ABCA4^{1961E/E}$ (right) human retinal organoids. Grey: Hoechst; cyan: rhodopsin; magenta: arrestin3 (scale bars: 25 µm). **B)** 2D UMAP projection of single cells from human retinal organoids ordered by the *ABCA4* genotype ($ABCA4^{1961G/G}$ human retinal organoids: left; $ABCA4^{1961G/E}$ human retinal organoids: middle, and $ABCA4^{1961E/E}$ human retinal organoids: right) and the developmental stage (immature: top; mature: bottom). **C)** 2D UMAP plot of scRNA data from mature human retinal organoids colored by cell type and plotted separately by the *ABCA4* genotype ($ABCA4^{1961G/G}$ human retinal organoids: left; $ABCA4^{1961G/E}$ human retinal organoids: middle, and $ABCA4^{1961E/E}$ human retinal organoids: right). **D)** Heat map for Jensen–Shannon divergence (JSD) showing the similarity between organoids of different *ABCA4* genotypes at two different developmental stages (immature: top; mature: bottom). **E)** Results from targeted

deep-sequencing of the *ABCA4*$^{1961G/E}$ clone confirming successful knock-in of the target mutation in a heterozygous form. Confocal images of un-fed and POS-fed iPSC-RPE cells. Grey: ZO-1; magenta: ceramide (scale bars: 25 µm). Quantification of ceramide intensity signal, for un-fed and POS-fed iPSC-RPE. **F)** Genotypes of patient iPSC-RPE cells. Quantification of **(G)** ceramide intensity signal, **(H)** filipin-stained lipid deposits and **(I)** BODIPY-stained lipid deposits (one outlier point in control 2 was removed for plotting) for un-fed and POS-fed controls and patient-derived iPSC-RPE cells. For BODIPY-stained lipid deposits. Statistical tests used were two-sided, *$P = 0.0296$ for the interaction between feeding condition and sample (control and patient) in the ANOVA model with the count in fed condition being higher than in unfed condition in patient and lower in control. For Extended Data Fig. 5G-I, the experiment was performed twice with two biological replicates and results are presented as mean ± SD.

Extended Data Fig. 6 | Generation and characterization of *Abca4*^{hu1961E} mice. A) Strategy for the generation of the *Abca4*^{hu1961E} mouse line. *Abca4* exon 42 (blue box) with flanking introns. The PAM sites are highlighted, and the gRNA binding sites are indicated by black dashed lines. The red arrowhead points to the G1961E mutation. Bold nucleotides indicate nucleotide changes due to humanization. Note the deletion in the downstream intron – this is intentional and was introduced to disrupt the PAM site. The deletion is not expected to interfere with splicing as it is at position +9, at which there is no base preference for canonical splicing[52]. **B)** Sequencing of the gDNA of the *Abca4*^{hu1961E} allele. The red arrowhead points to the *Abca4* c.5882G>A mutation, the black arrowhead points to a deletion in the intron. **C)** Deep-sequencing of *Abca4*$^{hu1961E/ms1961G(KO)}$ mice, where the results indicate heterozygosity. The red arrowhead points to the *Abca4* c.5882G>A mutation and the black arrowheads point to the nucleotide changes due to humanization. **D)** Retinoid and bisretinoid levels measured by LC-HRMS analysis of eyes of *Abca4*$^{hu1961E/ms1961G(KO)}$ mice compared to age-matched *Abca4*$^{ms1961G/ms1961G(KO)}$ littermates. Retinyl acetate was used as internal standard (IS) for normalization. Absolute levels of A2E (A2E targeted) were determined according to the standard curve for synthetic A2E. Results were obtained from four biological replicates, except for atROL for mutant animals, where 3 biological replicates were used (9 months). Results were obtained from six biological replicates, except for atROL, where 4 biological replicates were used (11 months). Results are presented as mean ± SD. Statistical tests used were two-sided, A2E targeted: *$P = 0.021$, A2GPE: *$P = 0.016$, dimeric atRAL: **$P = 0.002$, atROL: $P = 0.056$ for the interaction between genotype and age in the ANOVA model. **E)** Fundus

autofluorescence images of retinas from an *Abca4*$^{hu1961E/ms1961G(KO)}$ and wild-type *Abca4*$^{ms1961G/G}$ mouse (top). Quantification of the fluorescent signals at different ages (bottom). For *Abca4*$^{hu1961E/ms1961G(KO)}$ animals, results were obtained from eight (week 20), nine (week 33) and five (week 44) biological replicates (eyes). For wild-type *Abca4*$^{ms1961G/G}$ animals, results were obtained from eight (week 20), two (week 33) and five (week 44) biological replicates (eyes). **F)** Confocal images of sections from *Abca4*$^{hu1961E/ms1961G(KO)}$ mice. Grey: Hoechst; cyan: rhodopsin, magenta: ABCA4 (scale bar: 25 μm).

Extended Data Fig. 7 | Dual AAV base editing in different in vitro model systems. A) Comparison of in vitro base-editing efficiencies at the A8 site in gDNA with different ubiquitous and photoreceptor specific promoters in *ABCA4*$^{1961G/G}$ human retinal organoids. Results were obtained from three biological replicates and are presented as mean ± SD. $^*P < 0.05$ by one-way ANOVA with Tukey's multiple comparisons test. CMV: cytomegalovirus promoter, CBA: chicken β-actin promoter, ProA7: cone-specific promoter from[19], hGRK1: human rhodopsin-kinase promoter. **B)** AAV9-PHP.eB-SABE1 editing efficiencies at the A8 site in gDNA of human iPSC-RPE at different time points and two different doses (high dose = 10^6 v.g./cell and low dose = 10^5 v.g./cell). Results were obtained from four biological replicates and are presented as mean ± SD. **C)** AAV9-PHP.eB-SABE1 editing at the A7 and A8 sites in gDNA of *ABCA4*$^{1961E/E}$ human retinal organoids. Results were obtained from five biological replicates and are presented as mean ± SD. **D)** AAV5-v2-SABE1 editing efficiencies at the A8 site in gDNA, *ABCA4* mRNA and sorted cones and rods of human retinal explants 5 weeks post-transduction. Results were obtained from three biological replicates and are presented as mean ± SD. **E)** AAV5-v2-SABE1 editing efficiencies at the A8 site in gDNA and *ABCA4* mRNA of human RPE/choroid explants 5 weeks post-transduction. Results were obtained from three biological replicates and are presented as mean ± SD.

Extended Data Fig. 8 | In vitro AAV capsid screen (low dose). A) Representative images of *ABCA4*$^{1961G/G}$ human retinal explants transduced with different AAV capsids encoding for CMV-eGFP. Results are from 5 weeks after transduction (4.7×10^{10} v.g./explant). Efficient cone-photoreceptor transduction is shown by colocalization of eGFP with arrestin3 (merge) (scale bars: 25 μm). **B)** Representative images of *ABCA4*$^{1961G/G}$ human retinal organoids transduced with the same capsids 4 weeks after transduction (3×10^{10} v.g./organoid). Efficient cone-photoreceptor transduction is shown by colocalization of eGFP with arrestin3 (merge)

(scale bars: 50 µm). **C)** Representative images of *ABCA4*$^{1961G/G}$ human RPE/choroid explants transduced with AAV5- or AAV9-PHP.eB capsids 5 weeks after transduction (4.7 × 10^{10} v.g./explant) (scale bars: 25 µm). Grey: Hoechst; magenta: arrestin3; green: eGFP. The number of independent experiments and biological replicates are included in the Methods section, under 'Statistics and Reproducibility'.

Extended Data Fig. 9 | In vitro AAV capsid screen (high dose). A) Representative images of *ABCA4*$^{1961G/G}$ human retinal explants transduced with different AAV capsids encoding for CMV-eGFP. Results are from 5 weeks after transduction (2.5 × 10^{11} v.g./explant). Efficient cone-photoreceptor transduction is shown by colocalization of eGFP with arrestin3 (merge) (scale bars: 25 µm). **B)** Representative images of *ABCA4*$^{1961G/G}$ human retinal organoids transduced with the same capsids 4 weeks after transduction (1.15 × 10^{11} v.g./organoid). Efficient cone-photoreceptor transduction is shown by colocalization of eGFP with arrestin3 (merge) (scale bars: 50 µm). **C)** Representative images of *ABCA4*$^{1961G/G}$ human RPE/choroid explants transduced with AAV5- or AAV9-PHP.eB capsids 5 weeks after transduction (2.5 × 10^{11} v.g./explant) (scale bars: 25 µm). Grey: Hoechst; magenta: arrestin3; green: eGFP. The number of independent experiments and biological replicates are included in the Methods section, under 'Statistics and Reproducibility' section.

Extended Data Fig. 10 | In vivo *ABCA4* base editing in non-ocular tissues from subretinally injected mice and NHPs. A) In vivo base-editing efficiencies in mouse tissues at the A7 and A8 site in *Abca4* gDNA and mRNA in the retina and RPE/choroid/sclera as well as in *Abca4* gDNA of different tissues of the visual pathway, cortex, cerebellum and peripheral organs. Tissues were harvested 5 weeks after subretinal injection. Results were obtained from two biological replicates (eyes) and one replicate (non-occular tissues). Results are presented as mean. **B)** In vivo base-editing efficiencies in NHPs at the A8 site in *ABCA4* gDNA and mRNA in the retina and RPE/choroid as well as in *ABCA4* gDNA of different tissues of the visual pathway, cortex, cerebellum and peripheral organs. Tissues were harvested 20 weeks after subretinal injection. Results were obtained from four biological replicates (eyes) and two biological replicate (non-occular tissues). Results are presented as mean ± SD.

Supplementary Figures

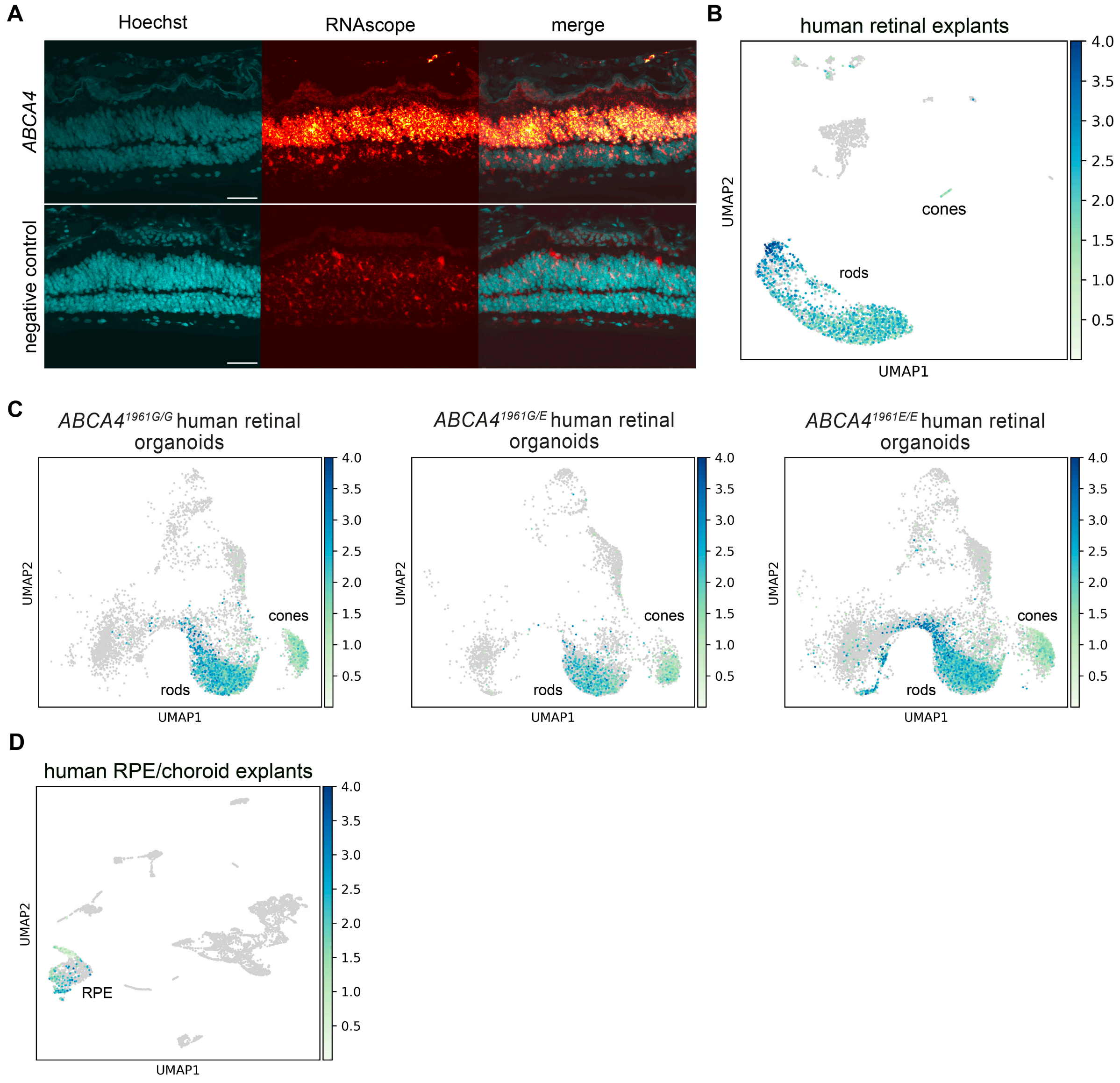

Supplementary Fig. 1 | *ABCA4* expression in human model systems. A) RNAScope from human retina showing *ABCA4* expression in photoreceptors, scale bars: 50 µm. The experiment was performed once with one biological replicate. **B-D)** UMAP clustering of scRNA data, highlighting *ABCA4*-expressing cells in blue. Results show that *ABCA4* is expressed in human cones, rods and RPE cells.

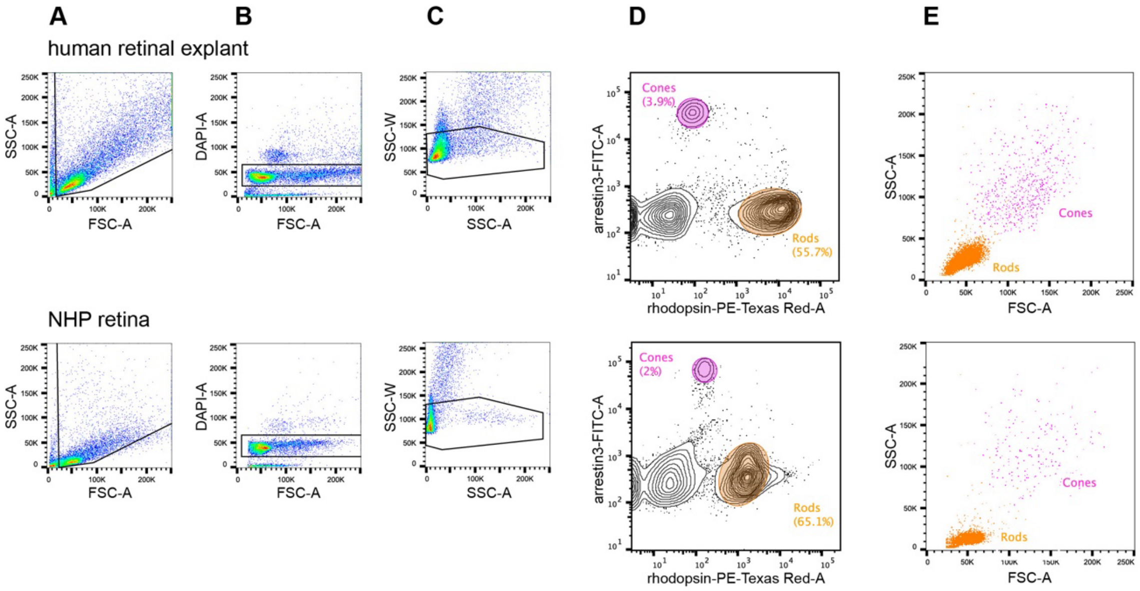

Supplementary Fig. 2 | Isolation of cone- and rod-photoreceptor cells from human retinal explants and NHP retinas. A-C) Representative FACS density plots from a human retinal explant (top) and a NHP retina (bottom) showing the initial gating strategy. **A)** The fixed cell suspension was first analyzed by forward scatter (FSC) and side scatter (SSC). **B)** To separate cells from debris, cells were further gated by Hoechst intensity. **C)** Cells were separated into singlets inside scatter area (SSC-A) versus width (SSC-W) plots. **D)** Contour plots depicting the two sorting gates based on arrestin3 (FITC-A+, cones) and rhodopsin (PE-Texas Red-A+, rods) expression and the frequencies of the two sorted populations. **E)** FACS dot plots showing sorted cones and rods by their size (FSC-A) and granularity (SSC-A), illustrating the expected size difference between the two photoreceptor cell types.

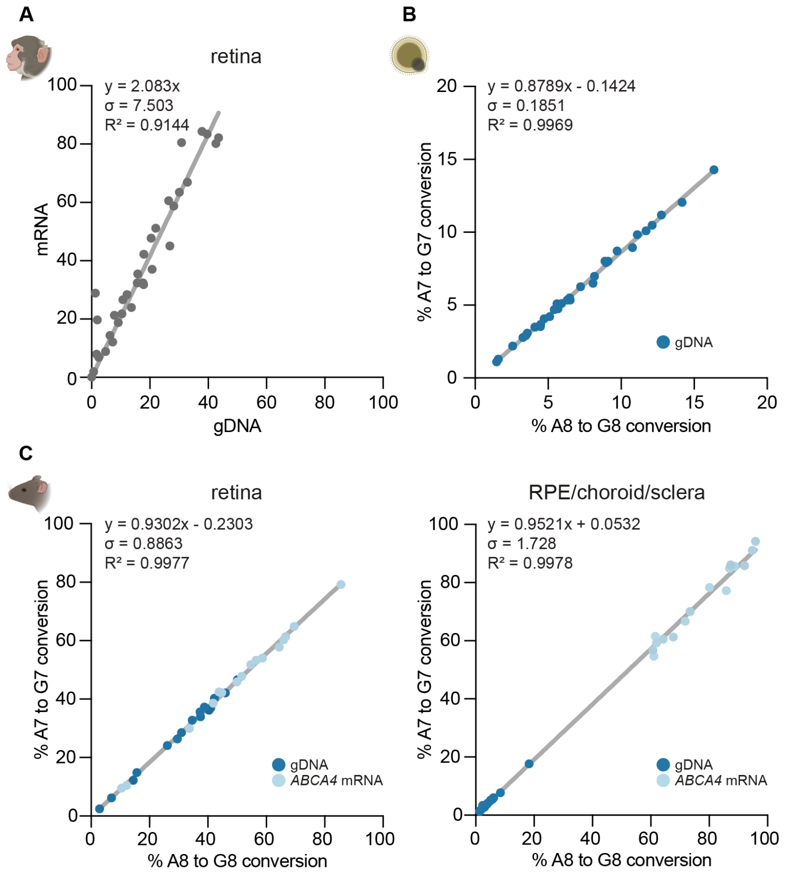

Supplementary Fig. 3 | Prediction of mRNA editing rates from gDNA editing rates and prediction of A7 editing rates from A8 editing rates using a linear regression. A) Relationship between editing rates at the gDNA and mRNA level in the retina of NHPs. Data points were fitted using a simple linear regression. $\beta_1 = 2.083$, p <0.0001 by Wald test. **B)** A7 editing rates as a function of A8 editing rates, from *ABCA4*$^{1961E/E}$ human retinal organoids at the gDNA level. Data points were fitted using a simple linear regression ($y = \beta_1 x + \beta_0$). β_1 is the slope of the regression line, β_0 is the intercept and σ is the residual standard deviation. $\beta_1 =$ 0.8789, p <0.0001 by Wald test. **C)** A7 editing rates as a function of A8 editing rates, from *Abca4*$^{hu1961E/ms1961G(KO)}$ at the gDNA level and *ABCA4* mRNA level in the retina (left) and RPE/choroid/sclera (right). Data points were fitted using a simple linear regression. $\beta_1 =$ 0.9302, *P* <0.0001 for the retina and $\beta_1 = 0.9521$, *P* <0.0001 for the RPE/choroid/sclera. The *P*-value was determined by Wald test. Based on the organoid **B)** and mouse **C)** data, we found that A7 editing rate is on average 92% of A8 editing rate.

Supplementary Data Source File

Source Data Fig 3C.

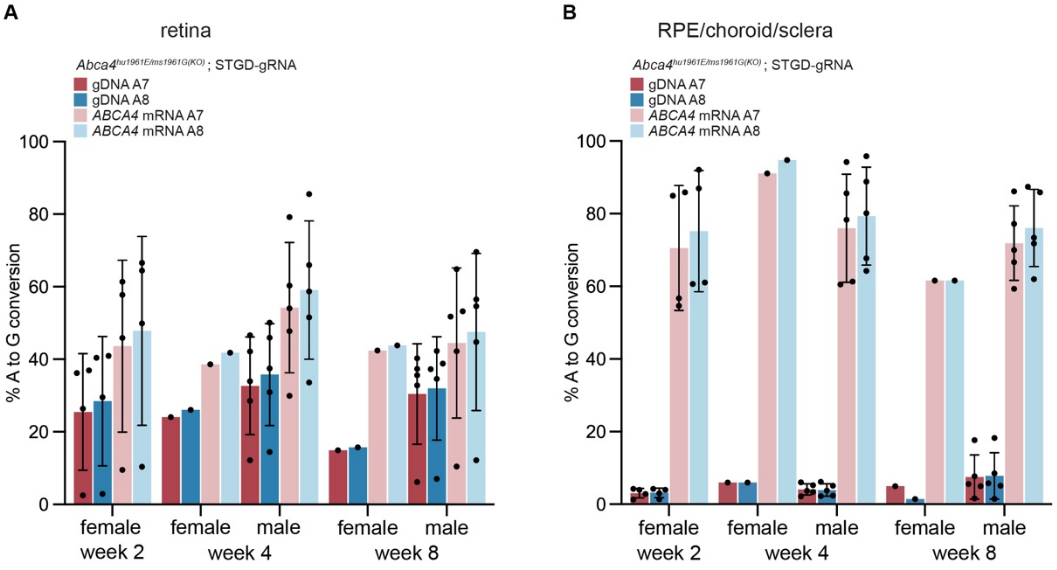

Source Data Fig 5A.

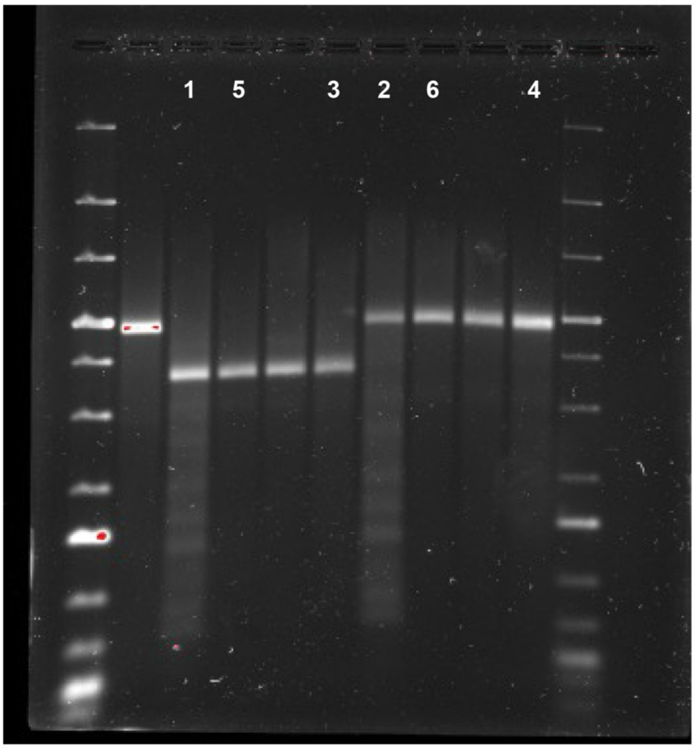

1: AAV5-v1-SABE1(N)

2: AAV5-v1-SABE1(C)

3: AAV5-v2-SABE1(N)

4: AAV5-v2-SABE1(C)

5: AAV9-PHP.eB-SABE1(N)

6: AAV9-PHP.eB-SABE1(C)
