# Supplementary material for: High-efficiency base editing in the retina in primates and human tissues
Source: Nat Med. 2025 Jan 8;31(2):490–501. doi: 10.1038/s41591-024-03422-8 (PMC11835749; doi:10.1038/s41591-024-03422-8)
Supplement: Supplementary file 2 — Reporting Summary [file 41591_2024_3422_MOESM2_ESM.pdf]

nature portfolio

Corresponding author(s): Botond Roska, Bence György

Last updated by author(s): 07.11.2024

Reporting Summary

Nature Portfolio wishes to improve the reproducibility of the work that we publish. This form provides structure for consistency and transparency in reporting. For further information on Nature Portfolio policies, see our Editorial Policies and the Editorial Policy Checklist.

Statistics

For all statistical analyses, confirm that the following items are present in the figure legend, table legend, main text, or Methods section.

n/a	Confirmed	
☐	☒	The exact sample size (*n*) for each experimental group/condition, given as a discrete number and unit of measurement
☐	☒	A statement on whether measurements were taken from distinct samples or whether the same sample was measured repeatedly
☐	☒	The statistical test(s) used AND whether they are one- or two-sided<br>*Only common tests should be described solely by name; describe more complex techniques in the Methods section.*
☐	☒	A description of all covariates tested
☐	☒	A description of any assumptions or corrections, such as tests of normality and adjustment for multiple comparisons
☐	☒	A full description of the statistical parameters including central tendency (e.g. means) or other basic estimates (e.g. regression coefficient) AND variation (e.g. standard deviation) or associated estimates of uncertainty (e.g. confidence intervals)
☐	☒	For null hypothesis testing, the test statistic (e.g. *F*, *t*, *r*) with confidence intervals, effect sizes, degrees of freedom and *P* value noted<br>*Give P values as exact values whenever suitable.*
☒	☐	For Bayesian analysis, information on the choice of priors and Markov chain Monte Carlo settings
☒	☐	For hierarchical and complex designs, identification of the appropriate level for tests and full reporting of outcomes
☒	☐	Estimates of effect sizes (e.g. Cohen's *d*, Pearson's *r*), indicating how they were calculated

Our web collection on statistics for biologists contains articles on many of the points above.

Software and code

Policy information about availability of computer code

Data collection	In this study, no software for data collection was used.
Data analysis	Statistical analysis was performed in R (version 4.2.2). Image analysis was performed in ImageJ (version 2.14.0/1.54f) with the Plugin 'Cell counter'. For targeted deep-sequencing analysis, we used the following softwares as stated in the method section: Illumina blc2fastq (v2.20.0.422), Trimmomatic (v0.39), Bowtie2 (v2.35), Samtools (v1.9), bam-readcounts tool (https://github.com/genome/bam-readcount). Initial in silico identification of candidate off-target sites was performed by running Cas-OFFinder v2.4.

For manuscripts utilizing custom algorithms or software that are central to the research but not yet described in published literature, software must be made available to editors and reviewers. We strongly encourage code deposition in a community repository (e.g. GitHub). See the Nature Portfolio guidelines for submitting code & software for further information.

Data

Policy information about availability of data

All manuscripts must include a data availability statement. This statement should provide the following information, where applicable:

- Accession codes, unique identifiers, or web links for publicly available datasets
- A description of any restrictions on data availability
- For clinical datasets or third party data, please ensure that the statement adheres to our policy

The data and materials availability section of the manuscript states that all data are available in the manuscript or the supplementary materials. We used the

GRCm38 mouse genome (https://www.ncbi.nlm.nih.gov/datasets/genome/GCF_000001635.20/), the Macaca_fascicularis_5.0 (GCF_000364345.1) macaque genome and the GRCh38 human genome (https://www.ncbi.nlm.nih.gov/datasets/genome/GCF_000001405.26/) for alignment. The sequencing data is available in the SRA database under accession number PRJNA1170171.

Research involving human participants, their data, or biological material

Policy information about studies with human participants or human data. See also policy information about sex, gender (identity/presentation), and sexual orientation and race, ethnicity and racism.

Reporting on sex and gender	Specification about human donor sex: samples in Figure 1F, 2D, 2E, 2G and 6 were from male donors and samples in Figure 2F, Extended Data Figure 7D and 7E, Extended Data Figure 8A,C and Extended Data Figure 9A,C were from female donors.
Reporting on race, ethnicity, or other socially relevant groupings	This information was not available from human donors.
Population characteristics	All human samples are derived from European population, but race and information on population charactertistics were not collected.
Recruitment	Human retina samples were collected in Basel (Basel University, Department of Ophthalmology) and in Budapest (Semmelweis University, Department of Ophthalmology)
Ethics oversight	All experimental protocols were approved by the local ethics committees (ethical permit numbers: Budapest: ETT TUKEB 34851-2/2018/EKU and ETT TUKEB IV/5645-1/2021/EKU, Basel: EKNZ 2021-01773).

Note that full information on the approval of the study protocol must also be provided in the manuscript.

Field-specific reporting

Please select the one below that is the best fit for your research. If you are not sure, read the appropriate sections before making your selection.

☒ Life sciences ☐ Behavioural & social sciences ☐ Ecological, evolutionary & environmental sciences

For a reference copy of the document with all sections, see nature.com/documents/nr-reporting-summary-flat.pdf

Life sciences study design

All studies must disclose on these points even when the disclosure is negative.

Sample size	Sample size is given throughout the manuscript. We performed the experiment throughout the study at least in triplicates to allow for statistical comparison. Sample sizes are consistent with standards of the field, which have consistently provided sufficient power to characterize editing efficiency (Levy JM et al. Nat Biomed Eng, Davis JR et al. Nat Biomed End). For NHP studies, we planned the numbers of eyes based on previous data (Juettner J et al. Nat Neurosci 2019) and power calculation. In general we calculated that using 4 eyes in one group we can detect a minimum of 25% difference (considered relevant) in base editing efficiency between two different experimental conditions with a power set to 0.8 and a P value of 0.05. Therefore in our hands, 4 eyes in one group is considered for a well-powered study and therefore minimum 4 eyes are included in the key experimental cohorts.
Data exclusions	Three out of 36 NHP eyes were excluded from the study. These eyes were excluded because OCT imaging showed no subretinal blebs and are listed in Supplementary Table 5.
Replication	All experiments represent independent biological replicates (independent organoids, independent human retina or RPE/choroid tissues from one donor per experiment, different eyes from different animals in the case of in vivo injections, Supplementart Table S4 and S5).
Randomization	Organoids were randomly selected for experiment. Human retina tissues and RPE/choroid tissues were obtained randomly without considering the retinal location. Mice were randomly allocated to group, with respect to sex and age. NHPs were randomly allocated with respect to age.
Blinding	Sequencing experiments were not performed in a blinded way, as all samples were processed through the exact same analytical and computational pipeline, avoiding any potential bias. Analysis of potential phenotype was performed using coded samples by blinded investigators.

Reporting for specific materials, systems and methods

We require information from authors about some types of materials, experimental systems and methods used in many studies. Here, indicate whether each material, system or method listed is relevant to your study. If you are not sure if a list item applies to your research, read the appropriate section before selecting a response.

Materials & experimental systems

n/a	Involved in the study
☐	☒ Antibodies
☐	☒ Eukaryotic cell lines
☒	☐ Palaeontology and archaeology
☐	☒ Animals and other organisms
☒	☐ Clinical data
☒	☐ Dual use research of concern
☒	☐ Plants

Methods

n/a	Involved in the study
☒	☐ ChIP-seq
☒	☐ Flow cytometry
☒	☐ MRI-based neuroimaging

Antibodies

Antibodies used

Primary antibodies: Rabbit polyclonal anti-ARR3: Sigma-Aldrich, PRODUCT #: HPA063129, 1:1000 dilution, Goat anti-ARR3: Novus, PRODUCT #: NBP1-37003, 1:200 dilution, Mouse monoclonal anti-Ceramide; clone MID 15B4: Enzo Life Sciences, PRODUCT #: ALX-804-196-T050, 1:100 dilution, Rabbit monoclonal Anti-CRISPR-Cas9; clone EPR18991: Abcam, PRODUCT #: ab189380, 1:200 dilution, Mouse monoclonal Anti-CRISPR-Cas9; clone 4G10: Diagenode, PRODUCT #: C15200216, 1:400 dilution, Goat polyclonal anti-Nanog: R&D systems, PRODUCT #: AF1997, 1:200 dilution, Rabbit monoclonal anti-Oct4: Abcam, PRODUCT #: Ab181557, 1:100 dilution, Mouse monoclonal anti-RHO; clone 1D4: Sigma-Aldrich, PRODUCT #: R5403, 1:500 dilution, Rabbit polyclonal anti-SOX2: Millipore, PRODUCT #: AB5603, 1:200 dilution, Mouse monoclonal anti-SSEA4: Invitrogen, PRODUCT #: 414000, 1:200 dilution, Rabbit polyclonal anti-ZO1: Abcam PRODUCT #:ab216880, 1:400 dilution, Mouse monoclonal anti-ZO1: Thermo Fisher Scientific, PRODUCT #: MA3-39100-A488, 1:100 dilution.
Secondary antibodies: Alexa Fluor 488 donkey anti-rabbit IgG: Invitrogen, PRODUCT #: A21206, 1:500 dilution, Alexa Fluor 488 Phalloidin: Thermo Fisher Scientific, PRODUCT #: A12379, 1:200 dilution, Alexa Fluor 488-conjugated AffiniPure F(ab')2 Fragment Donkey anti-mouse IgG: Jackson ImmunoResearch, PRODUCT #: 715-546-150, 1:500 dilution, Alexa Fluor 555 donkey anti-rabbit IgG: Thermo Fisher Scientific, PRODUCT #: A31572, 1:800 dilution, Alexa Fluor 568 donkey anti-mouse IgG, Invitrogen, PRODUCT #: A10037, 1:500 dilution, Alexa Fluor 647 donkey anti-rabbit IgG, Invitrogen, PRODUCT #: A31573, 1:500 dilution, Alexa Fluor 647 donkey anti-goat IgG, Invitrogen, PRODUCT #: A21447, 1:500 dilution.

Validation

All antibodies are from commercial sources and we have not performed validation. HPA063129 was validated in human retina by Sigma-Aldrich using immunohistochemistry. NBP1-37003 was validated in human retina by Novus Biologicals using immunohistochemistry. No validation information is provided for ALX-804-196-T050. ab189380 was validated in transfected HEK-293 cells by Western blot. C15200216 was validated in transfected HEK-293 cells by Western blot and immunofluorescence. AF1997 was validated using iPSC lysates by Western blot. Ab181557 was validated using NCCIT cell lysates by immunprecipitation. No validation information is provided for R5403. AB5603 was validated in mESCs by Western blot and human stem cells by immunofluorescence. 414000 was validated in various stem cells using multiple methods, including flow cytometry and immunofluorescence. ab216880 was validated using multiple cell types using Western blot. MA3-39100-A488 antibody was validated using multiple cell lines (including knock-out) by immunofluorescence.

Eukaryotic cell lines

Policy information about cell lines and Sex and Gender in Research

Cell line source(s)

iPSC line 01F49i-N-B7 (short name: F49B7) 01F49i-N-B7 - female
iPSC line iPS(IMR90)-4-DL-01 (short name: IMR90) WiCell iPS(IMR90)-4-DL-01 - female
HEK293T cells (American Type Cell Culture Collection (ATCC), CRL-3216)
iPSC-control 1 - female
iPSC-control 2 - male
iPSC- patient 1- female
iPSC- patient 2 - male

Authentication

None of the cell lines were authenticated, but they are from primary sources.

Mycoplasma contamination

We regularly confirmed the lack of mycoplasma contamination.

Commonly misidentified lines
(See ICLAC register)

None used.

Animals and other research organisms

Policy information about studies involving animals; ARRIVE guidelines recommended for reporting animal research, and Sex and Gender in Research

Laboratory animals

C57BL/6J wild-type mice, B6.129S-Abca4tm1Ght/J and Abca4hu1961E/ms1961G(KO) were used. Both sexes were included in the study in a randomized way. Animals were between 11 and 22 weeks at the time of injection.Details are listed in Supplementary Table 4. 21 macaca fascicularis, aged 6- to 15 years old at the time of injection (9 animals from Mauritius and 2 from China) were used in the study. All animals were females.Details are listed in Supplementary Table 5.

Wild animals	The study did not involve wild animals.
Reporting on sex	The study included both male and female mice as indicated in Supplementary Table 4. All individual data points regarding male and female mice, are included in the SRA submission. We included only female non-human primates, this is indicated in the Abstract and Supplementary Table 5.
Field-collected samples	The study did not involve field-collected samples.
Ethics oversight	All mice experiments and procedures were approved by the local ethics committee (permit number: 3048/31896, Kantonales Veterinäramt Basel-Stadt).All non-human primate protocols were approved by the French Ministry of Higher Education and Research (permit number: APAFIS#27357-2020092811266511_v2 (28/12/2020)).

Note that full information on the approval of the study protocol must also be provided in the manuscript.

Plants

Seed stocks	*Report on the source of all seed stocks or other plant material used. If applicable, state the seed stock centre and catalogue number. If plant specimens were collected from the field, describe the collection location, date and sampling procedures.*
Novel plant genotypes	*Describe the methods by which all novel plant genotypes were produced. This includes those generated by transgenic approaches, gene editing, chemical/radiation-based mutagenesis and hybridization. For transgenic lines, describe the transformation method, the number of independent lines analyzed and the generation upon which experiments were performed. For gene-edited lines, describe the editor used, the endogenous sequence targeted for editing, the targeting guide RNA sequence (if applicable) and how the editor was applied.*
Authentication	*Describe any authentication procedures for each seed stock used or novel genotype generated. Describe any experiments used to assess the effect of a mutation and, where applicable, how potential secondary effects (e.g. second site T-DNA insertions, mosiacism, off-target gene editing) were examined.*
