# Supplementary figures and images for: High-efficiency base editing in the retina in primates and human tissues
Source: Nat Med. 2025 Jan 8;31(2):490–501. doi: 10.1038/s41591-024-03422-8 (PMC11835749; doi:10.1038/s41591-024-03422-8)

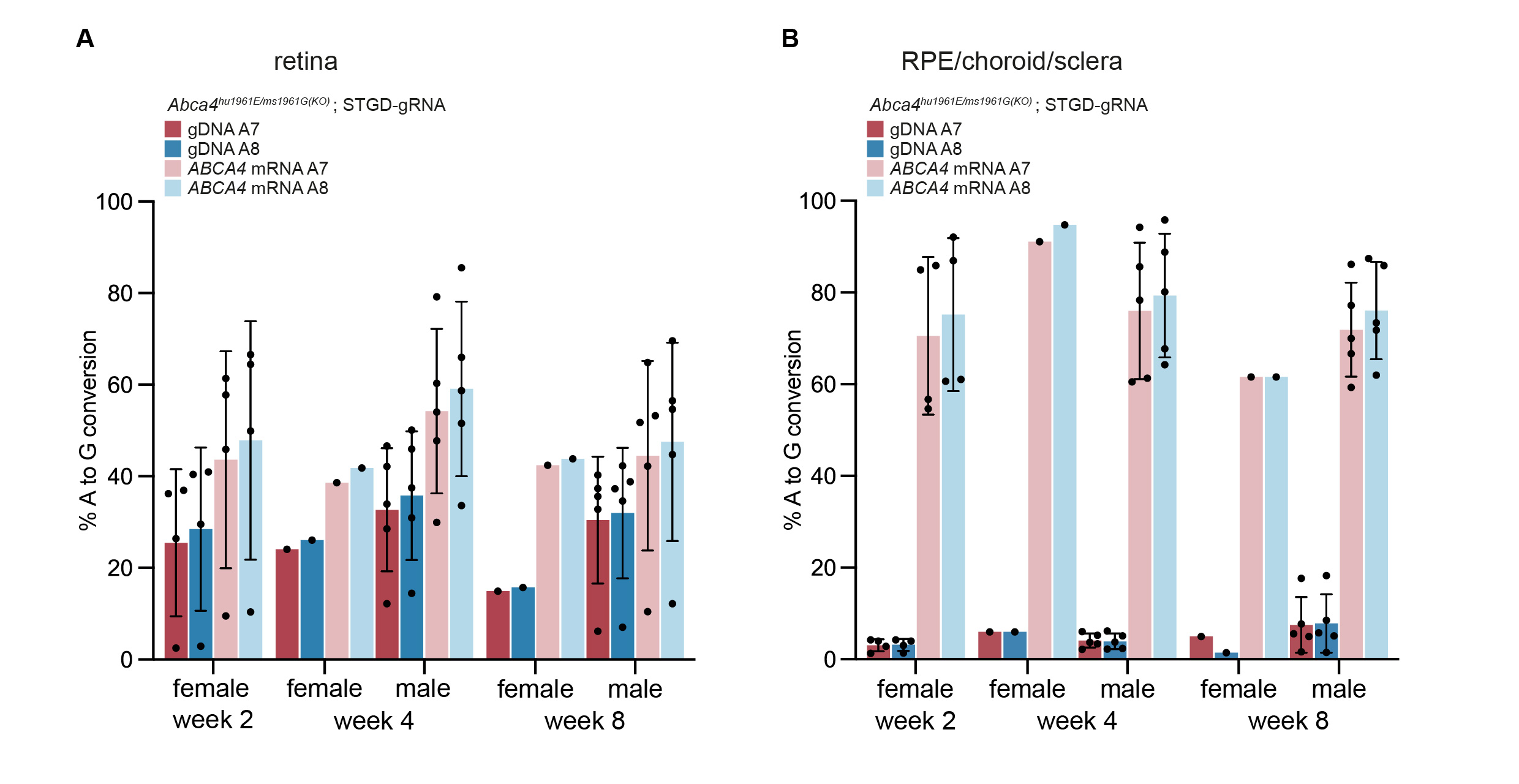

Supplement: Supplementary file 4 — Base-editing results in mice from Fig. 3c segregated by sex. [file 41591_2024_3422_MOESM4_ESM.jpg]

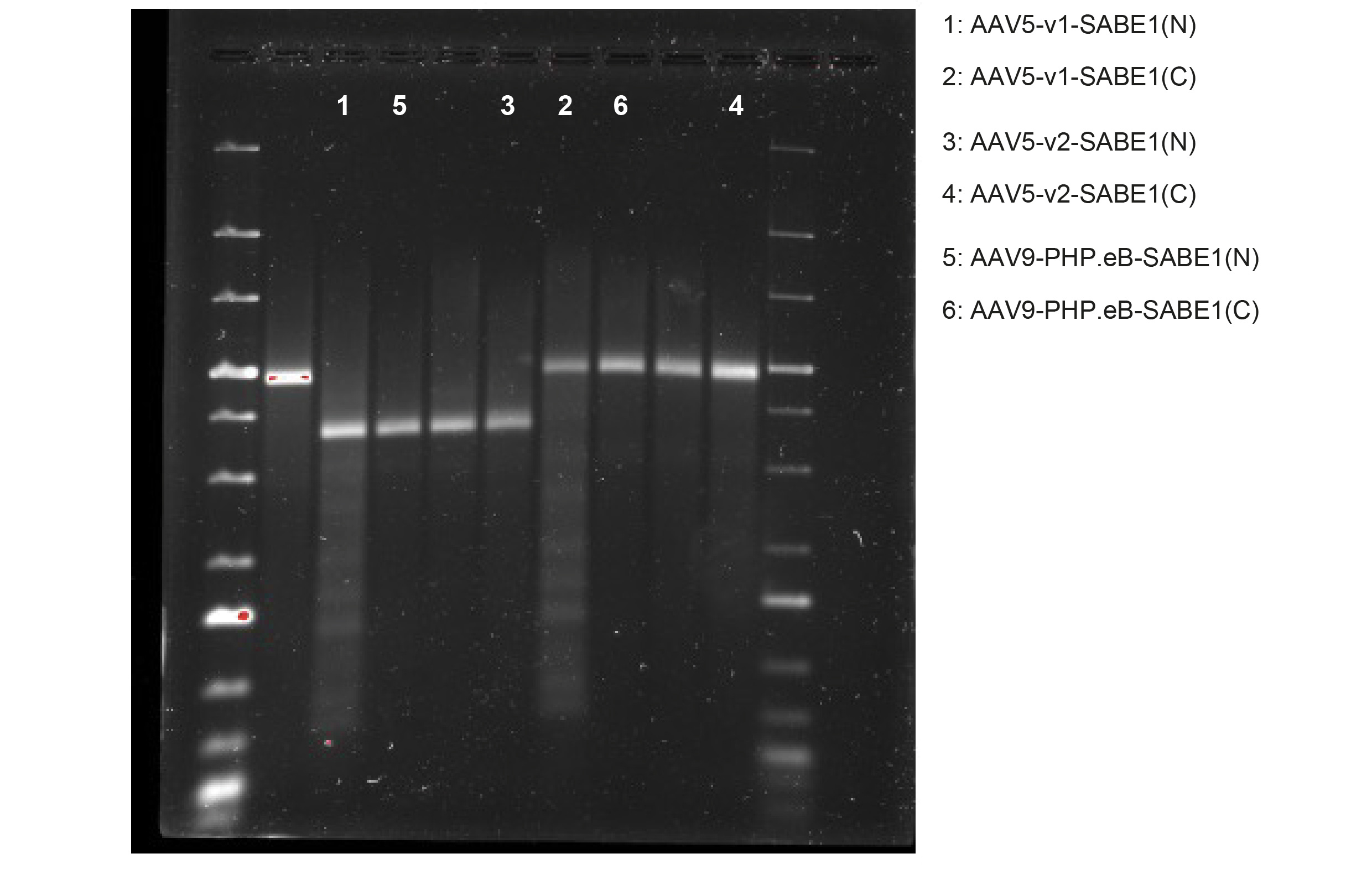

Supplement: Supplementary file 5 — Unprocessed alkaline gel electrophoresis image. [file 41591_2024_3422_MOESM5_ESM.jpg]
